# Comparative Transcriptome Analysis to Reveal Differentially Expressed Cytochrome P450 in Response to Imidacloprid in the Aphid Lion, *Chrysoperla zastrowi sillemi* (Esben-Petersen)

**DOI:** 10.3390/insects13100900

**Published:** 2022-10-03

**Authors:** Jyoti Pathak, Gandhi Gracy Ramasamy, Aditi Agrawal, Subhi Srivastava, Bhusangar Raghavendra Basavaarya, Mohan Muthugounder, Venugopal Kundalagurki Muniyappa, Pratheepa Maria, Anil Rai, Thiruvengadam Venkatesan

**Affiliations:** 1 Division of Genomic Resources, ICAR-National Bureau of Agricultural Insect Resources, P. Bag No. 2491, H.A. Farm Post Bellary Road, Hebbal, Bangalore 560024, India; 2 Centre for Agricultural Bioinformatics, Indian Agricultural Statistical Research Institute, Pusa, New Delhi 110012, India

**Keywords:** *Chrysoperla zastrowi sillemi* (CZS), Integrated Pest Management (IPM), cytochrome P450 (CYP), imidacloprid, resistant, transcriptome

## Abstract

**Simple Summary:**

*Chrysoperla zastrowi sillemi* (CZS) is a generalist predator of arthropod pests in different crops and is distributed in wide geographical regions. Being a natural predator, CZS shares an ecological niche with the pests and is exposed to several groups of pesticides including imidacloprid. Due to continuous exposure, it has developed resistance to several insecticides. Transcriptomes of imidacloprid-resistant and susceptible strains have been generated and compared for expression differences. From the transcriptome, sequences belonging to the CYP gene family have been mined for their nomenclature and classification into the four CYP clans. Putative functions of the CYP families in CZS have been identified by phylogenetic analysis including CYP sequences from *Drosophila* and *Tribolium*. Further, differential expressions of CYP genes have been validated using qRT-PCR. We found nine CYP genes to be downregulated and one to be upregulated after imidacloprid treatment. The information from current study can be exploited for the effective implementation of IPM as it aims at sustainable and eco-friendly crop yield improvement.

**Abstract:**

The aphid lion, *Chrysoperla zastrowi sillemi* (Neuroptera: Chrysopidae) is a highly effective beneficial predator of many agricultural pests and has developed resistance to several insecticides. Understanding the molecular mechanism of insecticide resistance in the predators is crucial for its effective application in IPM programs. Therefore, transcriptomes of imidacloprid-resistant and susceptible strains have been assessed using RNA-seq. Cytochrome P450 is one of the important gene families involved in xenobiotic metabolism. Hence, our study focused on the CYP gene family where mining, nomenclature, and phylogenetic analysis revealed a total of 95 unique CYP genes with considerable expansion in CYP3 and CYP4 clans. Further, differential gene expression (DGE) analysis revealed ten CYP genes from CYP3 and CYP4 clans to be differentially expressed, out of which nine genes (CYP4419A1, CYP4XK1, CYP4416A10, CYP4416A-fragment8, CYP6YL1, CYP6YH6, CYP9GK-fragment16, CYP9GN2, CYP9GK6) were downregulated and one (CYP9GK3) was upregulated in the resistant strain as compared to the susceptible strain. Expression validation by quantitative real-time PCR (qRT-PCR) is consistent with the DGE results. The expansion and differential expression of CYP genes may be an indicator of the capacity of the predator to detoxify a particular group of insecticides.

## 1. Introduction

Common green lacewing, *Chrysoperla zastrowi sillemi* (Neuroptera: Chrysopidae), is an important natural predator of aphids, coccids, mealybugs, thrips, psyllids, and whiteflies. They also feed on the eggs and larvae of many lepidopteran pests and mites occurring on various crops [1]. Green lacewings are of main interest in biocontrol programs because of their polyphagous nature and high predatory potential [2]. They can also be mass-reared on factitious host insects for commercialization [3]. Furthermore, they have developed resistance to several widely used insecticides which can be an added advantage for their use in Integrated Pest Management (IPM) programs [4]. The Indian green lacewings were earlier known as *Chrysoperla carnea* (Stephens), later taxonomically confirmed as *Chrysoperla zastrowi sillemi* (Esben-Petersen) [5] and are successfully used as a commercial biological control in India. Several studies have confirmed the high predatory potential of its larvae both in the field as well as under laboratory conditions [6,7] and their high resistance potential against the commonly used insecticides [8,9]. 

Neurotoxins of different modes of action are the most common form of insecticides used nowadays for the control of insect pests, primarily hemipterans. One of the most frequently used insecticides in the class “neonicotinoids” is imidacloprid [10]. Due to the excessive use of imidacloprid, insects developed resistance mainly via enhanced cytochrome P450 mediated detoxification. Several studies have confirmed the upregulation of CYP genes in insects in response to imidacloprid [11,12,13,14]. With the increasing transcriptomic and genomic information, cytochrome P450 continues to be the largest enzyme family in animals, plants, and fungi [15]. Cytochrome P450s are ubiquitously expressed and are involved in a wide array of functions in insects. They are involved in a myriad of catalytic functions including the metabolism of endogenous and exogenous compounds. The heme-thiolate group makes them monooxygenase, capable of adding oxygen to a great variety of substrates for hydroxylation, epoxidation, and dealkylations [16]. They are also the first in conferring metabolic resistance to the insecticide [17,18]. Cytochrome P450 protects insects from neurotoxins either via gene expansion or increased expression [19].

Transcriptome sequencing is an effective way for functional and differential expression analysis and is widely used to increase the information of transcribed regions and to discover the function of novel genes [20,21,22]. Hence, to understand the role of CYP genes in the resistance mechanism in CZS, a transcriptome-based approach was adopted, and differential gene expression analysis was carried out to reveal resistance-relevant CYP genes. CYPs are not only responsible for the metabolism of xenobiotics but are also involved in various other functions in insects, viz., synthesis and degradation of ecdysteroids, juvenile hormones, odorants, and pheromones which are essential for the insect growth, development, reproduction, and survival [23]. Therefore, to enhance our knowledge about the CYP genes, we mined the protein sequences belonging to the CYP family and classified them into CYP2, mitochondrial, CYP3, and CYP4 clans. Based on the phylogenetic analysis most likely orthologs and paralogs have been identified in CZS.

## 2. Materials and Methods

### 2.1. Insect Collection and Laboratory Maintenance

**Figure 1:**
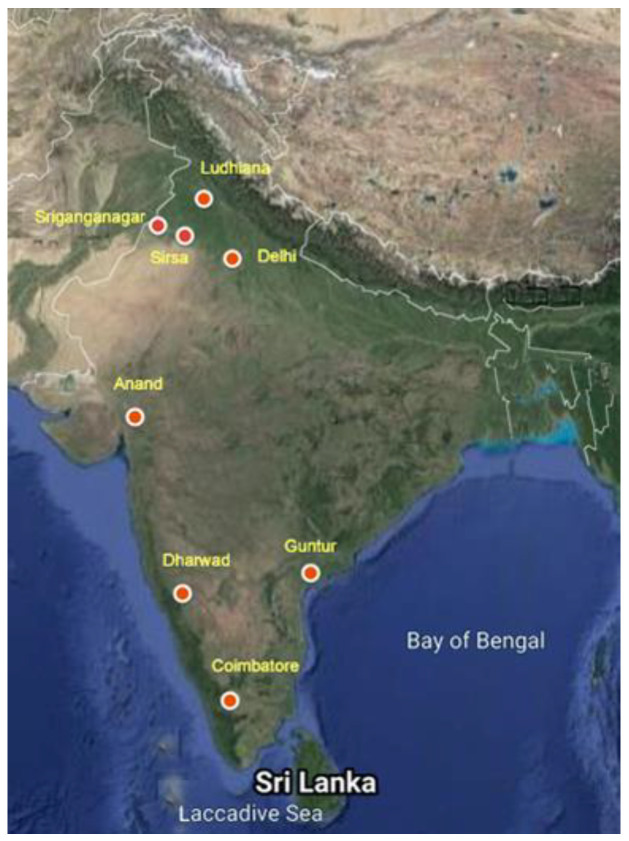
Different locations of collection of CZS populations from India.

Eight populations of CZS (~100 larvae/adults) were collected in 2013–2014 from the cotton fields in eight states of India, viz., Coimbatore (Tamil Nadu), Anand (Gujarat), Delhi, Sirsa (Haryana), Sriganganagar (Rajasthan), Guntur (Andhra Pradesh), Dharwad (Karnataka) and Ludhiana (Punjab) (Figure 1). The field-collected populations were maintained individually and bioassays were performed with imidacloprid to calculate LC_50_ to find the most resistant strain. The Sriganganagar population with the observed maximum LC_50_ was selected for further resistance development and was continuously sprayed with imidacloprid 17.8% Soluble Liquid (SL) 0.2 mL/liter one time in a single lifecycle of the insect up to 15 generations. This population was considered imidacloprid-resistant and was used for further transcriptome analysis.

A laboratory population of CZS originally maintained for the past 11 years at ICAR-National Bureau of Agricultural Insect Resources (NBAIR), Bangalore, India, without exposure to insecticides for 125 generations was used in the study as a susceptible strain. The larvae and adult populations of CZS were maintained as described by Venkatesan et al. [24].

### 2.2. Dose Mortality Bioassays

Based on the field recommended dosage of imidacloprid i.e., 17.8 SL (0.2 mL/liter), the following concentrations were used for the bioassay studies: 0.025, 0.05, 0.10, 0.20, 0.40, 0.80, 1.60 mL/liter of water. The pesticide doses were applied on 3-day-old larvae of CZS in the weight range of 0.8 to 1.2 mg by using topical assays [25]. Imidacloprid was tested with seven concentrations initially and the number of concentrations was further increased to 3.2 and 6.4 mL/liter of water as we did not obtain 50% mortality. Such insecticide-treated larvae were provided with factitious host insect *Corcyra cephalonica* eggs and were maintained in a plant growth chamber at a temperature (25 ± 2°C) and RH (80%). Control larvae were treated with distilled water alone. At least 30 larvae were used for each concentration as well as in control. The larval mortality was recorded after 48 h and the larvae were considered dead if they did not move when prodded. The LC_50_ of different populations was worked out and the population collected from Sriganaganagar recorded the highest LC_50_ compared to other populations and was considered a resistant strain. The selected resistant strain was further exposed to imidacloprid @ 0.2 mL/liter continuously for 15 generations and the LC_50_ was worked out.

### 2.3. RNA Isolation, Library Preparation, and Sequencing

RNA was isolated from 3rd instar larvae (ten per replicate) from both resistant and susceptible strains using the TRIzol (Invitrogen, Carlsbad, CA, NA, USA) method [26]. Two biological replicates were taken for each condition. RNA quality was checked and quantified with Nanodrop (Jenway 7415 nano-micro-volume spectrophotometer). The integrity of RNA was checked using the Bioanalyzer 2100 system (Agilent, Santa Clara, CA, NA, USA) and on 1% agarose gel.

Sequencing libraries were constructed using NEBNext^®^ Ultra™ RNA Library Prep Kit for Illumina^®^ by Nucleome Informatics Pvt. Ltd. Hyderabad, India as per the manufacturer’s protocol (Illumina). mRNA enrichment was performed using oligo (dT) magnetic beads and fragmented using divalent cations. First-strand cDNA synthesis was carried out using random primers and reverse transcriptase while DNA Polymerase1 and RNaseH were used for second-strand synthesis followed by polyadenylation, ligation to adapters, and enrichment by PCR. Sequencing was carried out on Illumina Hiseq 2500 system by Nucleome Informatics Pvt. Ltd. Hyderabad, India after assessing the library quality using Agilent Bioanalyzer 2100.

### 2.4. Pre-Processing of Raw Reads, De Novo Assembly, and Quality Assessment

The quality of raw sequence data was checked with FastQC v0.11.8 [27]. Adapters and low-quality reads were removed using Trimmomatic v0.36 software [28] with the following parameters: TruSeq3PE-2.fasta adapters (Illumina) with the palindromic algorithm, trim read when the average base quality in 4-base sliding window falls below 25. A minimum length for a read was set to 36 bp. The raw sequence data has been submitted to the NCBI Sequence Read Archive (SRA) under accession numbers SRR3820550 SRR3820549 SRR3820548 SRR3820547. The raw reads were assembled de novo using Trinity v2.5.1 [29] with default parameters, normalization of reads to 50X coverage, and k-mer length of 25. CD-HIT-EST v4.6.8 [30] was used for clustering assembled transcripts at 98% identity to reduce the redundant transcripts. To check the quality of our assembly, we followed several characterization methods. First, the raw reads were aligned back to the generated transcriptome assembly using Bowtie2 [31] and Samtools [32] to check the percentage mapping of the reads. Among the other assembly quality assessment, Benchmarking Universal Single-Copy Orthologs (BUSCO v3.1.0) [33] was performed to measure the completeness and contiguity of the transcriptome against 1066 arthropod (https://busco.ezlab.org/v2/datasets/arthropoda_odb9.tar.gz, accessed on 5 February 2020) BUSCO sets.

### 2.5. Transcriptome Annotation

To discriminate between valid transcript sequences and incorrectly assembled sequences, a potential coding region was identified in the generated transcriptome assembly from the longest ORF with a minimum length of 200 bp using TransDecoder (v5.5.0, http://transdecoder.github.io, accessed on 7 February 2020). Trinotate pipeline (v3.2.0, http://trinotate.github.io, accessed on 10 February 2020) was followed for annotation in which the sequences were searched for homologies to SwissProt using NCBI-BLASTx and BLASTp. Predicted protein sequences were also searched for conserved domain using Protein family (Pfam) database with HMMER (v3.2.1, http://hmmer.org/, accessed on 15 February 2020), for signal peptides with signalP v4 (http://www.cbs.dtu.dk/cgi-bin/nph-sw_request?signalp, accessed on 20 February 2020) and transmembrane helices with TmHMM v2 software (http://www.cbs.dtu.dk/cgi-bin/nph-sw_request?tmhmm, accessed on 25 February 2020). Trinotate leverages various annotation databases viz., Gene Ontology (GO), evolutionary genealogy of genes: Non-supervised Orthologous Groups (eggNOG) and Cluster of Orthologous Groups (COG), Eukaryotic Orthologous Groups of proteins (KOG) and Kyoto Encyclopedia of Genes and Genomes Ortholog (KEGG) databases.

### 2.6. Transcript Abundance and Differential Gene Expression

Clean reads from all the four libraries were mapped back to the assembled transcriptome using Bowtie2. Expression levels of transcripts were assessed by the alignment-based quantification protocol implemented in RSEM (RNA-Seq by Expectation Maximization) [34]; http://deweylab.github.io/RSEM/, accessed on 10 March 2020) using the script align_and_estimate_abundance.pl in Trinity. It gives an estimate of the count of RNA-seq fragments derived from each sequence both at the transcript and gene levels. The read counts from all of the samples were combined into a matrix and normalized using the TMM method. Normalized expression metrics are reported as FPKM (fragments per kilobase transcript length per million fragments mapped) or TPM (transcripts per million). The normalized values take into account the length of the transcript, the number of reads that mapped to the transcript, and the total number of reads mapped to any transcript implemented in abundance_estimate_to_matrix.pl script in Trinity. Based on the estimated fragment count at the isoform level, differentially expressed transcripts were identified using the EdgeR (Empirical Analysis of Digital Gene Expression in R) method [35], implemented by the script run_DE_analysis.pl.

### 2.7. Identification and Phylogenetic Classification of Cytochrome P450 Sequences

The CYP nomenclature committee is dedicated to naming the sequences by similarity search and domain analysis with the percentage cutoff of 40 for a family and 55 for a subfamily [36]. From the Pfam (PF00067) search of transcriptome data, CYP sequences were identified and submitted for naming to the approved CYP nomenclature committee (Dr. David Nelson, Univ. Tennessee). A total of 198 CYP sequences of at least 350 amino acid lengths were taken for phylogenetic analysis. These contain different isoforms of the CYP genes. After removing the duplicates, 160 CYP sequences were submitted to GenBank under accession numbers ON646299-ON646458 (Sequences are also provided in the File S1). Sequence homology of CZS CYP was searched using protein sequences from *D. melanogaster* [37] and *T. casteneum* [38]. The annotated P450 sequences from the representative insect species were obtained from the NCBI database. A combined phylogenetic tree was generated by using the 131 sequences from *T. castaneum*, 77 sequences from *D. melanogaster*, and 198 CZS CYP sequences, and evolutionary aspects were analyzed. Multiple sequence alignment was carried out using Clustal W and NJ-tree was constructed with 1000 bootstrap in MEGA 7 software [39].

### 2.8. Total RNA Isolation and Reverse Transcription Quantitative Real-Time PCR (qRT-PCR)

Real-time PCR primers were designed using IDT Primer Quest Tool. Oligo synthesis was carried out by Bioserve Biotechnologies (www.bioserveindia.com, accessed on 22 August 2021), Bangalore, India. For expression induction, the 3rd instar larvae were exposed to imidacloprid, and the survivals were collected at the 12th hour after imidacloprid application. Three biological replicates with 10 larvae in each tube were taken to obtain a sufficient amount of RNA. Samples were stored at −80 °C until RNA extraction by the TRIZOL method. One microgram of total RNA was used to convert into cDNA after removing genomic DNA using PrimeScript^TM^ RT Reagent Kit with gDNA Eraser (cat. # RR047A), TaKaRa Biotechnology, China. TB Green™ Premix Ex Taq™ II (TliRNaseH Plus) (cat. # RR820A) from TaKaRa Biotechnology, China was used for running qRT-PCR on CFX96 Touch^TM^ Real-Time PCR Detection System (Bio-Rad, Hercules, CA, NA, USA). The real-time experiment was performed with three biological replicates and three technical replicates for each biological replicate. The thermal cycling conditions were 45 cycles of 95 °C for 30 s, 58 °C for 30 s, and 72 °C for 30 s. The 28S reference gene was used as an internal control. Relative gene expression was determined with the 2^−ΔΔCT^ method [40]. qRT-PCR was carried out according to MIQE (minimum information for Q-PCR experiment) guidelines [41].

## 3. Results

### 3.1. Bioassay

Among the 8 fields and one susceptible population of tested *C. zastrowi sillemi* (CZS) with imidacloprid, the Sriganganagar population recorded a maximum LC_50_ (1.08 mL/liter) with a resistance ratio (RR) of 12 (Table 1). The Sriganganagar population was further exposed to imidacloprid (0.2 mL/liter) for 15 generations to the final LC_50_ of 2.24 mL/liter and a resistance ratio of 56 compared to the susceptible strain (0.04 mL/liter) (Table 2) was obtained.

### 3.2. RNA Sequencing and Quality Control

Illumina sequencing from all four libraries gave a total of 101,253,143 million high-quality reads with an average GC content of 33.75%. Approximately 90% of the reads survived after adapter removal and quality filter with lengths ranging from 36–150 bp. FastQC analysis after trimming showed high-quality scores (Q > 30) from all four samples.

### 3.3. De novo Transcriptome Assembly and Quality Assessment

**Figure 2:**
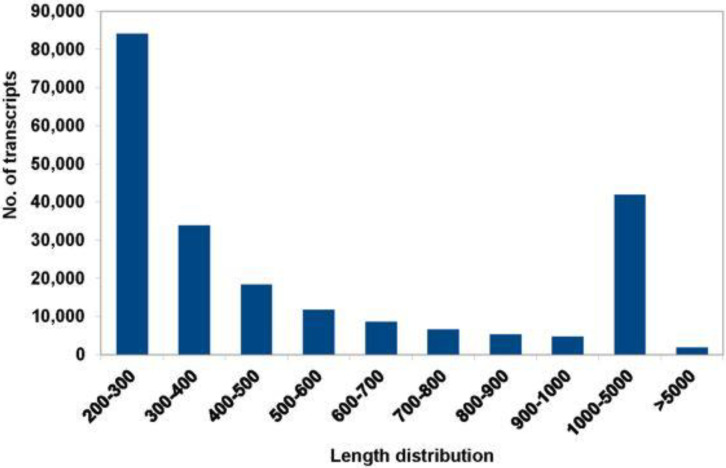
Length distribution of de novo assembled transcripts. *y*-axis represents the no. of transcripts for the length distribution on the *x*-axis.

A total of 162,945,182 bases were assembled into 146,051 genes and 216,595 transcripts with N50 of 1391 and a median contig length of 364. The size distribution indicated the lengths of 43,781 contigs to be longer than 1000 bp (Figure 2). More than 85% of the reads aligned concordantly back to the assembly from all four libraries. Among the 1066 arthropod BUSCO, 1017 (95.4%) were found to be complete (of which 395 [37.05%] were single-copy and 622 [58.35%] were duplicated), 36 (3.38%) were fragmented, and 13 (1.22%) were missing from the assembly.

### 3.4. Transcriptome Annotation

A total of 10,712 distinct Transdecoder predicted proteins and 10,085 distinct transcripts were annotated to the Swiss-Prot database using BLASTp and BLASTx, respectively. After annotation, 10,926 proteins were assigned to Pfam domains, TmHMM (8909), eggNOG or COG (52,352), KEGG (34,158), GO-BLASTx (50,119), GO-BLASTp (35,754), GO-Pfam (24,565). Of the total predicted transcripts 10119 unique sequences were obtained, out of which 9998 were assigned to at least one protein family.

**Figure 3:**
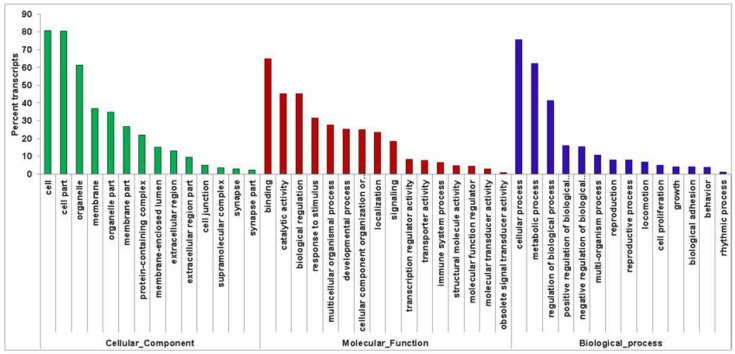
Gene Ontology (GO) classification of assembled transcripts from the GO-BLASTp result obtained from annotation using Trinnotate. The percentage of transcripts is represented on the *y*-axis for each GO class represented on the *x*-axis.

A total of 39,894 genes were assigned to 103,463 gene ontology (GO) terms with the highest GOs in the cellular component (35,057) followed by the biological process (34,247) and molecular functions (34,159) categories. Further, cell and cell parts were predominant categories under the “Cellular Component”, binding and catalytic activity under “Molecular Function”, and cellular process, metabolic process, and regulation of biological process under the “Biological Process” category of gene ontology (Figure 3).

**Figure 4:**
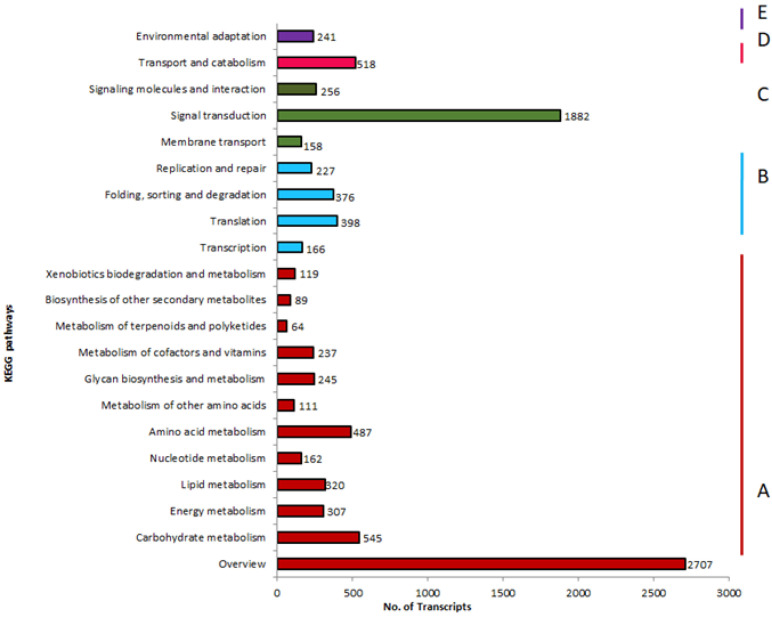
KEGG Pathway classification of assembly A: Metabolism B: Genetic Information Processing C: Environmental Information Processing D: Cellular Processes E: Organismal Systems. No. of transcripts is represented on the *x*-axis under different KEGG pathway classes represented on the *y*-axis.

Moreover, a total of 2707 transcripts were annotated to the KEGG database and classified into 5 broad categories (A–E). Maximum transcripts (69.52%) belonged to the category “C: Environmental Information Processing: Signal transduction” followed by the class “A: Metabolism: Carbohydrate metabolism” with 20.13% transcripts and 19.13% in the class “D: Cellular Processes: Transport and catabolism”. Among the various subcategories under “Metabolism”, 4.39% of transcripts were found to be mapped in xenobiotic biodegradation and metabolism (Figure 4).

**Figure 5:**
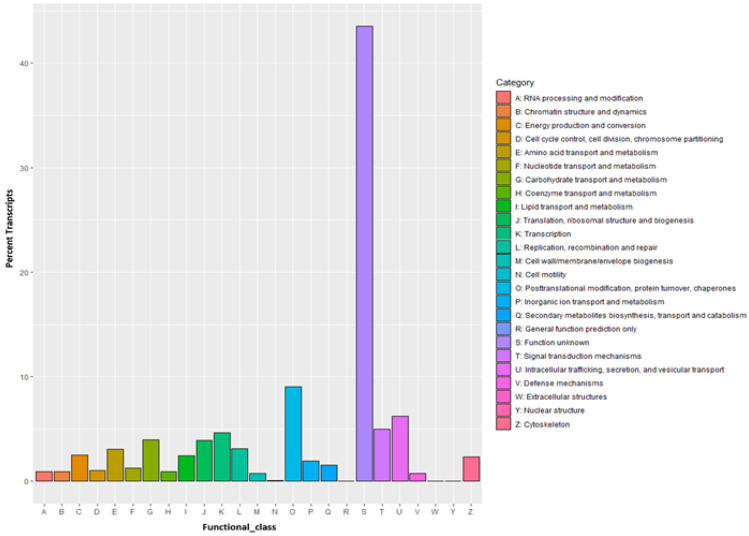
The Cluster of Orthologous Groups (COG) classification of the de novo assembled transcripts. Percentage transcripts are represented on the *y*-axis for each COG functional class on the *x*-axis.

Predicted proteins were also aligned to the Clusters of Orthologous Groups (COG) database to annotate and classify possible functions. As a result, a total of 9116 transcripts were clustered into 24 COG classes. Among them “S: Function unknown” is the biggest cluster with 43.50% transcripts, followed by “O: Posttranslational modification, protein turnover, chaperones” (9.05%), “U: Intracellular trafficking, secretion, and vesicular transport” (6.21%), “T: Signal transduction mechanisms” (4.99%). No hits in “W: Extracellular structures” and “R: General function prediction only” category and few hits in “N: Cell motility” (0.07%), and “Y: Nuclear structure” (0.01%) were found (Figure 5).

### 3.5. Phylogenetic Analysis of C. zastrowi sillemi Cytochrome P450 Sequences

A total of 95 protein-coding genes distributed in 21 families and 47 subfamilies from the four CYP clans namely CYP2, mitochondrial, CYP3, and CYP4 (Table S2) were obtained based on the sequence alignment and subsequent phylogenetic analysis. Due to the fragmented transcripts, we excluded some of the CZS CYP sequences from phylogenetic analysis in CYP2 and mitochondrial clan including Halloween genes viz., DmCYP306A1 Phantom (Phm), DmCYP307A1 spook (spo), disembodied DmCYP302A1 (Dib) and DmCYP314A1 shade (shd) which are known to be conserved across insect species [42].

**Figure 6:**
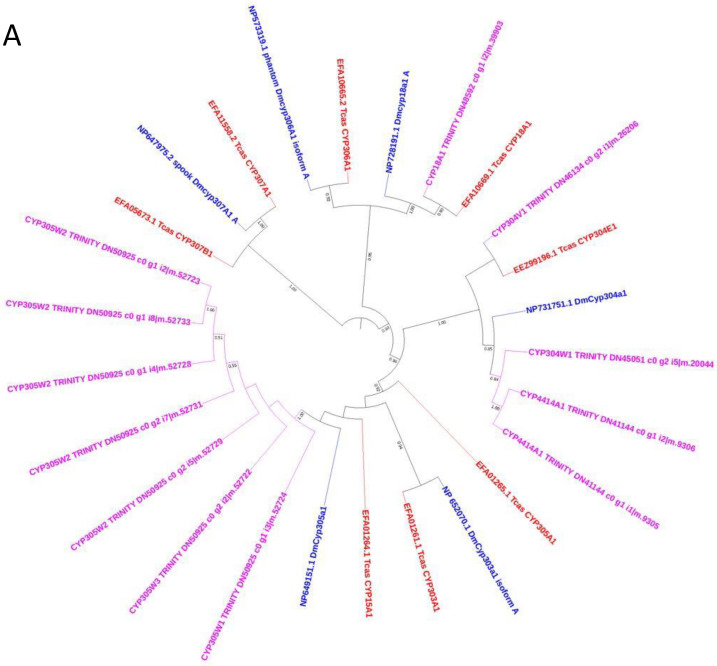

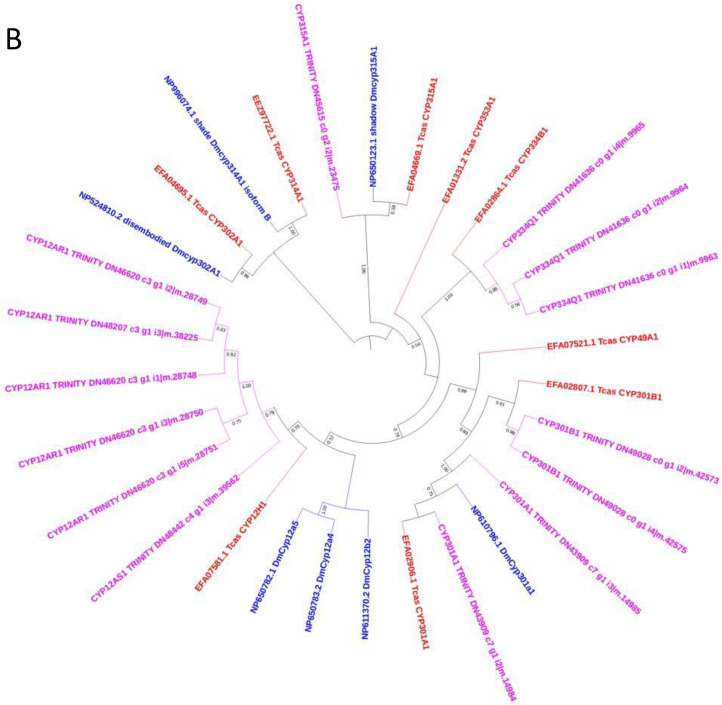

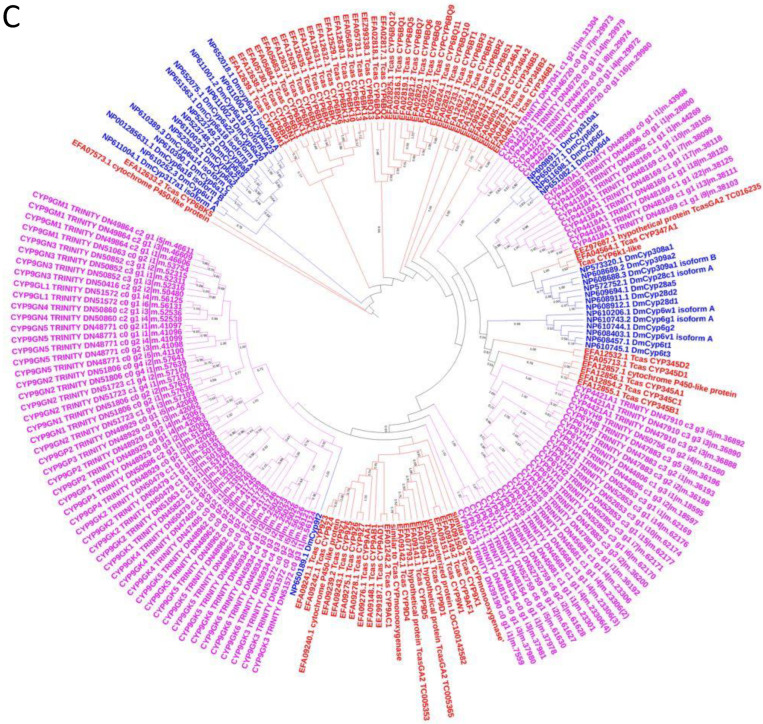

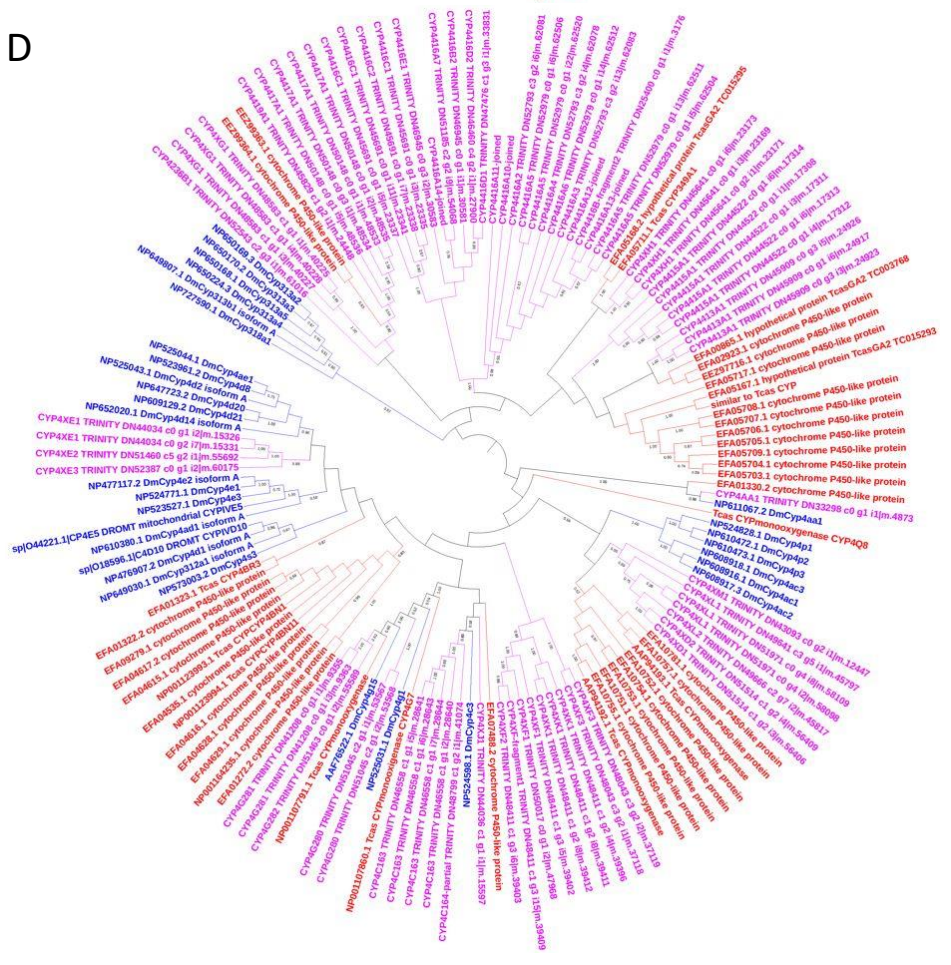
Neighbor-joining consensus trees of the four P450 clans. (**A**). CYP2 clan; (**B**). mitochondrial clan; (**C**). CYP3 clan; and (**D**). CYP4 clan. The phylogenetic tree was generated with 1000 Bootstrap cutoff by MEGA 7 using the amino acid sequences for CYPs from *T. castaneum* and *D. melanogaster*. Node labels are colored pink for *C. zastrowi sillemi*, red for *T. castaneum*, and blue for *D. melanogaster*. Tcas: *T. castaneum*, Dm: *D. melanogaster*.

The CYP2 clan has 4 families (18, 304, 305, 4414) and 4 subfamilies (Table S2, Figure 6A), CYP4414 is the new family reported in this study from the CYP2 clan. Mostly orthologs of CYP genes in this clan are found in other insects. CZS CYP18A1, TcasCYP18A1, and DmCYP18A1 show 1:1:1 orthology. CZS CYP4414A1 and CYP304W1 shares an orthologous relationship with DmCYP304a1 while CZS CYP304V1 is an ortholog of TcasCYP304E1. CYP18 and CYP306 (Phantom (Phm)) are consistent with the earlier findings that they arose from duplication events [43] and are paralogs supported by a 95% bootstrap value in the phylogenetic tree.

The mitochondrial clan has 4 families (12, 301, 315, 334) and 6 subfamilies (Table S2, Figure 6B). The corresponding ortholog of *Drosophila* and *Tribolium* CYP315A1 (shadow (sad) gene) and CYP301A1, which are conserved P450 in insects are also found in CZS. CZS CYP301B1 and CZS CYP334Q1 shares 1:1 orthology with Tcas CYP301B1 and CYP334B1, respectively. CZS CYP12AS1 and CYP12AR1 are orthologs of Tcas CYP12H1 and a paralog of DmCYP12b2, DmCYP12a4, and DmCYP12a5.

The CYP3 clan is composed of 6 families (6, 9, 4418, 4420, 4421, 4422) and 37 genes belonging to 15 subfamilies (6YH, 6YJ, 6YK, 6YL, 9AG, 9GK, 9GL,9GM, 9GN, 9GP, 4418A, 4418B, 4420A, 4421A, 4422A) (Table S2, Figure 6C). CYP (4418, 4420, 4421, and 4422) are the new families reported from the CYP3 clan in the present study. CYP6 includes 4 subfamilies (6YH, 6YJ, 6YK, 6YL), of which CYP6YH has the maximum number of protein-coding genes (9). CYP4421 and CYP6YH subfamilies in the CYP3 clan clusters together with 99% bootstrap support. CZS CYP4422 (2 genes) and CYP6YJ (1 gene) cluster together and has no ortholog or paralog from *D. melanogaster* and *T. castaneum*. CYP6YL and CYP6YK are also unique in CZS. CYP9 with 18 genes from 6 subfamilies (9AG, 9GK, 9GL, 9GM, 9GN, 9GP) is the largest family in the CYP3 clan.

CYP4 has 7 families (4, 4236, 4413, 4415, 4416, 4417, 4419) and 45 genes belonging to 22 subfamilies (4C, 4G, 4AA, 4XD, 4XE, 4XF, 4XG, 4XH, 4XJ, 4XK, 4XL, 4XM, 4236B, 4413A, 4415A, 4416A, 4416B, 4416C, 4416D, 4416E, 4417A, 4419A) (Table S2, Figure 6D). CYP (4236, 4413, 4415, 4416, 4417, and 4419) are the new families reported from the CYP4 clan in the present study. The CZS CYP4416 (A, B, C, D, and E) with 18 genes is unique to CZS and does not share any ortholog or paralog with *D. melanogaster* and *T. castaneum*. CZS CYP4XF (which has 4 genes: XF1, XF2, XF3, and one partial sequence) and CYP4XK (with one gene XK1) are novels, clustered together and form a separate clade from other insects. CZS CYP4C163 and CYP164-partial are the orthologs of DmCYP4C3 supported with a 58% bootstrap value while CZS CYP4G280, CYP4G281, and CYP4G282 are orthologs of DmCYP4g1 and DmCYP4g15. The CZS CYP4XJ1 share sequence homology with the subfamily CYP4C and cluster together in the phylogenetic tree. The CZS CYP4419 and CYP4417 cluster together and are the ortholog of Tcas cytochrome P450-like protein (GenBank: EEZ99363.1 and EEZ99364.1) with 79% bootstrap support. CZS CYP4XG1 and CYP4236B1 are homologous to CYP4417 and CYP4419 and cluster together. CZS CYP4AA1 presents as a single-copy gene as also reported from other insects [42] and also shares a 1:1 orthologous relationship to DmCYP4aa1 and Tcas protein (GenBank: EFA01330.2). CZS CYP4413 clusters with Tcas CYP protein (GenBank: EEZ97716.1, EFA02923.1, EFA00865.1), CYP4XH clusters and shares similarity with Tcas CYP349A1 and TcasGA2. CYP4XE from CZS has 3 genes and is a paralog of TcasCYP4BR3 which is reported to be over-expressed in response to imidacloprid [44].

### 3.6. Differential Gene Expression and Validation by Quantitative Real-Time PCR

Differentially expressed genes (DEGs) were reported between two conditions by EdgeR at FDR < 0.05. The DEGs were further filtered with the following cutoff for down-regulation (logFC ≤ −1.5 and *p* < 0.05) and for up-regulated genes (logFC ≥ 1.5 and *p* < 0.05). Of the differentially expressed genes, all the CYP (10) were selected for validation. Information related to the DE result of CYP genes obtained along with their fold change values and primers used for validation is provided in Table S1.

**Figure 7:**
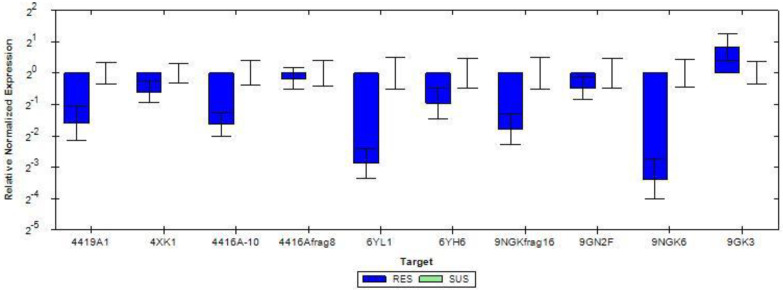
qRT-PCR validation of ten CYP genes in imidacloprid-resistant and lab-reared susceptible strains. Genes considered for validation are represented on the *x*-axis and the Relative Normalized Expression on *y*-axis. RES: resistance, SUS: susceptible.

qRT-PCR was conducted to verify the expression level of differentially expressed CYP genes. The results highly correlated with the transcriptome data. CYP4419A1 (−1.62 fold), CYP4416A10 (−3.9), CYP4416Afrag8 (−3.05), CYP4XK1 (−1.93), CYP6YL1 (−5.66), CYP6YH6 (−2.05), CYP9GKfragment16 (−3.93), CYP9GN2 (−1.51) and CYP9GK6 (−8.23) were downregulated and CYP9GK3 (3.31) was up-regulated after imidacloprid treatment (Figure 7).

## 4. Discussion

In general, under field conditions, natural enemies succumb to a different group of insecticides either through direct exposure to spray or by consuming the poisoned prey. In many cases, the application of chemical pesticides is lethal to predatory insects and further leads to pest resurgence and a secondary outbreak of minor pests. Imidacloprid, a systemic neonicotinoid is widely used for the control of insect pests, especially sucking pests. The indiscriminate application of imidacloprid in the field harms the non-target arthropods such as pollinating bees and other natural enemies which are important for our natural and agricultural ecosystems [45]. Imidacloprid is reported to adversely affect the olfactory, nervous, energy metabolism, and detoxification system of the predators [46], reducing their predatory potential [47] and even survival of the natural enemies [48]. Nevertheless, natural enemies can develop resistance when they are constantly exposed to chemical insecticides [49].

When insecticides are one of the components of IPM, widely adopted by a majority of the farmers, integrating the insecticide-resistant predators will enable us to suppress the pests sustainably and would delay the development of insecticide resistance in the insect pests. Among the natural enemies, the populations of green lacewing have revealed a significant level of resistance development against different insecticides [24,50]. The insecticide-resistant predators could be useful to target the pests under insecticide-stressed crop ecosystems thus, reducing the pesticide load on the crops and the cost of protection. Furthermore, the use of such insecticide-resistant predators will help in reducing pesticide resistance and the resurgence of insect pests.

Keeping the above in view, we have developed an imidacloprid-resistant CZS strain by exposing the predator to insecticide continuously in the laboratory. Such imidacloprid-resistant strain was subjected to transcriptome sequencing and compared with the susceptible strain. We have generated a transcriptome assembly with an N50 of 1391 and 95% of the arthropod′s complete BUSCO groups. Further, 85% of the reads mapped back to the assembly concordantly, signifying our reads sequenced were in proper pair. The remaining unassembled reads might be those that belonged to the lowly expressed genes with insufficient coverage.

The distribution of transcripts annotated by Gene Ontology is similar to the transcriptome studies carried out in other neuropteran *C. pallens* [51] where cell, cell part, cellular process, and metabolic process were among the predominant GO classes. In the KEGG pathway classification, the “Signal transduction” class was the most dominant, signifying their role in the regulation of gene expression linked to apoptosis, growth, motility, differentiation, homeostasis, metabolism, and multidrug efflux. The category “Function unknown” is the most enriched in COG classification for the assembly, indicating a majority of transcripts to be either non-coding or specific to the CZS. The next predominant COG class includes “Posttranslational modification, protein turnover, chaperones” and “Intracellular trafficking, secretion, and vesicular transport” which indicates the activity of biologically important proteins under the insecticide treatment to regulate function, activity, localization, and substrate specificity.

Our study also provides a large CYP sequence information that is not yet reported for *C. zastrowi sillemi*. Table S3 provides a comparison of the CYP genes in the 22 insects including *C. zastrowi sillemi*. In general, more CYP genes are reported in polyphagous insects such as *Tetranychus urticae* (78) and *Helicoverpa armigera* with 112 CYP genes. *Chrysoperla zastrowi sillemi* is a generalist predator and has more number (95) of CYP genes than *Acrythosiphum pisum* (64), *Myzus persicae* (65), and comparable with the parasitoid wasp *Nasonia vitripennis* (90), which is also used as a biological control agent.

Classification and phylogeny are frequently used in discerning the function and evolution of CYP genes. One of the most common ways is to find out the orthologs and paralogs from the related species [38,52,53]. Our phylogenetic study revealed that CYP2 and mitochondrial clans in CZS are less divergent and mostly share an orthologous relationship with the CYP genes from *D. melanogaster* and indicating they have evolved from a common ancestor and later differentiated due to speciation. The ortholog of CYP18A1, CYP315A1, CYP301A1, and CYP301B1 are found in CZS. From the earlier studies in insects, we know that most of the genes from CYP2 and mitochondrial clans including the Halloween family are involved in ecdysteroid metabolic pathways. CYP18A1, which is an ecdysteroidogenic P450 with conserved 26-hydroxylase activity in insects, is involved in ecdysteroid catabolism and thus, essential for proper insect development [54]. CYP315A1 is a member of the Halloween gene family and catalyzes the hydroxylation reactions in the ecdysteroidogenic pathway controlling the biosynthesis of 20-hydroxyecdysone, the molting hormone of insects [55]. Furthermore, RNAi experiments in *Drosophila* with CYP301A1 produced adults with distinct morphological disruption of cuticle suggesting its crucial role in cuticle formation in adults [56]. Therefore, CYP18A1, CYP315A1, and CYP301A1 may be involved in ecdysteroid metabolic pathways however, their specific function requires further validation in CZS. CYP301B1 is reported in providing resistance to plant-derived insecticide, beta-asarone in *Nilaparvatha lugens* [57]. CYP12 family includes genes associated with insecticide resistance in the house fly *Musca domestica* [58], *D. melanogaster* [59], and *T. castaneum* [38] which contain a single CYP12H1 gene in the genome. However, two genes from the insecticide-resistant CYP12 family viz., CYP12A1 and CYP12AS1 have been found in the transcriptome of CZS. The role of the CYP301B1 and CYP12 gene family can be studied further for the inheritance pattern of insecticide resistance in CZS. There are three genes, W1, W2, and W3 for the CYP305 family which is known for the detoxification of plant allelochemicals in *D. melanogaster* [60]. Studies related to their function in CZS can provide major insight into the tritrophic interaction.

Species-specific gene expansion is seen in the CYP3 and CYP4 clans as is also evident from several studies reporting the occurrence of CYPome “blooms” [61] where some families have many genes while many have only a few; a similar pattern has been reported in other insects [43]. Most of the CYP genes in CZS belong to the CYP3 and CYP4 clans similar to that reported from the transcriptome of *Chrysopa pallens* [51]. CYP4 contains the highest number of genes (45) which accounted for 47% of total CYP genes from 7 families (33%) and 22 subfamilies (47%) followed by the CYP3 clan which contains a total of 37 genes (39%) from 15 subfamilies (32%) in 6 different families (28.6%). The CYP3 and CYP4 clan are the most dynamic as well as predominant in the insect species where expansion or loss of some families are observed for the adaptation and xenobiotic detoxification [52,53]. CYP6 and CYP9 families showed maximum diversity with CYP6YH as the largest subfamily and CYP9 as the largest family. Genes belonging to CYP6 and CYP9 families have been involved in the detoxification process conferring insecticide resistance [62]. CYP6 family genes are shown to have a role in the detoxification of insecticides as well as of allelochemicals by constitutive and or induced over-expression in the resistant strains [18]. They have also been reported to be over-expressed in the presence of imidacloprid in *M. persicae* [63], *Leptinotarsa decemlineata* [52], and *N. lugens* [64]. From the phylogenetic analysis, CZS CYP6YH appears to be a paralog of DmCYP6g1 (RefSeq: NP610743.2) (Figure 6C), a gene with an established association with imidacloprid resistance [65,66] and to the TcasCYP345A1 (GenBank: EFA12856.1) which is also found to be involved in imidacloprid resistance [44]. The downregulation of CYP6YH6 found in imidacloprid-resistant CZS indicates the possible involvement of this gene in the resistance mechanism. Members of the CYP9 family are also reported to be associated with insecticide resistance in *Spodoptera exigua* [67] and mosquitoes [68]. Our analysis shows that CYP9 family genes have considerably expanded and formed *Chrysoperla*-specific separate clades in the phylogenetic tree (Figure 6C).

The wide diversity in the CYP4 clan is attributed to insecticide resistance, for example in *Triatoma infestans* for deltamethrin resistance [69]. CYP4 clan in *Chrysoperla* is also showing diversity with 6 new CYP families and gene expansion in the family CYP4 (with 22 genes from 12 different subfamilies altogether) and subfamily CYP4416 (with 18 genes). CYP4G family is known to catalyze the synthesis of cuticular hydrocarbons that serve multiple functions from desiccation resistance to chemical communication [70]. CYP4g1 is involved in lipid metabolism [71] and CYP4g15 is expressed in the brain and central nervous system [72] in *D. melanogaster* and is among the conserved genes across all insects. In the present study, we report three genes CYP4G280, CYP4G281, and CYP4G282 from the CYP4G family, the role of which can be explored further. Two genes from the CYP4416 family (CYP4416A10 and CYP4416Afrag8) and four genes from the CYP9 family (CYP9GK-fragment16, CYP9GN2, CYP9GK6, CYP9GK3) are differentially expressed after imidacloprid treatment suggesting the probable link between the expansion of genes and the detoxification mechanism in CZS.

## 5. Conclusions

Our study provides sequence information of the 95 CYP genes from the transcriptome of *C. zastrowi sillemi* and serves as a preliminary resource to investigate the functions of P450 genes. Phylogenetic analysis of the CYPs along with those from other insects helps in understanding the evolutionary diversity of CYPs in *C. zastrowi sillemi.* Differential expression study provides information on relative changes in expression of CYPs in response to imidacloprid which helps in understanding the detoxification mechanism in *C. zastrowi sillemi*. In addition, the information provided in the current study can also be exploited to study cross-resistance to other insecticides and prey-derived chemicals, allowing for a wide application of the predators with minimum effect on their fitness.

## Figures and Tables

**Table 1:** Dose-mortality of different field populations of CZS against imidacloprid 17.8 SL.

Sl. No.	Population	LC_50_ (mL/Liter)	Slope ± SE	Fiducial Limits		LC_90_ (mL/Liter)	Fiducial Limits		X^2^	Df	RR
				Lower	Upper		Lower	Upper			
1.	Coimbatore	0.33	1.08 ± 0.28	0.116	0.618	5.99	2.197	131.74	8.8	7	3.60
2.	Ludhiana	0.17	0.98 ± 0.30	0.027	0.347	3.47	1.384	80.072	7.52	7	1.90
3.	Sirsa	0.45	1.18 ± 0.29	0.232	0.815	5.53	2.310	51.35	7.34	7	5.0
4.	Delhi	0.47	1.45 ± 0.31	0.275	0.758	3.53	1.766	16.399	0.225	7	5.20
5.	Darwad	0.41	1.39 ± 0.31	0.230	0.678	3.46	1.690	17.823	6.93	7	4.50
6.	Guntur	0.36	1.29 ± 0.31	0.180	0.603	3.53	1.655	22.23	12.49	7	4.0
7.	Sriganganagar	1.08	1.67 ± 0.32	0.709	1.852	6.26	3.154	24.797	15.29	7	12.0
8.	Anand	0.33	1.24± 0.31	0.156	0.567	3.55	1.633	25.12	9.56	7	3.60
9.	Susceptible	0.09	1.09 ± 0.35	0.004	0.197	1.34	0.643	16.510	6.54	7	

**Table 2:** Dose mortality of imidacloprid resistant and susceptible strains of CZS against Imidacloprid 17.8 SL.

Sl. No.	Strain	LC_50_ (mL/Liter)	Slope ± SE	Fiducial Limits		LC_90_ (mL/Liter)	Fiducial Limits		X^2^	Df	RR
				Lower	Upper		Lower	Upper			
1.	Resistant (Sriganganagar)	2.24	1.10± 0.15	1.473	3.921	32.58	14.101	132.713	8.8	7	56
2.	Susceptible	0.04	1.0 ± 0.15	0.016	0.065	0.713	0.419	1.622	1.62	7	

## Data Availability

The raw sequence data has been submitted to the NCBI Sequence Read Archive (SRA) with accession number SRR3820550 SRR3820549 SRR3820548 SRR3820547. This Transcriptome Shotgun Assembly project has been deposited at DDBJ/ENA/GenBank under the accession GKCC00000000. The version described in this paper is the first version, GKCC01000000. CYP sequences have been submitted to GenBank under accession numbers ON646299-ON646458.

## Supplementary Materials

The following supporting information can be downloaded at: https://www.mdpi.com/article/10.3390/insects13100900/s1, Table S1: Differentially expressed CYP genes, primers used for real-time validation experiments, and fold changes as obtained from EdgeR and qRT-PCR in imidacloprid-resistant populations; Table S2: Total number of genes from different families from the four CYP P450 clans identified in CZS; Table S3: A comparison of CYP gene numbers in 22 insects including CZS; File S1: CZS 160 CYP protein sequences along with their GenBank accession number.
