# Supplementary material for: Comparative Transcriptome Analysis to Reveal Differentially Expressed Cytochrome P450 in Response to Imidacloprid in the Aphid Lion, Chrysoperla zastrowi sillemi (Esben-Petersen)
Source: Insects. 2022 Oct 3;13(10):900. doi: 10.3390/insects13100900 (PMC9604014; doi:10.3390/insects13100900)
Supplement: Supplementary file 1 [file insects-13-00900-s001.zip › insects-1838503-supplementary.pdf]

Comparative Transcriptome Analysis to Reveal Differentially Expressed Cytochrome P450 in Response to Imidacloprid in Aphid Lion, *Chrysoperla zastrowi sillemi* (Esben-Petersen)

Jyoti Pathak[1], Gandhi Gracy Ramasamy[1], Aditi Agrawal[1], Subhi Srivastava[1], Bhusangar Raghavendra Basavaarya[1], Mohan Muthugounder[1], Venugopal Kundalagurki Muniyappa[1], Pratheepa Maria[1], Anil Rai[2], Thiruvengadam Venkatesan[1] *

[1] Division of Genomic Resources, ICAR-National Bureau of Agricultural Insect Resources, P.Bag No: 2491, H.A. Farm Post, Bellary Road, Hebbal, Bangalore-24, India

[2] Centre for Agricultural Bioinformatics, Indian Agricultural statistical Research Institute, Pusa, New Delhi-110012, India

*Correspondence: tvenkat12@gmail.com

Table S1 Differentially expressed CYP genes, primers used for real-time validation experiments and fold changes as obtained from EdgeR and qRT-PCR in imidacloprid-resistant populations.

CYP_Gene	Protein_ID	Fold Change in Transcritome	Fold Change in qRT-PCR	Forward and Reverse Primer Used for qRT-PCR	Primer_ Length
CYP4419A1	TRINITY_DN45829_c1_g2_i1|m.24448	-1.75351	-1.62	TGCTAATTCACGACCGGATTC –F<br>TGCCCTGTTTACTCATAGCTTC -R	21<br>22
CYP4XK1	TRINITY_DN48411_c1_g2_i5|m.39401	-3.18927	-1.93	TAATACCAGGACCCCAAGGAC -F<br>CCAATGTTCACCAGCACTTG -R	21<br>20
CYP4416A10	TRINITY_DN52793_c3_g3_i2|m.62079	-1.87088	-3.9	AGTTGTTGGTGACACAAATG -F<br>CCAATATTCTGGATTTCGATGG -R	20<br>22
CYP4416A-fragment8	TRINITY_DN52793_c3_g2_i3|m.62076	-2.75892	-3.05	GATAGGCCACACCTCAAGAAG -F<br>AGGAGGAGGTGAAGAAGAGG -R	21<br>22
CYP6YL1	TRINITY_DN52759_c6_g1_i5|m.61630	-12.6133	-5.66	CACAATGAGCTATGAAGCTGTG -F<br>CGTGGTGAAGCGATAACAATG -R	22<br>21
CYP6YH6	TRINITY_DN47883_c2_g1_i1|m.36192	-2.15479	-2.05	AGGTGATTTGGGTACTG -F<br>CACCGTTTCGTACAATG -R	17<br>17
CYP9GK-fragment16	TRINITY_DN51061_c2_g1_i5|m.53627	-3.09926	-3.93	ATTTGCCGTAGTGGAAGAGG -F<br>GAAGCAACAGACCATTTTCG -R	20<br>20
CYP9GN2	TRINITY_DN51723_c1_g4_i1|m.57100	-1.58947	-1.51	GGAATTTGAACACCCATACCAG -F<br>GCTGCTTACGAATTAGCTGTC -R	22<br>21
CYP9GK6	TRINITY_DN45934_c4_g3_i2|m.25022	-7.41136	-8.23	GTGACTTCTGCATTGGGATTTC -F<br>TGCTACTGAAGGGAAAGTTGG -R	22<br>21
CYP9GK3	TRINITY_DN51572_c0_g2_i2|m.56121	1.643084	3.31	AAATGGCGTGACATGAGAGC -F<br>TTACGATCGCGCAACGAATC -R	20<br>20
28S				TGTTCATGGGCCCGTATTGATG -F<br>ATGCAGCGGAAAGAGCTTCTAC -R	22<br>22

Table S2 Total number of genes from different families from the four CYP P450 clans identified in CZS.

CYP Clan	Families	Subfamilies	No. of Genes
**CYP2 clan**	18, 304, 305, 4414	18A	1
		304V	1
		304W	1
		305W	3
		4414A	1
**Total in CYP2 clan**	**4**	**5**	**7**
**CYP3 clan**	6, 9, 4418, 4420, 4421, 4422	6YH	9
		6YJ	1
		6YK	1
		6YL	1
		9AG	1
		9GK	6
		9GL	1
		9GM	1
		9GN	5
		9GP	4
		4418A,	1
		4418B	2
		4420A	1
		4421A	1

		4422A	2
**Total in CYP3 clan**	**6**	**15**	**37**
**CYP4 clan**	4, 4236, 4413, 4415, 4416, 4417, 4419	4C	2
		4G	3
		4AA	1
		4XD	2
		4XE	3
		4XF	4
		4XG	1
		4XH	1
		4XJ	1
		4XK	1
		4XL	2
		4XM	1
		4236B	1
		4413A	1
		4415A	1
		4416A	11
		4416B	2
		4416C	2
		4416D	2
		4416E	1
		4417A	1

		4419A	1
**Total in CYP4 clan**	**7**	**22**	**45**
**Mitochondrial clan**	12, 301, 315, 334	12AR	1
		12AS	1
		301A	1
		301B	1
		315A	1
		334Q	1
**Total in Mitochondrial clan**	**4**	**6**	**6**
**Total**	**21 families**	**48 Subfamilies**	**95 Genes**

Table S3 A comparison of CYP gene numbers in 22 insects including CZS.

**CYP Clan**	**CYP2**	**CYP3**	**CYP4**	**Mitochondrial CYP**	**Total**
Chrysoperla zastrowii silemii	7	37	45	6	95
Drosophila melanogaster	7	36	32	12	87
Anopheles gambiae	10	38	43	9	100
Tribolium castaneum	8	68	43	9	128
Bombyx mori	7	29	35	11	82
Helicoverpa armigera	8	45	49	10	112
Apis mellifera	8	31	4	6	49
Nasonia vitripennis	6	46	31	7	90
Tetranychus urticae	38	9	26	5	78
Metaseiulus occidentalis	16	23	19	5	63
Rhynchophorus ferrugineus	3	43	23	8	77
Acyrthosiphon pisum	10	23	23	8	64
Myzus persicae	10	25	23	7	65
Agasicles hydrophila	7	39	30	6	82
Leptinotarsa decemlineata	4	39	20	11	74
Culex quinquefasciatus	16	89	82	12	204
Aedes aegypti	11	84	59	10	164
Cnaphalocrocis medinalis	5	16	9	6	36
Trialeurodes Vaporariorum	5	49	15	9	78
Pogonomyrmex barbatus	8	61	40	6	115
Danaus plexippus	8	36	30	12	86
Plutella xylostella	10	26	36	13	85

*Data are derived from Feyereisen, (2012), Antony *et al.*, (2019), Zhang et al., (2018), Dermauw *et al.*, (2020) and current study.

File S1. CZS 160 CYP protein sequences along with their GenBank accession number.

>ON646299 CYP4416Bfragment2 [369 AA] TRINITY_DN25400_c0_g1_i1|m.3176
MFLKYIESNIVQILLTSVFVLIVYKFSNYIRIFYYVSKLPGPPAYPIIGNALHFIGKNKDSSQKFL
KIFEDYNSFFRLWIGLIPGVCISNPDFIQKLLNNKNGLEKSFLMKYFGKEAMGDSIILSDVVKW
KQNRGIVARGFTPLILKSYFKIFVEKSLMLVEKLDEKLNDGESFDIYEYLSKTTLEAVCGSTM
GVNIDISNSYYSALESPFHLGMHRIYHFWEIPNCTYRWTDNYKKFNESIKTVREFTHNVFLQK
KKYYNSKNNENVDEIKSNLKNEFKQPLLEYLIDFAAVNLDFTEGDIEIEANLAIMAGYETLST
TLSYVILNLALHKYIQNKVCEELFEVLGNDLDRTIAVEDLSKLKYMEMVI

>ON646300 CYP4AA1 [379 AA] TRINITY_DN33298_c0_g1_i1|m.4873
NFLGEGLITNTGAKWKQHRKLIQPIFTNNILESFIDTFSDSAQSLLTKLSAHSGQDINITSYINNC
VLDILNEAVLGVPINNNCAEDIGESPFRKGEIVAPYRLVRPWLLFNWIYELTEVARRELEQKQ
NLNNFTKKMIEYNKNIDKTKCSGRKSLLAMFQEIAERHPNFTEHDIVQEIRTFMLAGQDSVGS
ALAFCLYSLAKHQEHQDIVIKELDTIFKDSNRSPNARDLDEMCYLEQCIKETLRLYPSVPLVA
RKISEDIKLNDKYTIPKNCTVFISPYFTQRLPDIYPDPDNFIPSRFSREEILKRHPYSFIPFSAGPR
NCIGYKFSIMEMKTVISTILRHYRITLSPGKENLTLSWRITIRARGGIWLRLIPRTS

>ON646301 CYP9AG9 [508 AA] TRINITY_DN39190_c0_g1_i1|m.7559
MELTTWLLIGAVLYAIWAFLNRRLQYFESLNLPYVPGVPFFGSMASAVFRVCHISDTVKNIYR
YNPEAKYIGAFDFMRPVIVLRDLDVIKNVTIKNFDNFPDHQAFIDDSVDPLFGGGLFNIAGER
WKEARALLSPAFTSSKMKGMFELMVVCGKNFVDYLSKLPEKERKAIATKDMFGKLTNDVIG
TCAFGININSLENPKNDFFILGKEATTFDGIMSLKFFIARSFPNVMKWFNIKFVSDRVERFFTSI
VSTTIATRDEQGISRPDMIQLMMDARGKEGKNFKLDITSMTAQAFIFFFGGFDTTSTQMCIIAH
ELAINPDIQKRLQDEIDDVLERSKGKLTYEALNSMLYLDAIFNESMRFHPQAGFLDRLCVKAF
ELPPALPGLKPFVVQPGMNVWIPAVAIHRDPEYHDEPDTFNPDRYYQKKVTINDTQNLGFGI
GPRSCIGNRFAIMETKLLFFFLLSKFNLVPNDKTCSPLVYSKKTFSLIPPGGYSLAIEPRTDASA
YA

>ON646302 CYP4414A1 [499 AA] TRINITY_DN41144_c0_g1_i1|m.9305
KMISPLLFGILSTLFIYDIFYFINGRPKNFPPGPIRIPFFGSYLFLIFKTLKDVPIELAEWSKKYKS
NVTSLFLGPYKNIILHDYQTIQILTRNEFDGRPDGFIIKLRAFGKRLGIFFTDGDFWLEQRRFAL
RHMRDFGFGRRSEKLENIIEFEMKEMIKHIENNHYMFKAPHFFAPGFVNLLVSILTGATLDQN
QRNYLEEIGFHSLLFQKNASPLGGFIGIFPWVRFFAPNVSGYHGSKNGNDKLIEFVQDLIENRK
KLRHKEETEGFIDVYLQELENREKANKSSFTPEQLILTCVDFLLPTSSCVEMSLSLLIQRLIHHP
DVQDKMFDEIKSVVGTGRLPTLNDRPSLLYCEATLREGMRIDTPINLGVGRETTTNINVQGY
DIPKGTLVFANLWQMHHDEILWKDPFAFRPERFLTDDGKRLCKDMTLPFGLGRRVCAGETF
ARNGMFMFLVGLVQNFKFTVPPGALLPDLSDRANSALLTNCQEFIVQCEPRI

>ON646303 CYP4G281 [560 AA] TRINITY_DN41208_c0_g1_i1|m.9355
MSAAGPEMVTGTLATASATGAFSATSVFFTLLVPALVLYYIYFRVTRRHMIELADKIPGPTGF
PLIGNALELLGSSDTIFKNVYKRSFEFDQVIKLWIGPKLVVFLIDPRDVEVILSSHVYIDKSPEY
RFFQPWLGNGLLISTGQKWRAHRKLIAPTFHLNVLKSFIDLFNANSNAVVEKLRKEGNREFDI
HDYMSETTVEILLETAMGVSKTTQDKSGFEYAMAVMKMCDILHLRHTRVWLRPDWLFNLT
KHGKEQVKLLDIIHGLTKKVVARKKEDYKSGKRNFVEVSKEQAKSTTVVEGLSFGQSSGLK
DDLDVDDNDVGEKKRQAFLDLLVEASQNGVVLTDEEMKEQVDTIMFEGHDTTAAGSSFFLS
MMGCHPDIQEKVIQEIDEIFGDSDRPATFQDTLEMKYLERCLMETLRMYPPVPIIAREVKTDL
KLASGDYTIPAGCTVVVATFKLHRQPHIYPNPDVFNPDNFLPEKTANRHYYAFVPFSAGPRSC
VGRKYAMLKLKILLSTILRNYRVKSTVKEEDFRLQADIILKRAEGFKVKLEPRKRAGGVKA

>ON646304 CYP334Q1 [570 AA] TRINITY_DN41636_c0_g1_i1|m.9963

MVRPLLRLRRLPFYLQIRNQETSTKIKSIESVPPATTASLHSTDVGTVLGESTIQIKTPSLNEIRV
KEVDAATQTDSSLKLIQNFDEVPGPRVLKNVANILRYIPSLSTHVTATTIQYILSTGSQLVGGA
DSRFIKWLFDEYGPVVRLEAPLGGNIVILSRPEHVHAVFKNEGPYPIRSSLDCIEKYRLQYRKY
KQAGPYIMHGPEWIQLRSKIQTPLESMLDIHFQNLQSSCDRFVKIIKKIRNQQEETPAEFHSEIY
KWSMDCLTIVSFNKELGFLDAGGLRPTSEAMALFNALKDATAAIHKCEGGLKLWKFFVTRD
WKLLLKQLDTIDNIVNKYIGSIQLAIEDKDSKLSIGPNSLVEALLVKEQLQPEDILTVLVDLMII
GVNTTSHAMAFLLYHLARNPKVQKKLYNEIRNANLYEINSYEKLPYLQACIKESLRLKPPMP
VLSRILQQDVIIHNYRIPKDTFVLMATHLASLRDENFENPTKYDPERWIDDTDSGTQSLASIPF
GFGPKSCLGQKLAMLQLSMMTAKIIKEFNIQYNYGDITAGTKILSNPNRPLKFRFIERN

>ON646305 CYP334Q1 [365 AA] TRINITY_DN41636_c0_g1_i4|m.9965
MIVNFGSVYQAYFLMEFEFYFLNLQSSCDRFVKIIKKIRNQQEETPAEFHSEIYKWSMDCLTIV
SFNKELGFLDAGGLRPTSEAMALFNALKDATAAIHKCEGGLKLWKFFVTRDWKLLLKQLDT
IDNIVNKYIGSIQLAIEDKDSKLSIGPNSLVEALLVKEQLQPEDILTVLVDLMIIGVNTTSHAMA
FLLYHLARNPKVQKKLYNEIRNANLYEINSYEKLPYLQACIKESLRLKPPMPVLSRILQQDVII
HNYRIPKDTFVLMATHLASLRDENFENPTKYDPERWIDDTDSGTQSLASIPFGFGPKSCLGQK
LAMLQLSMMTAKIIKEFNIQYNYGDITAGTKILSNPNRPLKFRFIERN

>ON646306 CYP4XM1 [385 AA] TRINITY_DN43093_c0_g2_i1|m.12447
SAGDYWRIRRKMLTPAFHFTILKQFANIFEENSKLAVQNLSKECEKLSTNVAEYINDYALHTLCDT
TLGCKFESTSAEFSNYKKALHRFGEIAVYRLSRPWVFPDFLFNLSYLKYKTIQTIKTLRSFTSKIITS
RLETWDPNIVDNTTIETTSGRRLYSMLDLLILHMKTNGGIDYKGIQEEVDTFVFEGHDTTSVAIVN
MLMLLANHTEVQDNIVRELYSVVGEDISKISYNDLQSLKYLEMCIKETLRLYPSVPFIARTLTEPLE
LKDGVVLPKGLLCNVHIYDLHHDAEYWPEPEKFIPERFTEENCAKRNPFCYVPFSAGPRNCIGQKF
AILQMKSIISNILFNYILEPIDTPESITLLCDLVIRTEKPVRVKFRKRVRNA

>ON646307 CYP301A1 [490 AA] TRINITY_DN43909_c7_g1_i2|m.14984
MKFNSKQLIFDCSKLLRTHQQYRLKSYVAQTSEIPATVKPYKDIPGPRPIPLLGNTWRLLPIIGQYQI
ADFANVCDILHKEYGKICKLSGLVGRPDLLFVYDANEIEKVYRHEGPTPYRPSMPCLVYYKSVVR
KDFFGDLPGVVGVHGEPWREFRTRVQKPVLQLHTVRKYIEPIEVVTNDFIQRMNEMKDENDEMP
SDFDNEIHKWALECIGRVALDVRLGCLDPNLSPDSETQKIIDAAKYALRNVAILELKMPFWRYIPS
SLWTKYVKNMNYFIEICMKHIDAAMQRLKQKNVTSDKDLSLIERILTSEKDPKIAYVLALDLILVG
IDTISMAVCSILYQLATRPEEQEKIHQELLKALPDPNTPLTSKHLEQVPYLKAFIKEVFRVYSTVIGN
GRTLQQDLEICGYHVPKGIQCVFPTIITGNMEDYVTNASTFMPERWLSKHSENSSNNTIHPFASLPY
GHGARMCLGRRFADLEMQILLAKVSF

>ON646308 CYP301A1 [523 AA] TRINITY_DN43909_c7_g1_i3|m.14985
MKFNSKQLIFDCSKLLRTHQQYRLKSYVAQTSEIPATVKPYKDIPGPRPIPLLGNTWRLLPIIGQYQI
ADFANVCDILHKEYGKICKLSGLVGRPDLLFVYDANEIEKVYRHEGPTPYRPSMPCLVYYKSVVR
KDFFGDLPGVVGVHGEPWREFRTRVQKPVLQLHTVRKYIEPIEVVTNDFIQRMNEMKDENDEMP
SDFDNEIHKWALECIGRVALDVRLGCLDPNLSPDSETQKIIDAAKYALRNVAILELKMPFWRYIPS
SLWTKYVKNMNYFIEICMKHIDAAMQRLKQKNVTSDKDLSLIERILTSEKDPKIAYVLALDLILVG
IDTISMAVCSILYQLATRPEEQEKIHQELLKALPDPNTPLTSKHLEQVPYLKAFIKEVFRVYSTVIGN
GRTLQQDLEICGYHVPKGIQCVFPTIITGNMEDYVTNASTFMPERWLSKHSENSSNNTIHPFASLPY
GHGARMCLGRRFADLEMQILLAKLIRSYKLEYHHEPLKYAVSFMYAPEGELKFKLTKRE

>ON646309 CYP4XE1 [513 AA] TRINITY_DN44034_c0_g1_i2|m.15326
MLWLIIQICVTLFIGLLVVLYLQQLQKSKKILLPSPPLIPLLGHALEFGSTAQDIHNNILRLVSTYGK
LFTIWVGPFNQYCIISDSDVTNKILSSTKLIEKSGDYDLIKPWLGDGLLISKGTKWFKRRKMLTPAF
HFTILEQSINTFTEASDRFIENLKKHVNGDTFDVYPYVTLLGLDIICKTAMGIDLNVQTQPDSEYVT
AVRDMCRIIFERVFSAVQQSDFIFYNLTANGKTFKNGLSILHSMTNKVIEERRQLLINDIKNNEVKD
NGPEKTKDDISFGEKKEKMVFMDVLLKSTVDGKPLTNSDIQEEVDTFMFEGHDTVSSGISFTLYLL
ANHPEIQTRAFEEVRDICGDNLNDQPSLSDLNKMPYLEAIIKESMRLYPPVPVILRNLSEDLKLDNC
VLPKHTRATIVIYCIHRNPEYFSDPESFKPERFLEETQNKNYFSYVPFSAGSRNCIGQKFAMYEMKS
TLSKVLRHYELCSAGPEFDLKLAMAVILKSLNGVNIKVRPRIYK

>ON646310 CYP4XE1 [476 AA] TRINITY_DN44034_c0_g2_i7|m.15331
MLWLIIQICVTLFIGLLVVLYLQQLQKSKKILLPSPPLIPLLGHALEFGSTAQDIHNNILRLVSTYGK
LFTIWVGPFNQYCIISDSDVTNKILSSTKLIEKSGDYDLIKPWLGDGLLISKGTKWFKRRKMLTPAF
HFTILEQSINTFTEASDRFIENLKKHVNGDTFDVYPYVTLLGLDIICKTAMGIDLNVQTQPDSEYVT
AVRDMCRIIFERVFSAVQQSDFIFYNFTANGKTFKNGLSILHSMTNKVIEERRQLLINDIKNNEVKD
NGPEKTKDDISFGEKKEKMVFMDVLLKSTVDGKPLTNSDIQEEVDTFMFEGHDTVSSGISFTLYLL
ANHPEIQTRAFEEVRDICGDNLNDQPSLSDLNKMPYLEAIIKESMRLYPPVPVILRNLSEDLKLDNC
VLPKHTRATIVIYCIHRNPEYFSDPESFKPERFLEETQNKNYFSYVPFSAGSRNCIGLYNLSIVYSFY
YSIQNR

>ON646311 CYP4XJ1[493 AA] TRINITY_DN44036_c1_g1_i1|m.15597
MILVYALITFYIFWKYIKFYKLQKIFVNIPSPKGYPILGNYLDFINSSDSEIFKLTCDRCSTYYPRLW
AVWIWMKPALILHKAEHIEAILKGVIPTKKSRVYKYVEPWLGQSLLTSAGMHWKTHRKLLTPAF
HFTILKEFQSVISEKADILVDRLKTAMENNQEIELLPYINQCTLDVICETSMGVSINAQSDSPPVYYE
AITNFTELAIKRQFSMFGKYEFLWNLTSQGRRQNECLKILHDFADAVIQERKHKLQQNITDENNNE
EDNRKLKPFLDIMLESDEPLTDKEIRDEVSTFLFAGHDTSSTNINWTLFLLGNYPHIQDRVYEELNS
IFNGSKRHATNDDLIEMKYLEMCIKESLRMYPSAPRISRVITEDTNIDGYFIPKNTSVCIHIYKVHHD
PEHFSQPETFNPDNFLPENIAKRHPFSYIPFSAGARNCIGQKFAIQEIKLILSSILRNFKVISLTNQHDV
QIQSAFILTSKEGLKFRLVPRL

>ON646312 CYP4415A1 [529 AA] TRINITY_DN44522_c0_g1_i1|m.17308
MYSRIAGTLLNYKFFNKLTIYLGLVVVCIFLFVKIKNTLLRQWKIYIQLRWLPGPEKTVPFLGNSY
MFFNCNTADIYNVFVKLINKYGRKSAFKFWVGNKVFIILTKPEDLRILYNSQYAMEKEHTLYQMF
SEWLGNDSLLIAPVEKWKAHRRILAMTFSQRVLDNFVQIFSKNSVNLSNELQSYADNKEIFNVYN
VINKYTVKSICGTLMGVEIKDEHFVQKYLTAVQKEAEIIYLKMCKFWLHFNIIYKFSKMYEDETK
YLKILHSFSKMVFMKKNISYSNENSFSSSSKKFGINPKRKPFLELLLDLKRNCIHYDQNQILDDMNI
FLAAGTETTSITSSFVLLMLGIHQNIQDKVYEELNRILNDDKNEHEKNKNRMITPDDIRQMFYLEM
VIKETLRLYPIVPIVARTITKDLMLPTSGTIPSGCCCIAAPIAVHYDPEIWTQPLLFNPDRFLSEEKIK
RHIYSFIPFSGGLRNCIGKKFAFMSLKVLIASIIRDYQILTPLKMEDIQLKVEVTLKSVNVTSTFN

>ON646313 CYP4415A1 [533 AA] TRINITY_DN44522_c0_g1_i3|m.17311
MYSRIAGTLLNYKFFNKLTIYLGLVVVCIFLFVKIKNTLLRQWKIYIQLRWLPGPEKTVPFLGNSY
MFFNCNTADIYNVFVKLINKYGRKSAFKFWVGNKVFIILTKPEDLRILYNSQYAMEKEHTLYQMF
SEWLGNDSLLIAPVEKWKAHRRILAMTFSQRVLDNFVQIFSKNSVNLSNELQSYADNKEIFNVYN
VINKYTVKSICGTLMGVEIKDEHFVQKYLTAVQKEAEIIYLKMCKFWLHFNIIYKFSKMYEDETK
YLKILHSFSKMVFMKKNISYSNENSFSSSSKKFGINPKRKPFLELLLDLKRNCIHYDQNQILDDMNI
FLAAGTETTSITSSFVLLMLGIHQNIQDKVYEELNRILNDDKNEHEKNKNRMITPDDIRQMFYLEM
VIKETLRLYPIVPIVARTITKDLMLPTSGTIPSGCCCIAAPIAVHYDPEIWTQPLLFNPDRFLSEEKIK
RHIYSFIPFSGGLRNCIGKKFAFMSLKVLIASIIRDYQILTPLKMEDIQLKVEVTLKSVNGYPIQLVR
RR

>ON646314 CYP6YH4 [370 AA] TRINITY_DN44806_c1_g3_i1|m.18595
MRNMFTLLDGFGEDLNKYIKNHVNKTIEVKDACRKYTTDVIVSTAFGIQANSFSDEEAEFSAISKK
LFTFTLRRSVELICFFFVPELVTLFNFKLFSSFGTAFLRNAVSQAIEVREKNNINRPDLIDALIKLKNT

GTLEDSPDLNDKQDHNKATIKFEGDILAAQAAVFFTAGFETTSSAISFTLYFLSYNPDIQIRLRKEIQ
TVIAENSGKFTYDSLAEMKYLDLCIKETLRLYPSLGFLDREAMSTYTFPGTDLTIDKGTPVIISLTGL
HRDPQYFENPEVYDPERFNDENKSKFPPFAYIPFGEGPRNCVGARFGMVATKVGLVHILKDFEVV
TFKTAPTLEIDPRGFLTSPKHGINLNFVKGTVYV

>ON646315 CYP6YH4 [520 AA] TRINITY_DN44806_c1_g3_i2|m.18597
MALISSSWYTDLLTLTTGLLTILYFYLTSTYDYWKKRKVPYDPPVLLFGNFYNAFTFKRNISSYFY
DLYRKTNEKYFGFYIYRRPYLLLRDPELIKHVLVKDFNNFVPRTNADTTNKEDVLGRDNIFTIKDP
HNWRYLRAKFSPFFSTGKMRNMFTLLDGFGEDLNKYIKNHVNKTIEVKDACRKYTTDVIVSTAF
GIQANSFSDEEAEFSAISKKLFTFTLRRSVELICFFFVPELVTLFNFKLFSSFGTAFLRNAVSQAIEVR
EKNNINRPDLIDALIKLKNTGTLEDSPDLNDKQDHNKATIKFEGDILAAQAAVFFTAGFETTSSAIS
FTLYFLSYNPDIQIRLRKEIQTVIAENSGKFTYDSLAEMKYLDLCIKETLRLYPSLGFLDREAMSTY
TFPGTDLTIDKGTPVIISLTGLHRDPQYFENPEVYDPERFNDENKSKFPPFAYIPFGEGPRNCVGARF
GMVATKVGLVHILKDFEVVTFKTAPTLEIDPRGFLTSPKHGINLNFVKGTVYV

>ON646316 CYP304W1 [510 AA] TRINITY_DN45051_c0_g2_i5|m.20044
MVSPLIVVIAITYFAYWILEFLLARPKNFPPGPIRIPIFGSYLYLLLRDVKHFYVETQRWCDKYNSK
VVALFIGSYNAVSVNDLEGIRRVFLCNDFDGRPFPYSVQLRSWAKRLGVFFTEGPFWLEQRRFTL
RYMRDFGFGRRMERLEYIIEDETKLMIKLIEEGTKNPYNAGLVKDGRLIYLPEFFAAPFINSYLAVL
TGHPLPEESRPILRKTTQSSILFQLAADATGGIISILPSLRYIFPKLSGYHGITKGHEALMDLMRDIIK
QRKNRGFTETEGFIDFYLNEMKARKEAGGYTTFSDDQLLLIVADLLMPASSAVQSQLGMLVERLI
HNPEIQDKIYNEIKTVVGSGRLPTLDDRPNLPYCEATLRESLRIDTLATLGVPHSTLVDTTLHGYSIP
KDTIVITNLWQMHHDEKVWGDPETFRPERFVTEDGKGLAKDQSLPFGLGRRVCAGETFARNTMF
LIYTGLLQNYKLTVPEGHPLPDLNDRLNGLVISPKDFWVKCIPRK

>ON646317 CYP4XH1 [461 AA] TRINITY_DN45641_c0_g1_i3|m.23169
TIIILILSLIVGKYIEYRWKNRRFFKLAAKFNGPDPLPFIGNAHMFSGTSEDVFKQILDHFYNYNDKG
PSRFWLGNQLYVVITKPQHMKAVLTNPNALQKSYVYSFINTWLGTGLFSAPVTIWKHHRKIIMPS
FNQKILDTFISIFQERSNVLAKKLECETGYFDIFPYVSRCTLDSICESALGITVNSLEDSNEKYVECA
TRVLDIVNTRMLKVWLHPDFIFKRTKLYRELQFHIQYLHNFTDSIIKQKRAEFKEIQESNRFSPTDQ
TNDEPIFKRKAFIQLLMETSDFTDEEIRNEVDTMTVAGNDTTATVNSFTLLLLAMYPDIQEKLYNE
VYSIVGDSDKTIELEDLPKLKYTEMVIKESMRLFPIGPILGRDALEDIQLDENYTIPKGSGIALGIYY
THRDPEYWNEPDVFDPERFQPEELKTRDPYSYLPFSGGPRNCLGKILLFLCQKTGTSPV

>ON646318 CYP4XH1 [535 AA] TRINITY_DN45641_c0_g2_i1|m.23171
MVLPAFCSIFFATYDWVSDSKEHSTSANYHIFPTITILILSLIVGKYIEYRWKNRRFFKLAAKFNGPD
PLPFIGNAHMFSGTSEDVFKQILDHFYNYNDKGPSRFWLGNQLYVVITKPQHMKAVLTNPNALQ
KSYVYSFINTWLGTGLFSAPVTIWKHHRKIIMPSFNQKILDTFISIFQERSNVLAKKLECETGYFDIF
PYVSRCTLDSICESALGITVNSLEDSNEKYVECATRVLDIVNTRMLKVWLHPDFIFKRTKLYRELQ
FHIQYLHNFTDSIIKQKRAEFKEIQESNRFSPTDQTNDEPIFKRKAFIQLLMETSDFTDEEIRNEVDT
MTVAGNDTTATVNSFTLLLLAMYPDIQEKLYNEVYSIVGDSDKTIELEDLPKLKYTEMVIKESMR
LFPIGPILGRDALEDIQLDENYTIPKGSGIALGIYYTHRDPEYWNEPDVFDPERFQPEELKTRDPYSY
LPFSGGPRNCLGIKYAYMAMKILIATVIRHYKLSTDLKLDDLILKTELTLKLSNAFLLRIQKRQTMS
Q

>ON646319 CYP4XH1 [372 AA] TRINITY_DN45641_c0_g1_i6|m.23173
MPSFNQKILDTFISIFQERSNVLAKKLECETGYFDIFPYVSRCTLDSICESALGITVNSLEDSNEKYV
ECATRVLDIVNTRMLKVWLHPDFIFKRTKLYRELQFHIQYLHNFTDSIIKQKRAEFKEIQESNRFSP
TDQTNDEPIFKRKAFIQLLMETSDFTDEEIRNEVDTMTVAGNDTTATVNSFTLLLLAMYPDIQEKL
YNEVYSIVGDSDKTIELEDLPKLKYTEMVIKESMRLFPIGPILGRDALEDIQLDENYTIPKGSGIALG
IYYTHRDPEYWNEPDVFDPERFQPEELKTRDPYSYLPFSGGPRNCLGIKYAYMAMKILIATVIRHY
KLSTDLKLDDLILKTELTLKLSNAFLLRIQKRQMMSQ

>ON646320 CYP6YH5 [528 AA] TRINITY_DN45681_c1_g1_i1|m.23301

MSFITSNWLTNCLPILTFVLTVLYIYLNSKYNYWKKRNVPYEKPNLIFGNFYETFTLKKNLTTFFQE
QYEKTNERVYGLYIFQRPYLLLRDPELAKYVLVKDFNNFIARSNSEAPNDSLGKNNLFSLKNIHH
WRFMRTKMSPFFSPSKMRNLFELFDEIGVQLTEHIKKHVNETMDVKDVSQKYTTDVIVSAAFGIQ
ANSFTSDQAEFSGIAKIMFTYTLKRSIELFSFFFAPLLVKIFNFKFFGEEASQFLRKVVWHTIQVREE
NNIKRPDLIDALITLKTTGTMEDSLVSEKNVGESGIKLQDSIELEGDVLVAQAAVFFTAGFGTSSLT
MSFTLHSLSYNPEIQMKLRKEIQSVIARNGGKFTYDCLQEMKYLDACVKETLRLYPTLGMLDREA
INTYTFPGTDITIDKGTAVVISLGGLHRDPQYFENPYVYDPERFNDDNKNRIKPFTYMPFGEGPRNC
IGARFGSVATKVGLVHILKDFELETYEKPPPLEVNPRSVFLSPKNGIHLKLRTGTVYEQLNEVD

>ON646321 CYP6YH5 [448 AA] TRINITY_DN45681_c1_g1_i4|m.23306
IFQRPYLLLRDPELAKYVLVKDFNNFIARSNSEAPNDSLGKNNLFSLKNIHHWRFMRTKMSPFFSP
SKMRNLFELFDEIGVQLTEHIKKHVNETMDVKDVSQKYTTDLIVSAAFGIQANSFTSDQAEFSGIA
KIMFTYTLKRSIELFSFFFAPLLVKIFNFKFFGEEASQFLRKVVWHTIQVREENNIKRPDLIDALITLK
TTGTMEDSLVSEKNVGESGIKLQDSIELEGDVLVAQAAVFFTAGFGTSSLTMSFTLHSLSYNPEIQ
MKLRKEIQSVIARNGGKFTYDCLQEMKYLDACVKETLRLYPTLGMLDREAINTYTFPGTDITIDK
GTAVVISLGGLHRDPQYFENPYVYDPERFNEDNKNRIKPFTYMPFGEGPRNCIGARFGSVATKVG
LVHILKDFELETYEKPPPLEVNPRSVFLSPKNGIHLKLRTGTVYEQLNEVD

>ON646322 CYP4416C1 [467 AA] TRINITY_DN45691_c0_g1_i3|m.23335
MFLKLFLNCFQNNSQIGLVLLVLVIVLIYMYNTNYLRIVYYLSKIPGPKSYPIIGVAYLVIGKTHAEI
TQLVIDMINKFSPILKAYLGPIPLVCVSSPEYVREILTNHHGLEKPWLMTHIAVDVAGDSILSAQVP
KWKHNRSKIVRGFTPNILKTYFKVMIESSLLLSKKLEDKLNDSKSFDILEYLSKAAMDSVARSTMG
VNVNVQDSGSTFYGAMQSMFHIAFDRIYSPWKLLDVIFRLSQDFPRFSQDKKAVIELAEKIISEKR
YQKTCINNNDNKSDNIETIDKELSEKSLLDILLEVTKNDPLFDDQQLKDEVIFMIIAGFETTANALG
YVLLNLAVHQDIQNKVCEELFQVVDDFDRKIHLDDLPQLKYMEMVIKESMRLHPTIPLIARKLTR
DIKFDEYLVPDGTTVVIPIICLHRNENIWTDPLKFDPERFTPENSKNRHPYAFLPFSAGPRNCVGKG
N

>ON646323 CYP4416C1 [518 AA] TRINITY_DN45691_c0_g1_i5|m.23337
MFLKLFLNCFQNNSQIGLVLLVLVIVLIYMYNTNYLRIVYYLSKIPGPKSYPIIGVAYLVIGKTHAEI
TQLVIDMINKFSPILKAYLGPIPLVCVSSPEYVREILTNHHGLEKPWLMTHIAVDVAGDSILSAQVP
KWKHNRSKIVRGFTPNILKTYFKVMIESSLLLSKKLEDKLNDSKSFDILEYLSKAAMDSVARSTMG
VNVNVQDSGSTFYGAMQSMFHIAFDRIYSPWKLLDVIFRLSQDFPRFSQDKKAVIELAEKIISEKR
YQKTCINNNDNKSDNIETIDKELSEKSLLDILLEVTKNDPLFDDQQLKDEVIFMIIAGFETTANALG
YVLLNLAVHQDIQNKVCEELFQVVDDFDRKIHLDDLPQLKYMEMVIKESMRLHPTIPLIARKLTR
DIKFDEYLVPDGTTVVIPIICLHRNENIWTDPLKFDPERFTPENSKNRHPYAFLPFSAGPRNCVGIRY
AWMFMKVVLATLLSRYEFHTDVDVNKLEHEFALSLKLLGGHQVRITPRRRN

>ON646324 CYP4416C1 [518 AA] TRINITY_DN45691_c0_g1_i11|m.23341
MFLKLFLNCFQNNSQIGLVLLVLVIVLIYMYNTNYLRIVYYLSKIPGPKSYPIIGVAYLVIGKTHAEI
TQLVIDLINKFSPILKAYLGPIPLVCVSSPEYVREILTNHHGLEKPWLMTHIAVDVAGDSILSAQVP
KWKHNRSKIVRGFTPNILKTYFKVMIESSLLLSKKLEDKLNDSKSFDILEYLSKAAMDSVARSTMG
VNVNVQDSGSTFYGAMQSMFHIAFDRIYSPWKLLDVIFRLSQDFQRFSQDKKAVIELAEKIISEKR
YQKTCINNNDNKSDNIETIDKELSEKSLLDILLEVTKNDPLFDDQQLKDEVIFMIIAGFETTANALG
YVLLNLAVHQDIQNKVCEELFQVVDDFDRKIHLDDLPQLKYMEMVIKESMRLHPTIPLIARKLTR
DIKFDEYLVPDGTTVVIPIICLHRNENIWTDPLKFDPERFTPENSKNRHPYAFLPFSAGPRNCVGIRY
AWMFMKVVLATLLSRYEFHTDVDVNKLEHEFALSLKLLGGHQVRITPRRRN

>ON646325 CYP4416C2 [495 AA] TRINITY_DN45691_c0_g1_i7|m.23338
MFLKLFLNCFQNNSQIGLVLLVLVIVLIYMYNTNYLRIVYYLSKIPGPKSYPIIGVAYLVIGKTHAEI
TQLVIDLINKFSPILKAYLGPIPLVCVSSPEYVREILTNHHGLEKPWLMTHIAVDVAGDSILSAQVP
KWKHNRSKIVRGFTPNILKTYFKVMIESSLLLSKKLEDKLNDSKSFDILEYLSKAAMDSVARSTMG
VNVNVQDSGSTFYGAMQSMFHIAFDRIYSPWKLLDVIFRLSQDFPRFSQDKKAVIELAEKIISEKR
YQKTCINNNDNNSDNIESIDKELSGKSLLDILLEVTKNDPLFDDQQLKDEVIFMIIAGFETTANALG
YILLNLAVHQDIQNKVCEELFQVLDDSDRKIELDDLPQLKYMEMVIKESMRLHPTIPLIARKITRDI
KFDEYLVPDGTTVVIPIICLHRNENIWTDPLKFDPERFTPENSKNRHPYAFLPFSAGPRNCVGIRYA
WMFMKVVLATLLTRYEFHTDIDVNKLEH

>ON646326 CYP315A1 [486 AA] TRINITY_DN45615_c0_g2_i2|m.23475
MINRIVSRTISTANNKLINNNNANATVQQLLSQIPQPQTLPIIGSTLALLLSGGSAKLHEYIDKRHQQ
LGNIFIDQLGPTKCVFILDPNDMRKVFNNEGKYPAHIYPEAWKLYNQLKNCERGLFFMDGTEWL
VFRRIMNKTLLREDLETFYEPCRIVADNVTNIWLESQQTEVLNLHDILYRWSVESVTSILLGTHYM
KNHKKLQPSITKLVRVMQDVFETTSKISLIPANFAYKFKLPAWRRFETAVDLALERANALSSMILD
MHENGGGVLERLLENGISKKDSIRVMTDLLLAAGDTTAHTMEWLLYLLAKNPNIQNDIYRESLTV
PDMSDPPALLKGAVREALRLYPVAPFLNRILPNDINIQGYDIPAGTLLILSLYSSGRCEKYFNSPEKF
WPERWLRCDLGESMNYSNLRAAVAHASMPFAMGSRSCIGKKLAEAQMYVTIRMILNKFSLELLN
TNNIEMVMRLVGVPSEPIRIALHKR

>ON646327 CYP4419A1 [493 AA] TRINITY_DN45829_c1_g2_i1|m.24448
MSPVLVFCIVLLVLYILIKYNWDRRDLYKLSWKLEGPFALPIIGNAFFFLFNSIEDTIPIAFDLLKKY
NGILRGWMGPQLWIFTANPNDIQHILSSQKITHKGAAYRFFNPIFGQGLINNNGQLWRMHRKIIVK
SFSQDFLSEYMHIFHEQSKVMVANMQKEVGKPAFDVMKYVQLCAMDIVGEAVMDVKLNVQSE
HNMEVVSSLDNIYMLIHDRILKYWLHPDFIFENTAKGKLQHKVVNYLRNFIQKNVLRKQEEVSNN
NSIKCKSTLETILHTLNDNPEAMSKQGIIDEMITLFAAGEDSISLQFSWTLLLLALHPEIQDKVYNEI
QSVLGDNENFGRDDLKKLYYTTMVIKEVSRLFPVAPFIVREVTEDFPIDKVVLPRGTSVFIGVMYV
HRDPSHWEYPNKFYPEHFLPEAVAKRHPFAFMPFSAGARGCIGKEFAMLCMKSMLVTILRNFQLQ
ADGTINDIKLKVDIIARSMNGYNLRLVQRINTL

>ON646328 CYP9GK1 [384 AA] TRINITY_DN45823_c2_g2_i1|m.24474
IGLFYWKFVRPLSLWKDKGLPYVKGYPIVGNYLGPVLGRLSFAEAIQVYYNATSEEKCAGIFVFN
QPQLMIRDPQLIKQMFIKDFDHFTDHTTVFFTEGFDTIFDKNLFALKGNDWRDMRATLSPAFTGV
KMRGMFQVMNECGEQVYHHYSNELKALKDSSHLELDLKDLFTRFTNDIIASTAFGVKCDSFKND
KNDFFVMGRKATTISTLRFLLVNFMPAVMKLLRLSVIPIYVCDYFRNVVKETTEIRVRENIVRPDM
LNLLMEAKKGALKVEEQKEENRGFAVADEYNNHHGKIKILNDDDVAAQAFIFFFAGFETVSTAM
CFLGYELALNPDIQERVYAEIQDVLESNNGKLSYDILNKMKYLDMCVSESLRKWPPAVLT

>ON646329 CYP4413A1 [407 AA] TRINITY_DN45909_c0_g1_i6|m.24917
MFLIFLAITFILFIVFDTIFRFQNRKLSLLAKQVPGPWGLPFLGMWYTVIDGPKGFLRLTSNLVKKY
GNICRAWMGYSLYLVVSKPEDIEAILTNSVEKDYYLKFTSIFAGDGLFIAPAQIWKHRRKLINPTFN
TKIVNSFHEKMVAKSNVLCKKLETRTSEDNYFDCYDSLFACTLDILFETNLDFSVDFQNGQLPYYL
KYMKRITRLVVLWMGKFWLHTNITFNHSNFGKKLMQETQYMKRISANIIQEKTVEIRKKMSDEN
YTGNKVQKPFLEHIIYAKESGFPISETSVVNEVDTMIIAGSDTSAYVMSFALMTLGVLQDIQDKVH
EEIISVFGNSDRECTLDDIKSMQYLEMVIKETMRLFAPTPLLARKVTTDVQLRKLSQFKTSKNKQL
TELIIEIPCIV

>ON646330 CYP4413A1 [494 AA] TRINITY_DN45909_c0_g3_i3|m.24923
MFLIFLAITFILFIVFDTIFRFQNRKLSLLAKQVPGPWGLPFLGMWYTVIDGPKGFLRLTSNLVKKY
GNICRAWMGYSLYLVVSKPEDIEAILTNSVEKDYYLKFTSIFAGDGLFIAPAQIWKHRRKLINPTFN
TKIVNSFHEKMVAKSNVLCKKLETRTSEDNYFDCYDSLFACTLDILFETNLDFSVDFQNGQLPYYL
KYMKRITRLVVLWMGKFWLHTNITFNHSNFGKKLMQETQYMKRISANIIQEKTVEIRKKMSDEN
YTGNKVQKPFLEHIIYAKESGFPISETSVVNEVDTMIIAGSDTSAYVMSFALMTLGVLQDIQDKVH
EEIISVFGNSDRECTLDDIKSMQYLEMVIKETMRLFAPTPLLARKVTTDVQLPNVVVPANTNCIICV
LELHRSSDIYSDPLKFDPNRFLPEETAKRHPYAYIPFGGGARGCIGPKFAYLSMKIILTSILRKYKIYS
DVNLENMELKMELFLKPVNGFRIRLEKR

>ON646331 CYP4413A1 [423 AA] TRINITY_DN45909_c0_g3_i5|m.24926
MSYNSLRCRIRADATLHMAILTNSVEKDYYLKFTSIFAGDGLFIAPAQIWKHRRKLINPTFNTKIVN
SFHEKMVAKSNVLCKKLETRTSEDNYFDCYDSLFACTLDILFETNLDFSVDFQNGQLPYYLKYMK
RITRLVVLWMGKFWLHTNITFNHSNFGKKLMQETQYMKRISANIIQEKTVEIRKKMSDENYTGNK
VQKPFLEHIIYAKESGFPISETSVVNEVDTMIIAGSDTSAYVMSFALMTLGVLQDIQDKVHEEIISVF
GNSDRECTLDDIKSMQYLEMVIKETMRLFAPTPLLARKVTTDVQLPNVVVPANTNCIICVLELHRS
SDIYSDPLKFDPNRFLPEETAKRHPYAYIPFGGGARGCIGPKFAYLSMKIILTSILRKYKIYSDVNLE
NMELKMELFLKPVNGFRIRLEKR

>ON646332 CYP9GK6 [525 AA] TRINITY_DN45934_c4_g3_i2|m.25022
MWLITFSLVGVFYFYWNFIRPLNTWKNYGIPYIKGWPLVGNFLKNALKRESFGDAVISMYKAFPN
EPYCGSFVFSAPRLLIRDANLLKDIGIKDFDHFTDHTKIFFGDGFDPIFDKNLFSLDGQNWRDMRAT
LSPAFTGSKMRGIFVLMNECGNQLVNYLNDKIKTENNQKHLELELKDLFTRFTNDIIATTAFGIKV
DSLKEPENKFYLMGREVTYFSTFRLFLMNFIPTIMRLLNIGILSKAACGYFRNLVFQTISMRETQKI
VRPDMLNILLQTKNGLLKNDDKGDDNGGFAAATEGKVGGKIRKLTDEDVTAQAFIFFLAGFDTV
STAMCFTVYELALNPDIQQRLYSEINDALDGNPNAEVTYETLHRMKYLDMIISESLRKWPPAVFID
RECTKPYRLKTDNGKEIMIPTDMGIVIPIMGIHRDPKYYPNPEKFDPERFSDENKSKINMNAYLPFG
VGPRVCIGSRFALMETKILIVHLIINFEVILTSKSTVPPRLSTQSSNMAIENGFNVGLKPRNF

>ON646333 CYP304V1 [513 AA] DN46134_c0_g2_i1|m.26206
MSLIIFILLIILIFLIIYLLIKNAYTRPENFPPGPPRLPIWGSYWILLALNYKFPHLALRELGKRYKTNV
LGFYLGQTPAVITLNYETTHEMLTRSEFTNRQVNFVIKKRAFGQENLGIFFSYGDFWKEQRRYSLR
TMRDFGLGRRSPNLENVVADEVKKLMTMICNGPSNEIEKTVCKEKFHILLPHGLFSGFFNAFWYV
LTGDTYSDPYKSHKVFQNALKFQRSGEPLGGALVYTPWLRYIWPNKSGHVPTFESHFELVDFFKD
YIKEIKENLSEDHSTGYIHEYLLELKKQNPETYSTFSEDQLLLTLVDHLIPSIVANTTVLAFTFLRILH
NPHVQTKIQQEIDTVVGSGRYPTLDDRSKMIYTEATIKEGLRMDALNPLGIPRECNQDTYFQGYFI
PKGTFMVPGNHSSNMDTKLWGDPENFRPERFIDTNGHLVKKDYALLFGAGKRVCVGETFSRQN
MFLFLSMLMQNFQFSVPDGESLPETTYQNYINGINVAPKEFWIYAKPRF

>ON646334 CYP4416D2 [515 AA] TRINITY_DN46460_c4_g2_i1|m.27900
MFLSLIINFIFNHILVTLIVLIIILICVYFSNYFRICYYVSKINGPRAYPIIGNSTLFRGTLEEATKVFFQL
SNEYAPITRFWIGPVPLVIVMDPKIIKEILYNQNALEKAWFMKLIMKCVLGNTILISEVPKWKRNR
NIINQGFTLSILKSYFKIFEGNIDTLSDILDNELNNERFFDIYGILSKTTLEAVCASSMRLDMKFQQD
QSNSSYFEAIHSSMHMMSARLMNPLKLPNIFYQFTQDYKKLMGYKNEVYAIARDAISTKRKEQIT
MDDTLIDFKSENNNEIKKPLLDYLIDFTKSNPDFTDEEMADEINLTIIAAYETTARTVSMVLLILAL
YKDMQNKVCEELFQMLGESNENKINFDDLSKLKYMEMVIKEALRLFPAIPLIARQLTEDVTMGN
YTIPKGTNIGIPIFALHRNEAYWEEPLKFDPERFTFENIKKQNPYAFIPFSAGPRNCLGVRYAWIFM
KLALSRLLSKYEFHTNIKDLSEIKFEFEISLKIINGYPIRITPRQR

>ON646335 CYP4C163 [501 AA] TRINITY_DN46558_c1_g1_i2|m.28640
MFCMYAVLFLIAAIILLWHKNKNDRTVKLIEQFPGPKKLPIVGTSWTLLGVPVNKLFSYISSEYDR
YYPGPYRTWLGPTPAINIYKPEHIECIVNNQKHITKGLTYNFLHPWLGQGLLTSSGEKWFKHRRMI
TPAFHFKILDQFVEVFFEKCDIMTKRLERHSDGKYFDIYPYITRCALDIICETAMGVPINAQVDQDS
EYVTALYEVSDLTMKRSVLPWLQPDIIWNNVADGKRYYQCLAILHGFTNSVIKGRRESRKSNVAE
TVSKDDDIGSKKRMAFLDVLLEAQEKDPTLSDTDIREEVDTFMFEGHDTTTAAICWSLFMLGLHP
EVQEKAYQEIKQIFQGSNRSLTKQDLLEMKYLEMVIKEALRLFPSVPIIGRVLKEDQEIDGKLVPKE
TVIFVNIHKSQRDPAHFNNPNEFNPDNFLLENSSKRHPYAYMPFSAGPRNCIGQKFALLEEKTIISTI
LRNFKLKSMQKMHEVNLVGSLVLRPEQGAIVSLEKRQ

>ON646336 CYP4C163 [372 AA] TRINITY_DN46558_c1_g1_i5|m.28641
MFCMYAVLFLIAAIFVLWHKNKNDRTVKLIEQFPGPQKLPIVGTSWTLLGVPVNKLFSYISSEYDR
YYPGPYRTWLGPTPAINIYKPEHIECIVNNQKHITKGLTYNFLHPWLGQGLLTSSGEKWFKHRRMI
TPAFHFKILDQFVEVFFEKCDIMTKRLERHSDGKYFDIYPYITRCALDIICETAMGVPINAQVDQDS
EYVTALYEVSDLTMKRSVLPWLQPDIIWNNVADVKRYYQCLAILHGFTNSVIKGRRESRKSNVAE
TVSKDDDIGSKKRMAFLDVLLEAQEKDPTLSDTDIREEVDTFMFEGHDTTTAAICWSLFMLGLHP
EVQEKAYQEIKQIFQGSNRSCPSNIKVSTSSRISVSLNVGSFS

>ON646337 CYP4C163 [396 AA] TRINITY_DN46558_c1_g1_i6|m.28643
MFCMYAVLFLIAAIILLWHKNKNDRTVKLIEQFPGPKKLPIVGTSWTLLGVPVNKLFSYISSEYDR
YYPGPYRTWLGPTPAINIYKPEHIECIVNNQKHITKGLTYNFLHPWLGQGLLTSSGEKWFKHRRMI
TPAFHFKILDQFVEVFFEKCDIMTKRLERHSDGKYFDIYPYITRCALDIICETAMGVPINAQVDQDS
EYVTALYEVSDLTMKRSVLPWLQPDIIWNNVADGKRYYQCLAILHGFTNSVIKGRRESRKSNVAE
TVSKDDDIGSKKRMAFLDVLLEAQEKDPTLSDTDIREEVDTFMFEGHDTTTAAICWSLFMLGLHP
EVQEKAYQEIKQIFQGSNRSLTKQDLLEMKYLEMVIKEALRLFPSVPIIGRVLKEDQEIGKIYDNKT

>ON646338 CYP4C163 [501 AA] TRINITY_DN46558_c1_g1_i7|m.28644

MFCMYAVLFLIAAIFVLWHKNKNDRTVKLIEQFPGPQKLPIVGTSWTLLGVPVNKLFSYISSEYDR
YYPGPYRTWLGPTPAINIYKPEHIECIVNNQKHITKGLTYNFLHPWLGQGLLTSSGEKWFKHRRMI
TPAFHFKILDQFVEVFFEKCDIMTKRLERHSDGKYFDIYPYITRCALDIICETAMGVPINAQVDQDS
EYVTALYEVSDLTMKRSVLPWLQPDIIWNNVADGKRYYQCLAILHGFTNSVIKGRRESRKSNVAE
TVSKDDDIGSKKRMAFLDVLLEAQEKDPTLSDTDIREEVDTFMFEGHDTTTAAICWSLFMLGLHP
EVQEKAYQEIKQIFQGSNRSLTKQDLLEMKYLEMVIKEALRLFPSVPIIGRVLKEDQEIDGKLVPKE
TVIFVNIHKSQRDPAHFNNPNEFNPDNFLLENSSKRHPYAYMPFSAGPRNCIGQKFALLEEKTIISTI
LRNFKLKSMQKMHEVNLVGSLVLRPEQGAIVSLEKRQ

>ON646339 CYP12AR1 [532 AA] TRINITY_DN46620_c3_g1_i1|m.28748
MNKKLVSFHEIPFKFQQLRNLTVDKNINNKLTTENKKYHDLEWNEALPFEFLPGPKPLPIIGNAWR
FFPFIGNYYGKNILELHKQLHKEYGNIVILKGIMGRPPFVITFSPEDCEKIFRNEGTRPYRNGIGSLA
YYQKVVRKNIYKNNASLAVLQGDEWHKMRAQVNSALMQPRVVARYVNRIENVARDFILKIDFL
LKNSKNTDELPDDFLNEIYRWSLETAGEISLDTRLGCLELNVSQNSEVQQIIDAVNDMFDLIFELNI
LPSMWRQISTPKYKKFVRTMDYLTSICRKYADRAQTKMLGSEHNNDTVPSVLEVLLKVDKVVAII
MAVDILLAGVDTTGKTAAAVMYFLSINQEKQDKLREELIELMPDPNTPLTRQILDKMTYLRGCIK
EAQRIASITPGQNRVLPKDITLSGYQIPKNTEVFTGNLLMCNLDENYPKSNQYIPERWLRNSAPELT
AKTPHPFVFLPFGHGPRACIGRRFAFCELEVLIATVFRKYKFEWHHDEMKFNSRLFYGVDSPLKFK
LIPIY

>ON646340 CYP12AR1 [452 AA] TRINITY_DN46620_c3_g1_i2|m.28749
MYVCILHKEYGNIVILKGIMGRPPFVITFSPEDCEKIFRNEGTRPYRNGIGSLAYYQKVVRKNIYKN
NASLAVLQGDEWHKMRAQVNSALMQPRVVARYVNRIENVARDFILKIDFLLKNSKNTDELPDDF
LNEIYRWSLETAGEISLDTRLGCLELNVSQNSEVQQIIDAVNDMFDLIFELNILPSMWRQISTPKYK
KFVRTMDYLTSICRKYADRAQTKMLGSEHNNDTVPSVLEVLLKVDKVVAIIMAVDILLAGVDTT
GKTAAAVMYFLSINQEKQDKLREELIELMPDPNTPLTRQILDKMTYLRGCIKEAQRIASITPGQNR
VLPKDITLSGYQIPKNTEVFTGNLLMCNLDENYPKSNQYIPERWLRNSAPELTAKTPHPFVFLPFG
HGPRACIGRRFAFCELEVLIATVFRKYKFEWHHDEMKFNSRLFYGVDSPLKFKLIPIY

>ON646341 CYP12AR1 [371 AA] TRINITY_DN46620_c3_g1_i3|m.28750
MRAQVNSALMQPRVVARYVNRIENVARDFILKIDFLLKNSKNTDELPDDFLNEIYRWSLETAGEIS
LDTRLGCLELNVSQNSEVQQIIDAVNDMFDLIFELNILPSMWRQISTPKYKKFVRTMDYLTSICRK
YADRAQTKMLGSEHNNDTVPSVLEVLLKVDKVVAIIMAVDILLAGVDTTGKTAAAVMYFLSINQ
EKQDKLREELIELMPDPNTPLTRQILDKMTYLRGCIKEAQRIASITPGQNRVLPKDITLSGYQIPKNT
EVFTGNLLMCNLDENYPKSNQYIPERWLRNSAPELTAKTPHPFVFLPFGHGPRACIGRRFAFCELE
VLIATVFRKYKFEWHHDEMKFNSRLFYGVDSPLKFKLIPIY

>ON646342 CYP12AR1 [520 AA] TRINITY_DN48207_c3_g1_i3|m.38225
MNKKLVSFHEIPFKFQQLRNLTVDKNINNKLTTENKKYHDLEWNEALPFEFLPGPKPLPIIGNAWR
FFPFIGNYYGKNILELHKQLHKEYGNIVILKGIMGRPPFVITFSPEDCEKIFRNEGTRPYRNGIGSLA
YYQKVVRKNIYKNNASLAVLQGDEWHKMRAQVNSALMQPRVVARYVNRIENVARDFILKIDFL
LKNSKNTDELPDDFLNEIYRWSLETAGEISLDTRLGCLELNVSQNSEVQQIIDAVNDMFDLIFELNI
LPSMWRQISTPKYKKFVRTMDYLTSICRKYADRAQTKMLGSEHNNDTVPSVLEVLLKVDKVVAII
MAVDILLAGVDTTGKTAAAVMYFLSINQEKQDKLREELIELMPDPNTPLTRQILDKMTYLRGCIK
EAQRIASITPGQNRVLPKDITLSGYQIPKNTEVFTGNLLMCNLDENYPKSNQYIPERWLRNSAPELT
AKTPHPFVFLPFGHGPRACIGRRFAFCELEVLIATVRCQYIRLPEVELYTEWAILINNG

>ON646343 CYP4418B2 [368 AA] TRINITY_DN46696_c0_g1_i1|m.28800
MWVDLLLFVALLIGIFTIFYTWNYDYWNKRRLPYVKPIFPFGSLKDVILFKKTFSSLLDDWYYKY
KDYKYIGVYFGRHPVLFIRDAQLIKDVMVKHFNHFQDNNARINEKADPIAGNNPFMLKGGRWKS
TRAHLTPGFTALKLKQMFPNMLSVSERLISYIQQNIDSSKNSIEAKDLSTNFTTEVFTLAALNMKSD
SFENPNSQFKTIAGDLLAGSHLKLFITWTLTIVDPALAVLLRIRILSKKGEMYFRKAISDVLAYREQ
NNLVINDFCNYLNNIHLKNSDFTRDDVVSNIVTIFLDGVETSSQIMRFLLREVAAYPEVFKKLRNEI
DTIKSKYDDKITFDGLQEMTYLDSCLFETLRIYSALLT

>ON646344 CYP4422A1 [409 AA] TRINITY_DN46720_c0_g1_i4|m.29972

MFLILSLVLVIYSLIYYYFRHKFSYWKRRGVETYPARFPFGHLGEFYLNKKSYIEIFTQYYDQSKSE
FCGIYQGIDPLLIVKNLEDIKYVLVKNFNCFMNRGVHTDFNSNVVSATLFRIEGDQWRNLRHKLTP
AFTSGKLKAMFETINQCCNGLDYALNKAMDPKTGEINVKMLAQRYTVDVISSVGFGVEANCLND
NYASPFFKLSQEILDRTALRTLLIKFQFFGPSYAKILYKFFSDRSKENQQSFFNMINSIVENRQKNKI
MRKDFLQLLIELKNHGTIDSSNDSIDNPNVFDKKDSLNFEFDMKTFVSQVFIFYFAGFDTSALTLT
MMLYYLAENQDLQKRLRKDINDCLRKHNNKLTYDGVMEMEYLEMLLSETLLLNLNWASSILYY
ILETNAPVLALNVMF

>ON646345 CYP4422A1 [439 AA] TRINITY_DN46720_c0_g1_i16|m.29980
MFLILSLVLVIYSLIYYYFRHKFSYWKRRGVETYPARFPFGHLGEFYLNKKSYIEIFTQYYDQSKSE
FCGIYQGIDPLLIVKNLEDIKYVLVKNFNCFMNRGVHTDFNSNVVSATLFRIEGDQWRNLRHKLTP
AFTSGKLKAMFETINQCCNGLDYALNKAMDPKTGEINVKMLAQRYTVDVISSVGFGVEANCLND
NYASPFFKLSQEILDRTALRTLLIKFQFFGPSYAKILYKFFSDRSKENQQSFFNMINSIVENRQKNKI
MRKDFLQLLIELKNHGTIDSSNDSIDNPNVFDKKDSLNFEFDMKTFVSQVFIFYFAGFDTSALTLT
MMLYYLAENQDLQKRLRKDINDCLRKHNNKLTYDGVMEMEYLEMLLSETLRLRPVLGHLFRVC
NKECYLPSSNLKVDVGTNICIPVYNIQRDEKYFPNPNKFDPERFS

>ON646346 CYP4422A2 [379 AA] TRINITY_DN46720_c0_g1_i7|m.29973
MFETINQCGNGLEYALNKAMDPKTGDINVQMLVQRYTVDVISSVGFGVEANSLKDNYASPIFNLS
QEIVVRKSLHSFLIKLAFFGPSYAKILYNFFSEGSKETRQSFLSMVNNIVESREKNKITRKDFLQLLI
ELKNHGKIDSSNDSLDNADVFDKKDSLNFEFSMKTFVSQVFVFYFAGFDTSALTLTMMLYYLAE
NQNIQKRLRKEIDECLKKHNNELTYDGVMEMEYLEMVLSETLRLRPVLGHLVRVCNKECYLPSS
NLKIDVGTTIGIPIYNIQRDEKYFPNPNKFDPERFSKENISKIPNFAYLPFGDGPRFCIGLRLGKLQTK
MGLISILRNHTVHLSDKSKISNYAPENVILKPKEPVILSFQKFISTDSE

>ON646347 CYP4422A2 [521 AA] TRINITY_DN46720_c0_g1_i8|m.29974
MFLILSLVLVIYSLIYYYFRHKFSYWKRRGVETYPARFPFGHLGEFYLNKKSYIEIFTQYYDQSKSE
FCGIYQGIDPLLIVKNLEDIKYVLVKNFNCFMNRGIHTDVNNNFLSEHLFRIEDEQWKNLRNKLSP
AFTSGKLKAMFETINQCGNGLEYALNKAMDPKTGDINVQMLVQRYTVDVISSVGFGVEANSLKD
NYASPIFNLSQEIVVRKSLHSFLIKLAFFGPSYAKILYNFFSEGSKETRQSFLSMVNNIVESREKNKIT
RKDFLQLLIELKNHGKIDSSNDSLDNADVFDKKDSLNFEFSMKTFVSQVFVFYFAGFDTSALTLTM
MLYYLAENQNIQKRLRKEIDECLKKHNNELTYDGVMEMEYLEMVLSETLRLRPVLGHLVRVCN
KECYLPSSNLKIDVGTNICIPVYNIQRDEKYFPNPNKFDPERFSKENILKIPNFAYLPFGDGPRFCIGL
RLGKLQTKMGLISILRNHTVHLSDKSKISNYAPENVILKPKEPVILSFQKFISTDSE

>ON646348 CYP4422A2 [521 AA] TRINITY_DN46720_c0_g1_i14|m.29979
MFLILSLVLVIYSLIYYYFRHKFSYWKRRGVETYPARFPFGHLGEFYLNKKSYIEIFTQYYDQSKSE
FCGIYQGIDPLLIVKNLEDIKYVLVKNFNCFMNRGIHTDVNNNFLSEHLFRIEDEQWKNLRNKLSP
AFTSGKLKAMFETINQCGNGLEYALNKAMDPKTGDINVQMLVQRYTVDVISSVGFGVEANSLKD
NYASPIFNLSQEIVVRKSLHSFLIKLAFFGPSYAKILYNFFSEGSKETRQSFLSMVNNIVESREKNKIT
RKDFLQLLIELKNHGKIDSSNDSLDNADVFDKKDSLNFEFSMKTFVSQVFVFYFAGFDTSALTLTM
MLYYLAENQNIQKRLRKEIDECLKKHNNELTYDGVMEMEYLEMVLSETLRLRPVLGHLVRVCN
KECYLPSSNLKIDVGTNICIPVYNIQRDEKYFPNPNKFDPERFSKENILKIPNFAYLPFGDGPRFCIGL
RLGKLQTKMGLIAILRNHTVHLSDKTKISNYAPEYVILKPKEHVILSFRKYISTEKE

>ON646349 CYP4416B2 [504 AA] TRINITY_DN46945_c0_g1_i1|m.30581
MFLIILVFLILFLSYFSNYLRVFYYVSKLPGPTAYPIIGNALLILGTNEDALSKVLKLFKDYSPFFRA
WLGPVPIVCTSNPDFVQRLLTGKYSMEKSFLMKYIGKEVVGNSIIISEVPKWKHNRNIIAQGFTPAI
LQSYFEIFLEKSLMLCDNLDVKLNDVKSFDIFQYLSQTSLDTICASTMGVNSNDENNKAAFYSAIE
NVLHLSVERSRYFWNIPKFIYCWSKSYKKHNESMKTIQEFANNVILQKKNYCKSYNNLDICTDRS
KNEFKKPLLEFLIDLQATNLDFTEKDLGDEANLVIIAGYETSATALSSVLLNLAVYKDVQNKVYEE
LFQVLGSDLNRTINFDDLPKLTYMEMVMKESMRLFPIAPFIIRELTDDIQLGDCLIPRGVSCAVSIY
ALHCDEKIWENPLKFDPERFTPENSIGRHPYAYIPFSGGPRNCIGMRYAWMFMKTVLATLLSRYEF
HTDLNTSEFKFEFAVTMKLIGGYQIQITPRKNDFLDHSR

>ON646350 CYP4416E1 [509 AA] TRINITY_DN46945_c0_g3_i2|m.30583

MLLEYIKNYLLVILLSLIVSLILCSYSKYLRIFYYTTKLPGPTALPIIGNILMLVGNHEEITTIVLNKV
QEYSPIFRAWLGPVPLIFTVKPEYIQEILNHGLEKAWIMKQAGYQLSGDSIMSSKVSKWKRNRRIV
VRGFSPILLKSYFKVFVEKNVILIENLNKKLNDDKPFDIFEYISKTTMDAVCGSTLGYDVNAQNSN
SSYYNAVQNLFFLMFERIFHPWKLPDIYYKFTEDGKQSEKSRDVILGLASKIITERRKIQYENAMQ
NHTLEIQDIKKNVFNKPLLDYLIDITENNQEFDNEQLRDEINIVILGGYETSANALSYVLLNLAAHK
DIQERVCKELFEVLDGDLNRPIEIDDLPKLKYMDMVLKESMRLYSTVPIIAREITKDIQMGDFFIPK
GSTCVIPILSLHRDHNLWKDPLKFDPERFTPENMKGRHPYAYIPFGGGPRNCIGIRYAWMFMKTLL
ATLLCRYEFHTDLNLNELKMQMEVSLKLVGGHQVRITSRIKN

>ON646351 CYP6YJ1 [516 AA] TRINITY_DN47041_c1_g2_i1|m.31304
MIKNITIIIEKYACVTLLVVTSRMFPITPLLLLLLILLAYYYFSYKFTYWKRKGVQSFPVTIPSGNILQ
ILVCRKSYTSVFTEIYKKINGAYAGIYFGLFPVLFVKDPEIIKDVLIKKFDHFQNRGVHFDEKHEPLS
AHLFRIEGEKWKTMRRKLTPAFSSGKLKWMTDIIFDCGNNLRSLLNDVYDGDNINFSIICHKYATD
VIASVGFGYEANSLKDDQSVFYHFASDIKSLNPLKKFVQQCVLFVPELLRLIPMRQQSKESEVFFL
KTIDETVKYREENNKTRNDFMQLLINMKNQENEITMRELAAQAYVFYIAGLETSNHTLSYTLYYL
AKYPEIQAKLLEEIDATFDTDEKFTYENINGMTYLEMVLLESLRLRPTLSMLNRVCNKPYTIPNTNI
TIDVGTKILISIIDLQTDPKYFPNPDKFDPERFSPANKSKIPSYCYMPFGEGPRKCIGERQGKFQTKIG
LISILRDHSVRICSYTKEPSEWLPGALLTVPKDPIILKLVKRKKSV

>ON646352 CYP9GK4 [350 AA] TRINITY_DN47405_c0_g2_i1|m.33522
RFTNDVIANTAFGVKCNSFQDKNNEFYLMGRAAIKFKAWRIILLSLIPAVLNKVLKFTAIPIYVCD
YFRNLVKHTTTIRKLQNIIRKDMLHLLMEAQKSTLKYENYIEYKEFAAVDESINSYGKARTLDDE
DVAAQAFIFFLTSFESTSTAMCFVAYELALNPDIQQRCYDEIHEILELTDGKLSYDAVQKIKYLDM
VISETLRKWPPAVATDRVCTKSYTLNVDGKDIFLEKGTNIEIPIYGIHHDPQFYPNPDKFDPERFSD
ENRHNIDPMTYLPFGVGPRNCIGSRFALMEIKVAMFHLIKNFEIILTNKSTVPPKLTTSTSCLGIVGG
FNVGLRPRPIDHTSTYLI

>ON646353 CYP9GK4 [364 AA] TRINITY_DN47405_c0_g2_i6|m.33525
EIKKLGGDSKHIELELKDSFTRFTNDVIANTAFGVKCNSFQDKNNEFYLMGRAAIKFKAWRIILLS
LIPAVLNKVLKFTAIPIYVCDYFRNLVKHTTTIRKLQNIIRKDMLHLLMEAQKSTLKYENYIEYKEF
AAVDESINSYGKARTLDDEDVAAQAFIFFLTSFESTSTAMCFVAYELALNPDIQQRCYDEIHEILEL
TDGKLSYDAVQKIKYLDMVISETLRKWPPAVATDRVCTKSYTLNVDGKDIFLEKGTNIEIPIYGIH
HDPQFYPNPDKFDPERFSDENRHSIDPMTYLPFGVGPRNCIGSRFALMEVKVAMFHLIKNFEIILTN
KSTVPPKLSTSTSAMVIEGGFNVGLKPRLVI

>ON646354 CYP9GK4 [404 AA] TRINITY_DN50479_c1_g2_i2|m.50193
MFFILLCLLGVLVLVFLYREKHYSSNYWENKGIKSFKALWLTDKVIRLVFQRVSLAETILSYYNAV
PNEKCVGFVAFNNATLIIRDTELIKQITVKDFDYFTDHPKTFFVEGVDGLLDKNLFALDGDTWRE
MRATLSPAFTGAKIRGMFQVVNECGDQMAAFYLNEIKKLGGDSKHIELELKDSFTRFTNDVIANT
AFGVKCNSFQDKNNEFYLMGRAAIKFKAWRIILLSLIPAVLNKVLKFTAIPIYVCDYFRNLVKHTT
TIRKLQNIIRKDMLHLLMEAQKSTLKYENYIEYKEFAAVDESINSYGKARTLDDEDVAAQAFIFFL
TSFESTSTAMCFVAYELALNPDIQQRCYDEIHEILELTDGKLSYDAVQKIKYLDMVISETLRKWPP
AVATDRVCTK

>ON646355 CYP4416D1 [515 AA] TRINITY_DN47476_c1_g3_i1|m.33831
MILMIASSFTINYVLLVTLTVLIGLLTFVYFSNYFRICYFVLKIRGPRAYPIIGNGLLFRGSLEEMSKV
VFETAIEYGPLTRLWMGPIPLLMTNEPRIIQEILTNANALEKAWLMKVMTRTMVGNAIIISEVPQW
KRMRSIITRAFTTSILKPYSNIFVDKMDILIEILDKELNKENTFDISKILSKAALDSVYASSMGVNMN
VQKGDSRFVDAMHRSCDLMIGRGLNPLKYPETIYRFTQDYKELMQCRDVVYSLTNKVISEKRNN
YKNGINNNQKDLKAVNTNAFKKPLLDYLIDFTENNPDFTDEQLADEINFITLEAMDTTAKQLSWV
LLIMAIHKDEQKKVYDELFEVLSNSNPDKIVFDDLSSLKYMEMVIKEAMRLFPVAPLIGRELTKDI
HFENYTIPKGTNIAIPIFSVHRNEDYWENPLKFDPERFSSENIKKHHPYAFLAFSAGPRNCIGMRYA
WLFMKIALACLLSRYEFHTSIKDMSDIRYQLELSLNVIGGHQVRITPRQK

>ON646356 CYP6YH6 [454 AA] TRINITY_DN47883_c2_g1_i1|m.36192
MRLITSSLSFDLLTLLLIGIILFYNYLKSKYNYWKERNVPYEEPNLIFGNFYDAFTFKRQITSFFHDQ
YRKTNDKVFGLYVFQRPFLLLRDPELIKHVLVKDFNNFIPRSNSKSSNDPIGNKNLFSLKNVHHWR

FMRSKMSPFFSSGKMRNVFGLLDTIGDQLTEHIKKHLNKTIDIKDVSQKYTIDVIVSAAYGIEANSF
TNEKAAFSSIGKVIFTYSLKRSVELISYFFAPLLVKLFNFKFFGDEATQFFRSVFWAAIKERELNYV
KRPDLIETLIALKNKGTLEDSPAVGDLGTVNNSKKLSSNKDTIKLEGDILVAQAAVFFAAGFETSSL
AISFTLYFLSYNPEIQIKLRKEIQTVIVRNGGKFTYDCLQDMKFLDACIKETLRMYPTLGFLDREAI
DTYTFPGTNITIDKGTAVVISLEGLHRDPQYFENPDVYDPERFNENNKINIV

>ON646357 CYP6YH7 [421 AA] TRINITY_DN47883_c3_g2_i1|m.36193
KDFNNFIPRTSAPTYKDDPLGEYNLFSMKSTHDWRFIRSKLSPIFSSGKLRNMFPLVNENGENLNG
YLKNHVFETLEGKSVCQNYTTDAIVSTVMGLSVNSFKDEETEFDRISKGLFTVTLRRATELMFFFF
VPFLSRLLRLKVFSEMGSQFYRNLFWSAVKMRQDNNIKRPDLIDALITLKNYGTIEDADSKEQKK
EVVSNPSQLKLDGDVLVAQIALFYAAGLNTSSAAMSFTCYSLSYNPEIQIKLRKEIQTVLARNGGK
LTYESVFEMKYLDCCFKETLRLYPSLGYLDREALDTYTFPGTNLTIEKGTPVIISLAGMQKDPQYF
KNPDVYDPERFNDENKDKIPPYVFMPFGDGPRNCIGARLGQLITKVGLINILKDYEVVTVASKPLL
ELNPKAFFTEAKGGISLQFKPGTVYV

>ON646358 CYP6YH7 [380 AA] TRINITY_DN47883_c3_g2_i2|m.36198
KDFNNFIPRTSAPTYKDDPLGEYNLFSMKSTHDWRFIRSKLSPIFSSGKLRNMFPLVNENGENLNG
YLKNHVFETLEGKSVCQNYTTDAIVSTVMGLSVNSFKDEETEFDRISKGLFTVTLRRATELMFFFF
VPFLSRLLRLKVFSEMGSQFYRNLFWSAVKMRQDNNIKRPDLIDALITLKNYGTIEDADSKEQKK
EVVSNPSQLKLDGDVLVAQIALFYAAGLNTSSAAMSFTCYSLSYNPEIQIKLRKEIQTVLARNGGK
LTYESVFEMKYLDCCFKETLRLYPSLGYLDREALDTYTFPGTNLTIEKGTPVIISLAGMQKDPQYF
KNPDVYDPERFNDENKDKIPPYVFMPFGDGPRNCIGELHKFVIIHLSPLAI

>ON646359 CYP6YH8 [389 AA] TRINITY_DN47883_c3_g5_i3|m.36196
MALVTSSWFTDILTLLIVTLSIVYYFLTSTYNYWKNKNIPYEKPTLIFGNFYNAVTFQQNITDYFAD
QYRKTKEKFFGLYIFRRPYLLIRDPELAKHVLIKDFNNFVPRTTAPTHKDDPMGQYNLFSMKNNN
DWRFIRSKLSPIFSSGKLRNTFPLINEIGENLNGYLKNHVFETLEGKSVCRKYTSDVIVSTVFGISTN
SFTDEETEFEKLSKTVFTFTLRRAYELMFFFFVPTVSRLFRLKVFSEEGTEFFRNIFWSAVKMRDEH
NIKRPDLIDALITLKNYGTIEDPDNKENSKEVVSNPSQLKLDGDVLVAQIALFYAAGLDTSSNAMS
FTCYSLSYNPEIQIKLRKEIQSVLARNGGQLTYDSLSEMKFLDCCIRGINTIQIMS

>ON646360 CYP4421A1 [498 AA] TRINITY_DN47910_c3_g2_i3|m.36888
MITIIILLLILIAISIRYYKENFNYDYWKIRNVKFLKPLPIFGNYLPAFLMQMSTGKLLEKIHRHYENEP
YVGFYIFNKPYLLIKDPKIIQSILVKDFTKFNNRSTASVIENDFIGTYSLFALKDTEWKVLRSKVLPF
FSTKQMKLMFELITDVGEHLNEEIKQIYNKKTNIFETKDTAGKFASDIIAACVYGIPPPNEFLQISKSI
FEPSVIRNFELNSFFFMKIFASIFKFTFIGRKATNYLRMLFWNIINTREMEVQHNSKMKQDNFLNFLI
ELRKNKEISLTHDKLVAQAIIFFTAGIEGPATAISQGLYHIALNSNIQRRLRKEIKQALADNNNELTF
ENIKELPYLDACVRESFRMAIALPFLDRQASEDYKVPGTDLIIEKGMGIFISLYGMHYDPKYFPNPN
KFDPERFLTNKSKLNQFAYLPFGAGPRNCIGARFANIQVKIGLINVLKDFQVEAAKKIDEPFKAHP
RGFFYTLANGYDLRFKKDDDIND

>ON646361 CYP4421A1 [446 AA] TRINITY_DN47910_c3_g3_i5|m.36892
MITIIILLLILIAISIRYYKENFNYDYWKIRNVKFLKPLPIFGNYLPAFLMQMSTGKLLEKIHRHYENEP
YVGFYIFNKPYLLIKDPKIIQSILVKDFTKFNNRSTASVIENDFIGTYSLFALKDTEWKVLRSKVLPF
FSTKQMKLMFELITDVGEHLNEEIKQIYNKKTNIFETKDTAGKFASDIIAACVYGIPPPNEFLQISKSI
FEPSVIRNFELNSFFFMKIFASIFKFTFIGRKATNYLRMLFWNIINTREMEVQHNSKMKQDNFLNFLI
ELRKNKEISLTHDKLVAQAIIFFTAGIEGPATAISQGLYHIALNSNIQRRLRKEIKQALADNNNELTF
ENIKELPYLDACVRESFRMAIALPFLDRQASEDYKVPGTDLIIEKGMGIFISLYGMHYDPKYFPNPN
KFDPERFLTNKSKLNQFAYLPFGAGPRNCIGKYIICK

>ON646362 CYP4XF3 [470 AA] TRINITY_DN48043_c3_g2_i1|m.37118
MDDDAMNIKQQEQQNDKQTTTTILINNNHEEEEVYTTINNNNNNTTTTISDNEQQQQPQQSINRN
TWSRTSLRRSPIHNNNGGGTGSGSIKRWGSFRTTNKRQLGSNALASELYRSSSFNCTSGSGGNVTT
RTTNCNLDDRMSTTSKDDLYGCSSMTTTYSSMSDHDQDIIDHGSLLEDDVLDLNHKVEQLQQQV
NILTSTTIEQQQHLNNNNINNNINNNNNNIIDDNSNVCLGSKKRIIFLDLLLQSTHLSGEPLTDKEIID
EVTNFMFAGHDTTSVQLSLLMYLLCEHPEIQNELYKEQCAILTDINGDPSFKQIQEMNYLERTIKES
QRILPCVISFSRKMTADIQLKTNNLLLPKGCTASIFVYDVHHNPKIYENPEKFDPDRFLPENIRGRHP

YAYIPFSAGPRNCLGQKFALLEMKTVMSNIIRNFKILPAYEDNGDKFKPIFRRYMLLTSLNGIQIRL
ESRR

>ON646363 CYP6YK1 [499 AA] TRINITY_DN48154_c0_g1_i1|m.37978
MIIFLSILLILISILYYYLMSNYDYFEKKNVPYDKPVLLFGNIRDRLLQKVCLHDFMQYYYMKYKN
NHPICGVYEGRNPRYLVLDFNLVKDIFIRDFDHFTDRSLFNFKQADILKDLLLMSSGPIWKPTRAK
LTPAFSSGKLKAMDVLIQRCGEQMSQHLLERKNPDEELEMKQFFGLFTLDVISNCAFGIESNPWKE
TDPELITAFRNFDSMTTLTRMRLLASLVLMPHYFRRFVTVWIFNKKTAYFIANIIRNTREYRLKNN
QRRNDFLQLLLDISAQEEEEKPEHRLMDVDHVISQSILFMIAGFETSSTLLGFAAYELALNPEVQNT
LRAEIKSVLEKHNNVCNYDAVQDMHYLEMVLLETLRKHSPVARIDRCCVKEYKIRGTDIVMEKG
MNVSVPVVGFHYDPTYFPNPEKFDPLRFQENRNPDGFLSFGIGPRNCIGKRFALISTKFAMVYLIK
DFELKATEKTEVPYTKRKIGILLYPQNGLHVKISKLNE

>ON646364 CYP6YK1 [374 AA] TRINITY_DN48154_c0_g1_i3|m.37980
MIIFLSILLILISILYYYLMSNYDYFEKKNVPYDKPVLLFGNIRDRLLQKVCLHDFMQYYYMKYKN
NHPICGVYEGRNPRYLVLDFNLVKDIFIRDFDHFTDRSLFNFKQADILKDLLLMSSGPIWKPTRAK
LTPAFSSGKLKAMDVLIQRCGEQMSQHLLERKNPDEELEMKQFFGLFTLDVISNCAFGIESNPWKE
TDPELITAFRNFDSMTTLTRMRLLASLVLMPHYFRRFVTVWIFNKKTAYFIANIIRNTREYRLKNN
QRRNDFLQLLLDISAQEEEEKPEHRLMDVDHVISQSILFMIAGFETSSTLLGFAAYELALNPEVQNT
LRAEIKSVLEKHNNVCNYDAVQDMHYLELHHNYKHCYVFLILI

>ON646365 CYP4418A1 [509 AA] TRINITY_DN48169_c1_g1_i10|m.38105
MFFDILLILITITLCFVIFYTWNYDYWKKRRIPYESPIFPFGSIKDALLLRKPLGSIIHELYYKYKDYK
YFGIFFGRHPVLFIRDPELFKMVTVKNFNDFQDNTASMSEKSDILGALNPFILKGERWKISRTHITP
AFTSLKIKQNMYPNMVDVSERLKMYITKNLDNFSNGIKIQNLARDFASEIFTLCGLAIKTDAFEDS
KNPFKIIAHEMFETESFTGILKFILMIVDPAVSLLLRTALMSKTAVEYFRKVIRDVRLQRKINNINTN
DYFSYLDKLQIDNADFSEDDLASHIVTLFIDGVETTASTIKLTMAEIAANPRVFKKLREEIDRIMVK
YDNKLTYDALRDMTYLDHCISEALRLHPLFMFFMRNCTAGSFQFPPYKDGVNRDVKIDKGFSVL
LPVYALQHDENLHANPETFYPERFSDEDTKVQSKANYFGFGDGPRICLGMKYAQLVTKIGIWAMI
SNFDIVLNEKSKYPIKNAGASFVFVPEDEPLLNFYKRGEIYSL

>ON646366 CYP4418A1 [366 AA] TRINITY_DN48169_c1_g1_i9|m.38103
MFFDILLILITITLCFVIFYTWNYDYWKKRRIPYESPIFPFGSIKDALLLRKPLGSIIHELYYKYKDYK
YFGIFFGRHPVLFIRDPELFKMVTVKNFNDFQDNTASMSEKSDILGALNPFILKGERWKISRTHITP
AFTSLKIKQNMYPNMVDVSERLKMYITKNLDNFSNGIKIQNLARDFASEIFTLCGLAIKTDAFEDS
KNPFKIIAHEMFETESFTGILKFILMIVDPAVSLLLRTALMSKTAVEYFRKVIRDVRLQRKINNINTN
DYFSYLDKLQIDNADFSEDDLASHIVTLFIDGVETTASTIKLTMAEIAANPRVFKKLREEIDRIMVK
YDNKLTYDALRDMTYLDHCISGILLFFHI

>ON646367 CYP4418A1 [366 AA] TRINITY_DN48169_c1_g1_i13|m.38111
MFFDILLILITITLCFVIFYTWNYDYWKKRRIPYESPIFPFGSIKDALLLRKPLGSIIHELYYKYKDYK
YFGIFFGRHPILFIRDPELFKMVTVKNFNDFQDNTASMSEKSDILGALNPFILKGERWKISRTHITPA
FTSLKIKQNMYPNMVDVSERLKMYITKNLDNFSNGIKIQNLARDFASEIFTLCGLAIKTDAFEDSK
NPFKIIAHEMFETESFTGILKFILMIVDPAVSLLLRTALMSKTAVEYFRKVIRDVRLQRKINNINTND
YFSYLDKLQIDNADFSEDDLASHIVTLFIDGVETTASTIKLTMAEIAANPRVFKKLREEIDRIMVKY
DNKLTYDALRDMTYLDHCISGILLFFHI

>ON646368 CYP4418A1 [509 AA] TRINITY_DN48169_c1_g1_i17|m.38118
MFFDILLILITITLCFVIFYTWNYDYWKKRRIPYESPIFPFGSIKDALLLRKPLGSIIHELYYKYKDYK
YFGIFFGRHPVLFIRDPELFKMVTVKNFNDFQDNTASMSEKSDILGALNPFILKGERWKISRTHITP
AFTSLKIKQNMYPNMVDVSERLKMYITKNLDNFSNGIKIQNLARDFASEIFTLCGLAIKTDAFEDS
KNPFKIIAHEMFETESFTGILKFILMIVDPAVSLLLRTALMSKTAVEYFRKVIRDVRLQRKINNINTN
DYFSYLDKLQIDNADFSEDDLASHIVTLFIDGVETTASTIKLTMAEIAANPRVFKKLREEIDRIMVK
YDNKLTYDALRDMTYLDHCISEALRLHPLFMFFMRNCTAGSFQFPPYKDGVNRDVKIDKGFSVL
LPVYALQHDENLHANPETFYPERFSDEDTKVQSKAIYYGFGDGPRICLGMKYAQLVTKIGIWAMI
SNFDIVLNEKSKYPIKNAGPSFIFVPEDEPLLNFYKRGEIYSR

>ON646369 CYP4418A1 [509 AA] TRINITY_DN48169_c1_g1_i18|m.38120
MFFDILLILITITLCFVIFYTWNYDYWKKRRIPYESPIFPFGSIKDALLLRKPLGSIIHELYYKYKDYK
YFGIFFGRHPILFIRDPELFKMVTVKNFNDFQDNTASMSEKSDILGALNPFILKGERWKISRTHITPA
FTSLKIKQNMYPNMVDVSERLKMYITKNLDNFSNGIKIQNLARDFASEIFTLCGLAIKTDAFEDSK
NPFKIIAHEMFETESFTGILKFILMIVDPAVSLLLRTALMSKTAVEYFRKVIRDVRLQRKINNINTND
YFSYLDKLQIDNADFSEDDLASHIVTLFIDGVETTASTIKLTMAEIAANPRVFKKLREEIDRIMVKY
DNKLTYDALRDMTYLDHCISEALRLHPLFMFFMRNCTAGSFQFPPYKDGVNRDVKIDKGFSVLLP
VYALQHDENLHANPETFYPERFSDEDTKVQSKAIYYGFGDGPRICLGMKYAQLVTKIGIWAMISN
FDIVLNEKSKYPIKNAGPSFIFVPEDEPLLNFYKRGEIYSR

>ON646370 CYP4418A1 [419 AA] TRINITY_DN48169_c1_g1_i22|m.38125
MVTVKNFNDFQDNTASMSEKSDILGALNPFILKGERWKISRTHITPAFTSLKIKQNMYPNMVDVS
ERLKMYITKNLDNFSNGIKIQNLARDFASEIFTLCGLAIKTDAFEDSKNPFKIIAHEMFETESFTGIL
KFILMIVDPAVSLLLRTALMSKTAVEYFRKVIRDVRLQRKINNINTNDYFSYLDKLQIDNADFSED
DLASHIVTLFIDGVETTASTIKLTMAEIAANPRVFKKLREEIDRIMVKYDNKLTYDALRDMTYLDH
CISEALRLHPLFMFFMRNCTAGSFQFPPYKDGVNRDVKIDKGFSVLLPVYALQHDENLHANPETF
YPERFSDEDTKVQSKAIYYGFGDGPRICLGMKYAQLVTKIGIWAMISNFDIVLNEKSKYPIKNAGP
SFIFVPEDEPLLNFYKRGEIYSR

>ON646371 CYP4XK1 [519 AA] TRINITY_DN48411_c1_g2_i4|m.39396
MILIILEILILLASAIIAIYSYNNVRKHLNGFYHKAELIPGPQGHFLLGSIREIVGTDGGIYNWLVQAR
KKYGPVLKFWALDSLHVMLADPKDVELILKSQKTLTKSYGYNFMRPLMGEGLVTSAGEHWREH
RKIITPTFHFNILEKFVETFNENAEILIKRLSDVQKENPTLDIHTFLTLCALDIICETAMGTKINAQQS
GGLNDYVQAVNLAAYMVVHRYYNLLHRFRFISKFTPLGKQYERCLQLLLNRTDEIIRTRKAELKR
EKENKSVEVKKSEEDVGRKRRMAFLDLLLQTTHTNGSPLSDTEIRDEVNTFMVAGHETTSIGTST
CLYLLSINPEVQEKVVEELNDIFGNDTRAPSYEDLQKLSYLERTIKESQRLMPSIAEFQRYIKEDFPI
NNYVIPGGSTIGVMVYAMHIDPEIFPDPMKFDPDRFLPENSRNRHPFAYIPFSGGYRNCIGQKFAM
LEMKAVISSVLRNFKIEAAIDEKTGKVFKPIIEERVTTGSKNGIYVKLLPRQKS

>ON646372 CYP4XF1 [511 AA] TRINITY_DN48411_c1_g3_i5|m.39402
MIEIIIGALVAIVVALYVYFELPLKRIKRLTSGIPGPKGLPIVGILFDVRKEKAGTFGFLWNNTQKYG
PIFKIPSVGRLAINMTNPEDIEVLLKNNEILDKPYSTTKFFKPWLGNGLVFSAGPYWKQQRKLITPT
FHFNILEKFFEVFNEQGDILIKLLKEKADGKTNIDIFPYMQSFALDVICETAMGTKIHAQTSARSAY
VLAVQDMERIIVERFISVIQQIDFLFPFTENGQTQRKALQILKESTDKLIRKRRDDLIKESENKSDEN
QDDSFGTKKSLAFLDLMLQARNMDGTPLTNEQISDEVSTFTFAGHDTTSTQLAFLFYLLSKNPHIQ
EEIYKEQCDILMGEKRDPTFQEVQEMHYLERTIKEAQRLLPSVPLFSRQVTKDLQLKTNNYVAPE
GSILTVFTFGLHRNPNIYPDPEKFDPDRFLPENLRERNPYSFVPFSAGPRNCIGQKFAMLEMKTSSS
KILRNFKILPAFEADGTPYEPDLRAYIVLTAVNGIQVRLVSRT

>ON646373 CYP4XF1 [405 AA] TRINITY_DN50017_c0_g1_i2|m.47968
MIEIIIGALVAIVVALYVYFELPLKRIKRLTSGIPGPKGLPIVGILFDVRKEKAGTFGFLWNNTQKYG
PIFKIPSVGRLAINMTNPEDIEVLLKNNEILDKPYSTTKFFKPWLGNGLVFSAGPYWKQQRKLITPT
FHFNILEKFFEVFNEQGDILIKLLKEKADGKTNIDIFPYMQSFALDVICETAMGTKIHAQTSARSAY
VLAVQDMERIIVERFISVIQQIDFLFPFTENGQTQRKALQILKESTDKLIRKRRDDLIKESENKSDEN
QDDSFGTKKRLAFLDLMLQARNMDGTPLTNEQISDEVSTFTFAGHDTTSTQLAFLFYLLSKNPHIQ
EEIYKEQCDILMGEKRDPTFQEVQEMHYLERTIKEAQRLLPSVPLFSRQVTKDLQLKTSNYQIRITI
QR

>ON646374 CYP4XF2 [367 AA] TRINITY_DN48411_c1_g3_i6|m.39403
IEIIIGALVAIVVALYVYFELPLKRIKRLTSGIPGPKGLPIVGILFDVRKEKAGTFGFLWNNTQKYGPI
FKIPSVGRLAINMTNPEDIEVLLKNNEILDKPYSTTKFFKPWLGNGLVFSAGPYWKQQRKLITPTFH
FNILEKFFEVFNEQGDILIKLLKEKADGKTNIDIFPYMQSFALDVICETAMGTKVHAQTKPRSAYVL
AVHNMARIIVERVLSAIQQIDFLFAFTENGRTQRKALKVLKESTDKLIRKRRNDLIKESENKSNESQ
ADTVGTKKRLAFLDLMLHARNMDGTPLTNEQISDEVSTFTAAGHDTTATQLALLFYLLSKNQHIQ
EEIYKEQCDILMGENRDPTFQEVQEMHYLERT

>ON646375 CYP4XFfragment1 [353 AA] TRINITY_DN48411_c1_g3_i15|m.39409
MIEIFIGLVGVILVVLYLRLELPLRRIKKLTSTIPGPKGLPLVGILFDITKDKDALGYLWKCTQTYGH
IFKIPIVGRLAINMSNPEDIEVLLKNNEILEKPYTTAKFFRPWLGNGLLLSRGSYWKHQRKLITPTFH
FNILEKFYEVFNEQGQILIEILKEKADGKTNIDIFPYMQSFALDVICETAMGTKVHAQTKPRSAYVL
AVHNMARIIVERVLSAIQQIDFLFAFTENGRTQRKALKVLKESTDKLIRKRRNDLIKESENKSNESQ
ADTVGTKKRLAFLDLMLHARNMDGTPLTNEQISDEVSTFTAAGHDTTATQLALLFYLLSKNQHIQ
EEIYKEQCDILMGEKQES

>ON646376 CYP12AS1 [518 AA] TRINITY_DN48442_c4_g1_i3|m.39562
MLCSRFKITPNINKNYNILRCLLSTTNVAVEWDNAQPFETLPGPKPLPILGNAPRFLPPFGEYSGKN
FLQIYQQLQKQYGNIMVLRGLPGREPIVVTYSVNDIETMYRNEGPRPYRPGLDSLRYYRKHVRND
VHKILGLALSQGDDWWDMRSKVNPVMMKTQSIRRYTKTVSSIADDLIKKIDILLSESDKSELPDDF
LNELFRWSLETVCAIALDTRLGCLDPNMSHDSEAQKMIDASVEMFECIYELDILPSVWKFVSTPTL
KKSIKVNDFITSYVMKHLDAAEERMKNKPSNSDHEPSILERLIVIDKNVASAMVNDAMIAGIETTS
KTVATVMYFLATNPNAQDKLREEINTIWPDSSTDITSKQIDQMLYLRACIKEAQRIAPVTVGTVRK
LDVDVVLSGYQIPKGTDILSPNIVLCLSDKHFPQPEKYLPERWLRSSPSELSARNTNMFVYLPFGFG
PRTCIGKRLATHEVEVLIAKLFKKYKFQWNYGPMRYTTKLLYEVADPLRFELHPV

>ON646377 CYP18A1 [533 AA] TRINITY_DN48592_c0_g1_i2|m.39903
MFLYTIQYFLNRLWKFENDTFSMLAVFLLALCVVRFIQFYMELKALPPGPWGVPILGLLPTLSGIR
APYLHYLELKQKYGPLFSMKVGSQLHVIMTDYKLIRDTFRKDEFSGRPQGPFHDLLSGYGIINSEG
KLWKDQRRFLHNRFRNFGVTYLGSGKNQIEKRIMGEVVEFLSVLKSFEGAPTDPSPILSVCISNVIC
EMMMSVRFTHNDPKFLRFMGLTQEGLQLWGSMNYATCIPVLQYFPKFRVILKKLGENRDEMSAF
FQEQLEEHRRTFDPENIRDLIDTYLLEIEKAKENGTDSNLFEGRNHDRQIRQVIADLFTAGMETIKN
TLQFAILYMIHHPESLRAVQDELDQIVGRNRLPKLEDLPYLPTTESTLYEVMRISSIVPLGTEHSPIR
DVTVNGYRIPQNTHVHPFLHAVHMDPNLWDEPERFNPSRFINSAGNCVKPEYFLPFGVGRRMCL
GDVLARMELFLFFSSMLHTFNISVPEGEKLPSLKPNSGITLFPEAFKVCFKPRSLNTDTVHPRQEKN
IGVH

>ON646378 CYP4XG1 [516 AA] TRINITY_DN48583_c1_g1_i1|m.40225
MIDNKLNDINKNDNSLSTYFLLKYTLLTIIVISVIKFYWDRRRLYICALKFSGPTALPLLGNALLFA
VQPEDILSRIYEIVCKYSSPCRLWFGHKLMTIIYAPEDLEVVLSDTVNIKKDHTYSFIEPFLGQGLIS
NSGSLWKTHRRLIIPTFTSKTLRYYVESFNTHAKTLVEKLQPIAERKETVNIIDPLNLCTVDIVLSTIL
GIEAGAQDHKIDEFVEHVELGYKMVAERMFKGWLHLDAIFRLSKCYRNTMKAQKVIHDFAKKQ
LTTLKAEFNQKYQDGLIDEHNVRTTLEHLLLINKKGDDFFTDVELRDETYTLFTASQDTVATECAF
ILLMLAMHPEIQEKVYKEILEVSGETKDYYTEWETYPEFKYLEMVIKESLRLFPIGPTIMREVIQDI
KLKNDIVIPKGAGVMVVMYRTHRLPEYWTDPEKFIPERFLPELCEKRHPFAYVPFSFGPRSCIAPR
YAMAAMKTIVTHVIRSYKLTCEDKFEDLQLCSDISTRSRNGYKMKLELRK

>ON646379 CYP4XG1 [420 AA] TRINITY_DN48583_c1_g1_i3|m.40227
MTIIYAPEDLEVVLSDTVNIKKDHTYSFIEPFLGQGLISNSGSLWKTHRRLIIPTFTSKTLRYYVESF
NTHAKTLVEKLQPIAERKETVNIIDPLNLCTVDIVLSTILGIEAGAQDHKIDEFVEHVELGYKMVAE
RMFKGWLHLDAIFRLSKCYRNTMKAQKVIHDFAKKQLTTLKAEFNQKYQDGLIDEHNVRTTLEH
LLLINKKGDDFFTDVELRDETYTLFTASQDTVATECAFILLMLAMHPEIQEKVYKEILEVSGETKD
YYTEWETYPEFKYLEMVIKESLRLFPIGPTIMREVIQDIKLKNDIVIPKGAGVMVVMYRTHRLPEY
WTDPEKFIPERFLPELCEKRHPFAYVPFSFGPRSCIAPRYAMAAMKTIVTHVIRSYKLTCEDKFEDL
QLCSDISTRSRNGYKMKLELRK

>ON646380 CYP4XG1 [506 AA] TRINITY_DN48583_c1_g1_i4|m.40228
MIDNKLNDINKNDNSLSTYFLLKYTLLTIIVISVIKFYWDRRRLYICALKFSGPTALPLLGNALLFA
VQPEDILSRIYEIVCKYSSPCRLWFGHKLMTIIYAPEDLEVVLSDTVNIKKDHTYSFIEPFLGQGLIS
NSGSLWKTHRRLIIPTFTSKTLRYYVESFNTHAKTLVEKLQPIAERKETVNIIDPLNLCTVDIVLSTIL
GIEAGAQDHKIDEFVEHVELGYKMVAERMFKGWLHLDAIFRLSKCYRNTMKAQKVIHDFAKKQ
LTTLKAEFNQKYQDGLIDEHNVRTTLEHLLLINKKGDDFFTDVELRDETYTLFTASQDTVATECAF
ILLMLAMHPEIQEKVYKEILEVSGETKDYYTEWETYPEFKYLEMVIKESLRLFPIGPTIMREVIQDI

KLKNDIVIPKGAGVMVVMYRTHRLPEYWTDPEKFIPERFLPELCEKRHPFAYVPFSFGPRSCIGTLF
LKNRMQKFYLIFYFYLLVFSSEICYGCYENNRNPRHQKL

>ON646381 CYP4C164partial [357 AA] TRINITY_DN48799_c1_g2_i1|m.41074
KADGAEFDLYPYITRCALDIICETAMGVKIDAQRHIEANLGDKPEALDSSDSEYVKAIYDFSDLAL
RRGFVIWYHINFIWNNSSSGRRYNKCVEILHGFTNKVILERKEALKAKKPIVPKEENEADSIGTKK
RMAFLDLLLEASLDGHHLTTEEIREEVDTFMFEGHDTTTAAICWSLFLLGNHMDVQQKCHEELDS
IFQDTDRDITRQDLADMKYLEMVIKETLRMYPSVPIISRELGEDIVLDGFTVPKKTFAVMHLHETH
RNEAHFKNPNTFNPENFNKDNCAKRHPYAYIPFSAGLRNCIGQKFAILEEKTVLASILRKFRIESIES
VEECKKVGALILRPLNGVQVFLHRRH

>ON646382 CYP9GN5 [512 AA] TRINITY_DN48771_c0_g2_i4|m.41099
MFAIIALIVILYLIHYAITNVYKYWESRGIPYYKPRNAYYDTYCTFLQKDNFSLEIAKAYNGFPDKQ
VYGSFLLRTPQLMVKDPDILRNICVKDFEHFTNHVDIFFKDDLLLGKSLFALKDKKWRDMRTILSP
IFTGSKLRGMVDLVSECSEQTMDYYAQLIKQKSDKTVKVDILDATSRFTNDVIATSAFGIQVNSLA
DKTNVFFETGQEVSKFPFWRFVVAAFPKFVKKIFNLSLFPSKIYEFFRNIVEESIRIRKTQNIVRYDLI
HLLLEAKKEQINKINEKSDNDQKLELSKNLLDIDDITAQSFIFFIAGFINVALTISFAGYELAIQPEIQ
DKLRKEIRELLQKNNGKLDYDILKKMKYLDMVVTEVLRKYPPAGMLDRVCTKAYTIKTATESID
LVPGMPINIPVMGLHYDPKYFPNPNKFDPERFNDENREKMNPYTYLPFGVGPRLCIASRFVLMEIK
IFLVHLINSFEVIATEKCPIPMEFETGTVLKPKHDEFGIAIKLIK

>ON646383 CYP9GN5 [512 AA] TRINITY_DN48771_c0_g2_i1|m.41097
MFAIIALIVILYLIHYAITNVYKYWESRGIPYYKPRNAYYDTYCTFLQKDNFSLEIAKAYNGFPDKQ
VYGSFLLRTPQLMVKDPDILRNICVKDFEHFTNHVDIFFKDDLLLGKSLFALKDKKWRDMRTILSP
IFTGSKLRGMVDLVSECSEQTMDYYAQLIKQKSDKTVKVDILDATSRFTNDVIATSAFGIQVNSLA
DKTNVFFETGQEVSKFPFWRFVVAAFPKFVKKIFNLSLFPSKIYEFFRNIVEESIRIRKTQNIVRYDLI
HLLLEAKKEQINKINEKSDNDQKLELSKNLLDIDDITAQSFIFFIAGFINVALTISFAGYELAIQPEIQ
DKLRKEIRELLQKNNGKLDYDILKKMKYLDMVVTEVLRKYPPAGMLDRVCTKAYTIKTATESID
LVPGMPINIPVMGLHYDPKYFPNPNKFDPERFNDENREKMNPYTYLPFGVGPRLCIASRFVLMEIK
IFLVHLINSFEVIATEKCPIPMEFETGTVLKPKHDEFGIAIKLIK

>ON646384 CYP9GN5 [458 AA] TRINITY_DN48771_c0_g2_i5|m.41100
MFAIIALIVILYLIHYAITNVYKYWESRGIPYYKPRNAYYDTYCTFLQKDNFSLEIAKAYNGFPDKQ
VYGSFLLRTPQLMVKDPDILRNICVKDFEHFTNHVDIFFKDDLLLGKSLFALKDKKWRDMRTILSP
IFTGSKLRGMVDLVSECSEQTMDYYAQLIKQKSDKTVKVDILDATSRFTNDVIATSAFGIQVNSLA
DKTNVFFETGQEVSKFPFWRFVVAAFPKFVKKIFNLSLFPSKIYEFFRNIVEESIRIRKTQNIVRYDLI
HLLLEAKKEQINKINEKSDNDQKLELSKNLLDIDDITAQSFIFFIAGFINVALTISFAGYELAIQPEIQ
DKLRKEIRELLQKNNGKLDYDILKKMKYLDMVVTEVLRKYPPAGMLDRVCTKAYTIKTATESID
LVPGMPINIPVMGLHYDPKYFPNPNKFDPERFNDENREKMNPYSYLPFGVGPRLCIG

>ON646385 CYP9GN5 [512 AA] TRINITY_DN48771_c0_g1_i1|m.41096
MFAIIALIVILYLIHYAITNVYKYWESRGIPYYKPRNAYYDTYCTFLQKDNFSLEIAKAYNGFPDKQ
VYGSFLLRTPQLMVKDPDILRNICVKDFEHFTNHVDIFFKDDLLLGKSLFALKDKKWRDMRTILSP
IFTGSKLRGMVDLVSECSEQTMDYYAQLIKQKSDKTVKVDILDATSRFTNDVIATSAFGIQVNSLA
DKTNVFFETGQEVSKFPFWRFVVAAFPKFVKKIFNLSLFPSKIYEFFRNIVEESIRIRKTQNIVRYDLI
HLLLEAKKEQINKINEKSDNDQKLELSKNLLDIDDITAQSFIFFIAGFINVALTISFAGYELAIQPEIQ
DKLRKEIRELLQKNNGKLDYDILKKMKYLDMVVTEVLRKYPPAGMLDRVCTKAYTIKTATESID
LVPGMPINIPVMGLHYDPKYFPNPNKFDPERFNDENREKMNPYTYLPFGVGPRLCIASRFVLMEIK
IFLVHLINSFEVIATEKCPIPMEFETGTVLKPKHDEFGIAIKLIK

>ON646386 CYP9GN5 [458 AA] TRINITY_DN48771_c0_g2_i3|m.41098
MFAIIALIVILYLIHYAITNVYKYWESRGIPYYKPRNAYYDTYCTFLQKDNFSLEIAKAYNGFPDKQ
VYGSFLLRTPQLMVKDPDILRNICVKDFEHFTNHVDIFFKDDLLLGKSLFALKDKKWRDMRTILSP
IFTGSKLRGMVDLVSECSEQTMDYYAQLIKQKSDKTVKVDILDATSRFTNDVIATSAFGIQVNSLA
DKTNVFFETGQEVSKFPFWRFVVAAFPKFVKKIFNLSLFPSKIYEFFRNIVEESIRIRKTQNIVRYDLI
HLLLEAKKEQINKINEKSDNDQKLELSKNLLDIDDITAQSFIFFIAGFINVALTISFAGYELAIQPEIQ

DKLRKEIRELLQKNNGKLDYDILKKMKYLDMVVTEVLRKYPPAGMLDRVCTKAYTIKTATESID
LVPGMPINIPVMGLHYDPKYFPNPNKFDPERFNDENREKMNPYSYLPFGVGPRLCIG

>ON646387 CYP9GK5 [522 AA] TRINITY_DN48962_c0_g2_i10|m.41746
MLVLSLFIILIAIIYFYFIRPLNYWKNLDVPFIPGYPIVGNFIWTSLKKQSFTDELNSLYNAFPNEKCV
GMFSFGTAPALLIRDPELLKQITIKDFNYFTDHTKSFFKDGFDTLIDRNLFTLSGNEWRDMRATLSP
AFTGSKMRGLFNMMNDCSEQFVSYYSNKVKNAKDSHLEVELKKSFTRFTNDIIASAVFGVKCDS
LNNEQNEFYLMGCKAAYFATWRILLSNFCPWILKFLNLGGVIPEECCKYFKNLVYETKAVREREN
IIRPDILHLLIQAQKGALKDDSVEENKGFVGTESKTVGNIKKLDDDDIAAQAFLFFLGGFETTSILM
CFTIFELALNPDIQERLLNEIQNTLSESNGEFSYDVVHKMKYLDMIVTETLRKWPPAPITDRVTTKN
YKIQVGKKLITLEPGFPITVPIIGFHRDPQYYPNPEKFDPERFNDENKKNIKPLTFCPFGAGPRLCIGA
RFAVMNAKIIIFHMIKNFEVILVKNSTVPPVLSSKSGNTILENGFHVGLKLRKL

>ON646388 CYP9GK5 [409 AA] TRINITY_DN48962_c0_g2_i4|m.41738
MLVLSLFIILIAIIYFYFIRPLNYWKNLDVPFIPGYPIVGNFIWTSLKKQSFTDELNSLYNAFPNEKCV
GMFSFGTAPALLIRDPELLKQITIKDFNYFTDHTKSFFKDGFDTLIDRNLFTLSGNEWRDMRATLSP
AFTGSKMRGLFNMMNDCSEQFVSYYSNKVKNAKDSHLEVELKKSFTRFTNDIIASAVFGVKCDS
LNNEQNEFYLMGCKAAYFATWRILLSNFCPWILKFLNLGGVIPEECCKYFKNLVYETKAVREREN
IIRPDILHLLIQAQKGALKDDSVEENKGFVGTESKTVGNIKKLDDDDIAAQAFLFFLGGFETTSILM
CFTIFELALNPDIQERLLNEIQNTLSESNGEFSYDVVHKMKYLDMIVTETLRKWPPAPITDRVTTKN
YKIQVGNFSL

>ON646389 CYP9GK5 [386 AA] TRINITY_DN48962_c0_g2_i5|m.41740
MLVLSLFIILIAIIYFYFIRPLNYWKNLDVPFIPGYPIVGNFIWTSLKKQSFTDELNSLYNAFPNEKCV
GMFSFGTAPALLIRDPELLKQITIKDFNYFTDHTKSFFKDGFDTLIDRNLFTLSGNEWRDMRATLSP
AFTGSKMRGLFNMMNDCSEQFVSYYSNKVKNAKDSHLEVELKKSFTRFTNDIIASAVFGVKCDS
LNNEQNEFYLMGCKAAYFATWRILLSNFCPWILKFLNLGGVIPEECCKYFKNLVYETKAVREREN
IIRPDILHLLIQAQKGALKDDSVEENKGFVGTESKTVGNIKKLDDDDIAAQAFLFFLGGFETTSILM
CFTIFELALNPDIQERLLNEIQNTLSESNGEFSYDVVHKMKYLDMIVTGKPRKK

>ON646390 CYP9GK5 [522 AA] TRINITY_DN48962_c0_g2_i2|m.41737
MLVLSLFIILIAIIYFYFIRPLNYWKNLDVPFIPGYPIVGNFIWTSLKKQSFTDELNSLYNAFPNEKCV
GMFSFGTAPALLIRDPELLKQITIKDFNYFTDHTKSFFKDGFDTLIDRNLFTLSGNEWRDMRATLSP
AFTGSKMRGLFNMMNDCSEQFVSYYSNKVKNAKDSHLEVELKKSFTRFTNDIIASAVFGVKCDS
LNNEQNEFYLMGCKAAYFATWRILLSNFCPWILKFLNLGGVIPEECCKYFKNLVYETKAVREREN
IIRPDILHLLIQAQKGALKDDSVEENKGFVGTESKTVGNIKKLDDDDIAAQAFLFFLGGFETTSILM
CFTIFELALNPDIQERLLNEIQNTLSESNGEFSYDVVHKMKYLDMIVTETLRKWPPAPITDRVTTKN
YKIQVGKKLITLEPGFPITVPIIGFHRDPQYYPNPEKFDPERFNDENKKNIKPLTFCPFGAGPRLCIGA
RFAVMNAKIIIFHMIKNFEVILVKNSTVPPVLSSKSGNTILENGFHVGLKLRKL

>ON646391 CYP9GP3 [506 AA] TRINITY_DN48929_c0_g1_i3|m.42062
MFWLIVGILAVILLYKFHNYIVSPFSYWKNKGIDYHLLHDPYTDIFKFVTNTVNSFEEDLANYNGM
PDKKVYGIVQFRNPALMIKDPDLLKQVLIKDFDHFRNHSNVLGEEDSVLTRNLFISKDKKWHNSR
TILSPVFTANKMRIIFSLMASCGDQIVAYFNKEYEKQGNGKPLEIEFKSTTARFTNDVIATTSFGLEI
DSFKNPENEFIKTGRAIFDFSMIRFILLFKFPFIAKIFNVKLMAPIVYEFFPKVIRETLKNRRENNIKRP
DMIQLMMEAKESLEKNPVKDLVLEDDDIISQAFVFFGAGFETVSVAMSFAMYELAIHPEIQKRLQ
EECIENFKKGDGKITYELLQGMKYLDMVVSESFRKWPPATSLDRVCNKKYTLNVDDQEIVIEPEM
FIICPVLGIHRDPKYYPNPDKFDPERFNDENKASIHPMTYMPFGAGPRICIASRFAIMEVKLFLCYIL
ANFNLEPTEKTPIPLKIKMSGFTYTAKDGFWIGLKPREQ

>ON646392 CYP9GP2 [444 AA] TRINITY_DN48929_c0_g1_i4|m.42063
MFWLIVGILAVILLYKFHNYIVSPFSYWKNKGIDYHLLHDPYTDIFKFVTNTVNSFEEDLINYNAKP
DKKVYGIVQFRIPALLIKDPDLLKQVLIKDFDHFTNHSNILREDDPILTKNLFISRDKKWHDTRTILS
PVFTANKMRIIFSLMASCGDQIVAYFNKEYEKQGNGKPLEIEFKSTTARFTNDVIATTSFGLEIDSF
KNPENEFIKTGRAIFDFSMIRFILLFKFPFIAKIFNVKLMAPIVYEFFPKVIRETLKNRRENNIKRPDMI
QLMMEAKESLEKNPVKDLVLEDDDIISQAFVFFGAGFETVSVAMSFAMYELAIHPEIQKRLQEECI

ENFKKGDGKITYELLQGMKYLDMVVSESFRKWPPATALDRVCNKKYTLNVDDQEIVIEPEMLIN
CPVLGIHRDPKYYPNPDKFDPERFNDENKPSIHPMTYMPFGA

>ON646393 CYP9GP2 [431 AA] TRINITY_DN48929_c0_g2_i4|m.42067
MFWLIVGILAVILLYKFHNYIVSPFSYWKNKGIDYHLLHDPYTDIFKFVTNTVNSFEEDLANYNGM
PDKKVYGIVQFRIPALLIKDPDLLKQVLIKDFDHFTNHSNILREDDPILTKNLFISRDKKWHDTRTIL
SPVFTANKMRIIFSLMASCGDQIVAYFNKEYEKQGNGKPLEIEFKSTTARFTNDVIATTSFGLEIDSF
KNPENEFIKTGRAIFDFSILRFILLHKFPTIAKIFNVKLMAPIVYDFFPKVIRDALKNRRENNIKRPDM
IQLMMEAKESLEKNPVKDLVLEDDDIISQAFVFFGAGFETVSVAMSFAMYELAIHPEIQKRLQEEC
IENFKKGDGKITYELLQGMKYLDMVVSESFRKWPPATALDRVCNKKYTLNVDNQEIVIEPEMLIN
CPVLGIHRDPKYYPNPDKFDPERFNDENK

>ON646394 CYP9GP2 [506 AA] TRINITY_DN48929_c0_g1_i5|m.42068
MFWLIVGILAVILLYKFHNYIVSPFSYWKNKGIDYHLLHDPYTDIFKFVTNTVNGFEEDWANYNG
MPDKKVYGIVQFRIPALLIKDPDLLKQVLIKDFDHFTNHSNILREDDPILTKNLFISRDKKWHDTRT
ILSPVFTANKMRIIFSLMASCGDQIVAYFNKEYEKQGNGKPLEIEFKSTTARFTNDVIATTSFGLEID
SFKNPENEFIKTGRAIFDFSILRFILLHKFPTIAKIFNVKLMAPIVYDFFPKVIRDALKNRRENNIKRP
DMIQLMMEAKESLEKNPVKDLVLEDDDIISQAFVFFGAGFETVSVAMSFAMYELAIHPEIQKRLQ
EECIENFKKGDGKITYELLQGMKYLDMVVSESFRKWPPATSLDRVCNKKYTLNVDDQEIVIEPEM
FIICPVLGIHRDPKYYPNPDKFDPERFNDENKASIHPMTYMPFGAGPRICIASRFAIMEVKLFLCYIL
ANFNLEPTEKTPIPLKIKMSGFTYTAKDGFWIGLKPREQ

>ON646395 CYP9GP1 [367 AA] TRINITY_DN48929_c0_g3_i2|m.42065
MFWLIVSVLAVILLYKFHNYILSPFFYWKNKKIDYHLIRGPYADLLKFVTNTVNIFEEDLINYNAKP
DKKVYGTVTFRRPSLLIKDPELLKQILIKDFDHFTNHATLLSEADPLLSDNLFIAKDKKWHNTRTIL
SPVFTANKMRITIDLMALCGEQMISYFKKEYEKQGNGKPLEIEFQSTTGLFSNDVIATTSFGIQCDS
FKAPENEFIKTGRAIFNFSMLRFMLLHLTPSIAKMFDVKIMPPIVYEFFPRVIKEALKNRRENKVFRP
DMIQLLMEAKESLEKNPVKDLALSDDEIISQALVFFAAGFETVAATMTFAIYELTIHPEIQKRAQEE
CVEAFKKGGGKLTYEAINEMKYMDMVVSGKF

>ON646396 CYP9GP1 [524 AA] TRINITY_DN50686_c2_g1_i1|m.51542
MFWLIVSVLAVILLYKFHNYILSPFFYWKNKKIDYHLIRGPYADLLKFVTNTVNIFEEDLINYNAKP
DKKVYGTVTFRRPSLLIKDPELLKQILIKDFDHFTNHATLLSEADPLLSDNLFIAKDKKWHNTRTIL
SPVFTANKMRITIDLMALCGEQMISYFKKEYEKQGNGKPLEIEFQSTTGLFSNDVIATTSFGIQCDS
FKAPENEFIKTGRAIFNFSMLRFMLLHLTPSIAKMFDVKIMPPIVYEFFPRVIKEALKNRRENKVFRP
DMIQLLMEAKESLEKNPVKDLALSDDEIISQALVFFAAGFETVAATMTFAIYELTIHPEIQKRAQEE
CVEAFKKGGGKLTYEAINEMKYMDMVVSETLRKWPPGTNTDRVCNKKYSLKVDNQEIVIEPEM
LITVPVLGIHRDPKYYPNPDKFDPERFNDENKSSIHPQTYMPFGAGPRICIASRFAIMEVKLFLCYIL
ANFNLEVIEKTPIPLKLKMAGLTGYTAKDGFWIGLKPRQ

>ON646397 CYP9GP4 [378 AA] TRINITY_DN48929_c0_g2_i3|m.42066
MVSKRIGLIIMGCRIKKVYGIVQFRNPALMIKDPDLLKQVLIKDFDHFRNHSNVLGEEDSVLTRNL
FISKDKKWHNSRTILSPVFTANKMRIIFSLMASCGDQIVAYFNKEYEKQGNGKPLEIEFKSTTARFT
NDVIATTSFGLEIDSFKNPENEFIKTGRAIFDFSILRFILLHKFPTIAKIFNVKLMAPIVYDFFPKVIRD
ALKNRRENNIKRPDMIQLMMEAKESLEKNPVKDLVLEDDDIISQAFVFFGAGFETVSVAMSFAMY
ELAIHPEIQKRLQEECIENFKKGDGKITYELLQGMKYLDMVVSESFRKWPPATALDRVCNKKYTL
NVDNQEIVIEPEMLINCPVLGIHRDPKYYPNPDKFDPERFNDENK

>ON646398 CYP301B1 [526 AA] TRINITY_DN49028_c0_g1_i2|m.42573
MKCINKNIYKQIDYLNKLFVCDYRLLSSVHHNVSNSNNITSSGTTTIVAGDATQTEWNNAVPYEL
MPGPKPLPLLGNTWRFIPYIGNFEIEHIDQVSLRLYHQYGNIVKISGLLGRPDMVFVYDPDDIERVF
RIEDSIMPHRPSMPSLNYYKHVLRKDFFGEDAGVIAVHGKNWHNFRTKVQQSMLQPRIAKLYIKP
IEDTANAFVERVGLIRDQNNEMPDDFLNYVHKWSLESIAKVALDVRLGCLDEPSNAETQCMIDAV
NTFFINVPVLELKIPFWKLFNTPTWNKYIKALDTITEISLKHINKALDNLKQGNVGSEPSLVQRILQS
EKNPKIACILALDMFLVGIDTTSNAVASILYQLSLHPEKQENLFTELQKVLPDKNTNITSKHMEEM
PYLRACIKETLRMYPVVIGNGRCVTNDCVIRGYQVPKGVQIIFQHYVISNLEKYFYKPNEFLPERW
LKTDAKAECKAHHPFASLPFGHGRRMCLGRRFADLEMQMIVAKVNSRKAFVHLLDPYTGKYLTF

>ON646399 CYP301B1 [542 AA] TRINITY_DN49028_c0_g1_i4|m.42575
MKCINKNIYKQIDYLNKLFVCDYRLLSSVHHNVSNSNNITSSGTTTIVAGDATQTEWNNAVPYEL
MPGPKPLPLLGNTWRFIPYIGNFEIEHIDQVSLRLYHQYGNIVKISGLLGRPDMVFVYDPDDIERVF
RIEDSIMPHRPSMPSLNYYKHVLRKDFFGEDAGVIAVHGKNWHNFRTKVQQSMLQPRIAKLYIKP
IEDTANAFVERVGLIRDQNNEMPDDFLNYVHKWSLESIAKVALDVRLGCLDEPSNAETQCMIDAV
NTFFINVPVLELKIPFWKLFNTPTWNKYIKALDTITEISLKHINKALDNLKQGNVGSEPSLVQRILQS
ENNPKIACILALDMFLVGIDTTSNAVASILYQLSLHPEKQENLFTELQKVLPDKNTNITSKHMEEM
PYLRACIKETLRMYPVVIGNGRCVTNDCVIRGYQVPKGVQIIFQHYVISNLEKYFYKPNEFLPERW
LKTDAKAECKAHHPFASLPFGHGRRMCLGRRFADLEMQMIVAKMIRNYKIEYHYKKLDYFIHPM
YTPNGPLRLRLLDRQS

>ON646400 CYP4420A1 [516 AA] TRINITY_DN49399_c0_g1_i1|m.43968
MPYFIIFNTLLLLLTILTFIIYLYLTWNYNYWKERNIPFVKPSLIFGNIKDQILMRKSFSDVFHEIYTN
FNDCKYVGFYLWRKPALVIRDPELIKMVWSKQFSNFYNNAWYLSEKVDPYFSKSPFFENDEKWK
ELRHQLTPTLSPGRIKSLFPVFLQGIDELHNFIKKNAINDKNEIDLRDLCSKYTTEVSTQAFFGVKA
HAFDNENSDFLNVGKLIFRNSVRSSFESICMMCDKLFAHYFRVKIVNEKAFDFFKKLAVEIMEYRK
TNNNCQYNDYFEHLLGLRETYKEYEDVNNIARQLITVFFDSADTTASGITIAMFQIIRHPEVYKKL
RNEIDTILLKYNGQLTVEAIQDMNYLDMCISEAFRLNVPLYFSLRDCTVPTEFPMPKEAQTDGSLL
VEPGITIIVPMHEIHRDLKYFNDPDVYDPERFSDERKSTIQKGTYFPFGDGPRMCLGKNFAIAAVKH
FIIALVQNYDIKFKPNSNYTLTPNVGIMLNVVLFKQKFMLNFAKRGEFYVE

>ON646401 CYP4418B1 [436 AA] TRINITY_DN49382_c1_g1_i1|m.44269
GVYFGRHPVLFVRDSQLARDVMVKYFNHFQDNTININDKSEPLVAKNPFVLKGEQWRSKRGHLT
PGFTALKLKQMFPHMVAVSERLVAYLHQQIDSSQNSIEAKEVASCFATEVFTLSALNITSDSFENP
KSSFKTIAKTMLEGSNLLAFITWTVAIVDPGLSVLLRTKILTKEGADYFRKAVNDVLVHRETNNIV
VNDIFNHFYTLYQKNADFTKDDLVSNVVTLFIDGVETSATTIRCILYEIAAQPRVYKKLRKEIDTIL
SKNNNKFTYDGIQEMTYLDSCISEGLRIYSAVLFAMRQCTLPFEFPSQKDGEPGVKIDKGISVVLPF
YSIHHDENLYPNPEEYKPERFYDDETKVQAKANLFGFGDGPRICLGMRYGLMVTRLGIATIISKLD
VTVNEKSKERAKNTGPSFLFAPNVEPIVDFHKRGTIYKDN

>ON646402 CYP4XL1 [384 AA] TRINITY_DN49641_c3_g5_i1|m.45797
GLLTSTGDKWRTRRKILTPAFHFNILKGFIPIFEGQSEHSMEDIDKECPKSESNVIPIIVKHSLYSLCE
TTMGLNIESEQKGIQTYKSNIHEFGDIFTDRMAKPWFYSEITYAFTPQRWRLYTIIKSLRTFTSNVIK
ERLTNWDLTQVQNTKNLDTGKYKLPMMDLLIKQMKLDNSLSYEDIREEVDTFMFEGHDTTSMG
LSLFLMLLANHPEIQNKIYEEMKTIFGDSKRQATYDDLMEMTYLERCIKESLRLYPAVPVVGRTLT
EDLVLKDYVLPAGIYVVYVLIYDLHRNPNYWPNPNKFDPDRFLPENSVKRNPFTYVPFSAGPRNCIG
QKFAILEMKSFVSKIIQEYILEPVDTPQTVSLKTDVVIRPSKPVRVKFRKRQ

>ON646403 CYP4XL1 [507 AA] TRINITY_DN51971_c0_g4_i2|m.58098
MYLVIGLILFVLALILWANREHWQLRIYKHVKDIPGPPSIPFIGNILPYVGTLDVIWKHARNLAIEY
DGLYKIWAGNRCAIFISDPNYNEIILSSTKNIEKSEPYNLLESWLGQGLLISGGEKWKSRRKILTPAF
HFHILKQFINVFEEQSDRTVKMIQEECDKPYTNVIPLAAEHALCALCETTMGIKMDNYENLQDFN
RYKEAIYQFGHLFMDRVKNPLLYSEKIYSYTPMARVTKKLYGILKKFTLRVIDERLASWDDEQKK
IGMTSASGKQLFPMLDILITEMKNGGDINYDGIREEVDTFMFEGHDTTSMGLSFLLMLLAQHPEV
QEKILEEQNQIFNGDNRIATYEDLQNMKYLEMVIKESLRLYPAVPVLGRKVTEDVQLGPYTIPAD
VQVYIVVFDTHHNPNFWPDPYKFDPDRFLPDNCAKRHPFAYIPFSAGSRNCIGQRFVMLEMKSFV
TKILREYILEPVDDLSKVVMKIDLVIRPLNDNLRVKFRRRNDNNNTK

>ON646404 CYP4XL1 [392 AA] TRINITY_DN51971_c0_g4_i8|m.58109
GLLISGGEKWKSRRKILTPAFHFHILKQFINVFEEQSDRTVKMIREECDKPYTNVIPLAAEHALCAL
CETTMGIKMDNYENLQDFNRYKEAIYQFGHLFMDRVKNPLLYSEKIYSYTPMARVTKKLYGILK
KFTLRVIDERLASWDDEQKKIMTSASGKQLFPMLDILITEMKNGGDINYDGIREEVDTFMFEGHDT
TSMGLSFLLMLLAQHPEVQEKILEEQNQIFNGDNRIATYEDLQNMKYLEMVIKESLRLYPAVPVL
GRKVTEDIQLGPYTIPADVQVYIVVFDTHHNPNFWPDPYKFDPDRFLPDNCAKRHPFAYIPFSAGS
RNCIGQRFVMLEMKSFVTKILREYVLEPVDDLSKVVMKIDLVIRPLNDNLRVKFRRRNDNNNTK

>ON646405 CYP4XL2 [470 AA] TRINITY_DN49666_c2_g7_i2|m.45817
MYFIIQFLAQALLSVILVFIATILIIIWKYPIHWKYRIYKLGQDIPGPPSIPFIGNFIPFLGPLDHIWRIFR
KLSLHYNGIFKVWTLNRFSVFISEPDYVEVLLSSAKNLRKSEAYDFLELWLGSGLLISDGDKWKSR
RKLLTPAFHFNILKQFVTVFEEQSERTVQIIRKECDKPFTNVIPISTEHALGALCETTMGIKMNFDD
NNSEFIKYKNAIYHFGEIFMERITKPIFYSQFLFELSSMGKITNKIITTLRNFTLNVIDERLNNWETRN
QQMIKSASGKKLLPMMDILITEMKNGANIDYNGIVEEVDTFMFEGHDTTSMGISFMLMLLASEPE
VQDKILEEQNSIFNGSTRMATYEELQNMEYLERVIKESLRLCPPVPVIGRRLEEDLQMGQYIIPKES
YLYVSIYDLHHNPKYWDDPEKFDPDRFLPDNAAQRHPFAYVPFSAGSRNCIGQRFVMLEMKSFV

>ON646406 CYP9GM1 [446 AA] TRINITY_DN49864_c2_g1_i1|m.46606
MILELIGVLIVIYLLHLYFMKGYDYWEKRGIPSYRRFHPYIEFLNMLFQRTTMYDDIIGVYNGLPD
KKVHGINILKIPQVFVKDPDILRNICVKDFDHFNNHVDIFLQADPLFGKSLFVLKDKKWRDMRNIL
TPVFTGSKLRGLCELISECGTQTMDHYSEMCRQKQNNVLKIDFLDACTRFTNDVIASVAFGIKVNS
LTHPNNEFFHAGHEISKFATWRMFLIFKFPSLCKIFKIRFAPNYVYDFFTNITESTLRARKEQNIMRY
DIIYLLTEAQKEQIKNDPSSKNILTTEDITSQVVIFFLAGYANVAIIMAFAAYELALQPDLQKRLQKE
IDTVLKENNGKLDYDVIKSMKFLDMIVTETLRKWPPSAALDRQCNKSFEIDTNNNETITVKPGMVI
NIPVMGLHYDPKYYPNPEKFDPERFNDENKAKLNPYTYLPFGYGPR

>ON646407 CYP9GM1 [459 AA] TRINITY_DN49864_c2_g1_i3|m.46609
MKIPIYLYFLIHKMILELIGVLIVIYLLHLYFMKGYDYWEKRGIPSYRRFHPYIEFLNMLFQRTTMY
DDIIGVYNGLPDKKVHGINILKIPQVFVKDPDILRNICVKDFDHFNNHVDIFLQADPLFGKSLFVLK
DKKWRDMRNILTPVFTGSKLRGLCELISECGTQTMDHYSEMCRQKQNNVLKIDFLDACTRFTND
VIASVAFGIKVNSLTHPNNEFFHAGHEISKFATWRMFLIFKFPSLCKIFKIRFAPNYVYDFFTNITEST
LRARKEQNIMRYDIIYLLTEAQKEQIKNDPSSKNILTTEDITSQVVIFFLAGYANVAIIMAFAAYELA
LQPDLQKRLQKEIDTVLKENNGKLDYDVIKSMKFLDMIVTETLRKWPPSAALDRQCNKSFEIDTN
NNETITVKPGMVINIPVMGLHYDPKYYPNPEKFDPERFNDENKAKLNPYTYLPFGYGPR

>ON646408 CYP9GM1 [372 AA] CYP9GM1_TRINITY_DN49864_c2_g1_i5|m.46611
MILELIGVLIVIYLLHLYFMKGYDYWEKRGIPSYRRFHPYIEFLNMLFQRTTMYDDIIGVYNGLPD
KKVHGINILKIPQVFVKDPDILRNICVKDFDHFNNHVDIFLQADPLFGKSLFVLKDKKWRDMRNIL
TPVFTGSKLRGLCELISECGTQTMDHYSEMCRQKQNNVLKIDFLDACTRFTNDVIASVAFGIKVNS
LTHPNNEFFHAGHEISKFATWRMFLIFKFPSLCKIFKIRFAPNYVYDFFTNITESTLRARKEQNIMRY
DIIYLLTEAQKEQIKNDPSSKNILTTEDITSQVVIFFLAGYANVAIIMAFAAYELALQPDLQKRLQKE
IDTVLKENNGKLDYDVIKSMKFLDMIVTGNKIITKPFP

>ON646409 CYP9GM1 [391 AA] TRINITY_DN51063_c0_g2_i1|m.53754
FGKSLFALKDKKWRDMRTILSPVFTGSKLRGLCELISECGTQTMDHYSEMCRQKQNNVLKIDFLD
ACTRFTNDVIASVAFGIKVNSLTHPNNEFFHAGHEISKFATWRMFLIFKFPSLCKIFKIRFAPNYVY
DFFTNITESTLRTRKEQNIMRYDIIYLLTEAQKEQIKNDPSSKNILTTEDITSQVVIFFLAGYANVAII
MAFAAYELALQPDLQKRLQKEIDTVLKENNGKLDYDVIKSMKFLDMIVTETLRKWPPSAALDRQ
CNKSFEINTNNNETITVKPGMVINIPIMGLHYDPKYYPNPEKFDPERFNDENKAKLNPYTYLPFGY
GPRVCIASRFVILETKTFLIHLLSKFNVVVTEKTPIPIRLVRGSILKPDTDFGLGLQLRQ

>ON646410 CYP4417A1 [447 AA] TRINITY_DN50148_c0_g1_i1|m.48533
YSNIFLDSKQFLFSYPNAVVLESYNNGYSDIFELLWRVVASGESKLLSSHKTTQKMYAYDLVKYS
FGEGLFLSSGAVYRNSHRLIQPTFHHDVLETYIQLFKKNGDFLVESLRENVGKEAFDIFRKVNLCII
KNVVDTLMDTKMTPEITDELYFSLNRLYNIIHERLFKPWLHPEFIFKLTSIGKEERKSAKYLQNFIR
NVIKSKKMELSLASDDSEYASNSKRRSTLDFLLRKLNENPNVMSEELLVDNISTIFLAAQDATSIQY
SFAVLMFGMYPNFQDKIYEEIQKVLGQESTFTISDLNQLVYLDMFLNECLRLLPIPFIGRKVTEDLQ
FENFVAPAGCSIIVNTYEIHRNPKYWEKPNEFYPEHFTPENVAKRHPMAFIPFSAGPRRCIGRQYAV
YVLKTLIISTLRSYKIEADGTLQDLNITGDLACRVKDGYKIRIKYRN

>ON646411 CYP4417A1 [495 AA] TRINITY_DN50148_c0_g3_i1|m.48534
MGVEILYPLVYSFLITFLIAYYWKRRHLYIASFKIDGPISFPLIGNSYLFFGSSQEDVFSKSMEMIQSY
SSTCRFWRGSQLLICIKNIKDIEKLLSSHKTTQKMYAYDLVKYSFGEGLFLSSGAVYRNSHRLIQPT

FHHDVLETYIQLFKKNGDFLVESLRENVGKEAFDIFRKVNLCIIKNVVDTLMDTKMTPEITDELYF
SLNRLYNIIHERLFKPWLHPEFIFKLTSIGKEERKSAKYLQNFIRNVIKSKKMELSLASDDSEYASNS
KRRSTLDFLLRKLNENPNVMSEELLVDNISTIFLAAQDATSIQYSFAVLMFGMYPNFQDKIYEEIQ
KVLGQESTFTISDLNQLVYLDMFLNECLRLLPIPFIGRKVTEDLQFENFVAPAGCSIIVNTYEIHRNP
KYWEKPNEFYPEHFTPENVAKRHPMAFIPFSAGPRRCIGRQYAVYVLKTLIISTLRSYKIEADGTLQ
DLNITGDLACRVKDGYKIRIKYRN

>ON646412 CYP4417A1 [384 AA] TRINITY_DN50148_c0_g1_i2|m.48535
MGVEILYPLVYSFLITFLIAYYWKRRHLYIASFKIDGPISFPLIGNSYLFFGSSQEDVFSKSMEMIQSY
SSTCRFWRGSQLLICIKNIKDIEKLLSSHKTTQKMYAYDLVKYSFGEGLFLSSGAVYRNSHRLIQPT
FHHDVLETYIQLFKKNGDFLVESLRENVGKEAFDIFRKVNLCIIKNVVDTLMDTKMTPEITDELYF
SLNRLYNIIHERLFKPWLHPEFIFKLTSIGKEERKSAKYLQNFIRNVIKSKKMELSLASDDSEYASNS
KRRSTLDFLLRKLNENPNVMSEELLVDNISTIFLAAQDATSIQYSFAVLMFGMYPNFQDKIYEEIQ
KVLGQESTFTISDLNQLVYLDMFLNECLRLLPIPFIGRKVTEDLQFGN

>ON646413 CYP4417A1 [392 AA] TRINITY_DN50148_c0_g1_i5|m.48536
MYAYDLVKYSFGEGLFLSSGAVYRNSHRLIQPTFHHDVLETYIQLFKKNGDFLVESLRENVGKEA
FDIFRKVNLCIIKNVVDTLMDTKMTPEITDELYFSLNRLYNIIHERLFKPWLHPEFIFKLTSIGKEER
KSAKYLQNFIRNVIKSKKMELSLASDDSEYASNSKRRSTLDFLLRKLNENPNVMSEELLVDNISTIF
LAAQDATSIQYSFAVLMFGMYPNFQDKIYEEIQKVLGQESTFTISDLNQLVYLDMFLNECLRLLPIP
FIGRKVTEDLQFENFVAPAGCSIIVNTYEIHRNPKYWEKPNEFYPEHFTPENVAKRHPMAFIPFSAG
PRRCIGRQYAVYVLKTLIISTLRSYKIEADGTLQDLNITGDLACRVKDGYKIRIKYRN

>ON646414 CYP9GK2 [409 AA]
TRINITY_DN50479_c1_g1_i4|m.50192MFYILLGLAVVIFLYREIYRSLYYWEDKGIKQVRSIPIVGN
FIWSILQRTPFAESSQLYYNALPNEKCIGIMAFNKPTLVIRDKNLIKQIGIKDFDNFTDHQKLFF
KEGFDRLFDRNLFSLDGQPWRDMRATLSPAFTGAKMRGMFQIINECGEQMAAFYLNEIKKL
GGDSKHIELELKDSFTRFTNDVIATSAFGVKCDSFKDKNNEFYSNGRNATQFQTWRIVLVNFI
PAVMKMLNIYTIPISVCDYFRNIVKETVTVRERENIVRPDMLHLLMQAQKGTLKTENQIEDN
KGFAAVNESTNAHGKAKLLDDEDVAAQAFIFFLAGFETVSTAMCFTSYELALNPDIQQRCYE
EIQEIVESNDGKLSYDVIQKIKFLDMVISESLRKWPPAIFTDRVCTKPYTFNVNG

>ON646415 CYP9GK2 [372 AA] TRINITY_DN51063_c1_g3_i1|m.53766
EQMAAFYLNEIKKLGGDSKHIELELKDSFTRFTNDVIATSAFGVKCDSFKDKNNEFYSNGRN
ATQFQTWRIVLVNFIPAVMKMLNIYTIPISVCDYFRNIVKETVTVRERENIVRPDMLHLLMQA
QKGTLKTENQIEDNKGFAAVNESTNAHGKAKLLDDEDVAAQAFIFFLAGFETVSTAMCFTSY
ELALNPDIQQRCYEEIQEIVESNDGKLSYDVIQKIKFLDMVISESLRKWPPAIFTDRVCTKPYTF
NVNGKDVLLEKGTNIQFPIYAIHRDPQNYPNPEKFDPERFSDENRHSIDPMTYLPFGVGPRICI
GSRFALMEVKIVIVHLVKNFEIILTKKSTVPPRMTRKSTNMTIEGGFNVGLKARTH

>ON646416 CYP9GN3 [441 AA] TRINITY_DN50416_c2_g2_i2|m.50480
VYAFGLPQFMIKDPDIIRNICVKDFDHFVNHIDAFFKHDRLFGKSLFALKDKKWRDMRTIMTP
VFTGSKLRGLVQLLSECGEQINLHYAQKYKDQDDKVLKIDIMDISTRFTNDVIASAVFGIKVN
SLTDPENEFYLAGNEIIKFPAWRFIMIMMLPSLSKLTKMGFASKHIYDFFIKLVEDTIRIRTERNI
IRYDIIHLLLEARKEQIKAIETKRASNENVDVLSKNLLTDEDITAQAFIFFLAGYSNVAILMSFA
AYELAVHPDVQVKLQKEIESLIKENGKLDYDVIKKMKYLDMVVSETLRKWPPLPAIDRECN
KAYKIETNNDIIEVKPGMIISIPVLGLHYDPKYFPNPNKFDPERFNDENKAKMNPYTYMPFGIG
PRICIANRFVILETKLFLINLLLKFDILPTEKTPIPLVLAGGNVLKPKDDFSLGLKLRN

>ON646417 CYP9GN3 [366 AA] TRINITY_DN50852_c3_g1_i3|m.52316

MIFVILATIIALGLAHYYFTKQFKYWEEHGIIHHIKCNRYMETYRFLFKKFVMYDDLMEDYN
VFPDKQVYGVYAFGLPQLMIKDPDIIRNICVKDFDHFVNHIDAFFKHDRLFGKSLFALKDKK
WRDMRTIMTPVFTGSKLRGLVQLLSECGEQINLHYAQKYKDQDDKVLKIDIMDISTRFTNDV
IASAVFGIKVNSLTDPENEFYLAGNEIIKFPAWRFIMIMMLPSLSKLTKMGFASKHIYDFFIKLV
EDTIRIRTERNIIRYDIIHLLLEARKEQIKAIETKRASNENVDVLSKNLLTDEDITAQAFIFFLAG
YSNVAILMSFAAYELAVHPDVQVKLQKEIESLIKENGKLDYDVIKKMKY

>ON646418 CYP9GN4 [398 AA] TRINITY_DN50860_c2_g1_i3|m.52536
HQDVFFRNDEMLGKNLFALKDKKWRDMRTILSPVFTGSKLRGLCELMSECGSQTVTYYVQK
MKQQNNNNPLKVDIFDACTRFTNDVIASTAFGIKVNSLADKNNEFFGVGQKVSSFSSWRLWL
FNLPLFVKQAFNIKIFPEDVYKFFRQIVNETIRVRAEKNIIRHDMIHLLMEARKEQIKRTEASNG
KVDNISASNNVLTNDDITAQGVIFFIAGFSNVALAISMASYEMAVNPEIQKKAYAEITEVLKA
NNGKIDYDVIKQMKYLEMIISETLRKWSPVVVGDRVCNKAFKIETPNDGVINVEPGMTINIPIS
GLHNDPKYFPNPKKFDPERFNDTNKSKMNPYTYIPFGLGPRICIGKYVIFVETHAWRKMACSL
WTHKNILSEVLNNSVHTINW

>ON646419 CYP9GN4 [410 AA] TRINITY_DN50860_c2_g1_i4|m.52538
HQDVFFRNDEMLGKNLFALKDKKWRDMRTILSPVFTGSKLRGLCELMSECGSQTVTYYVQK
MKQQNNNNPLKVDIFDACTRFTNDVIASTAFGIKVNSLADKNNEFFGVGQKVSSFSSWRLWL
FNLPLFVKQAFNIKIFPEDVYKFFRQIVNETIRVRAEKNIIRHDMIHLLMEARKEQIKRTEASNG
KVDNISASNNVLTNDDITAQGVIFFIAGFSNVALAISMASYEMAVNPEIQKKAYAEITEVLKA
NNGKIDYDVIKQMKYLEMIISETLRKWSPVVVGDRVCNKAFKIETPNDGVINVEPGMTMNIPI
SGLHNDPKYFPNPKKFDPERFNETNKLKINPYTYIPFGLGPRICIANRFVLIETKILLFHILKTFEI
VTTDETPIPMVLEKGAVIKPKGDTYGLAF

>ON646420 CYP305W3 [494 AA] TRINITY_DN50925_c0_g2_i2|m.52722
AVLFLILLSVFVILLIIRSIKKPYNYPPGPSWLPIVGCQPTFKKKLMKHEGAMYLGLQELADEY
NTNILGLKLGKDQIICVFGYERVKEVLTNDDFAGRPDGFFLRLRTFGMRKGITFTDGPVWHE
QRSFVMRHLRDVGFGKKSMELKIINECQDLIQFINENGPDINMEEFFAPSVLSVLWDLTAGRS
LDRNDDKLKELLALLKRRLKAFDLSGGLLGQMPWLRFIIPEKSGFNLVTKLNEQMRNFFMEA
ILEHHATFTGDDSDLIYAFIQEMKIQQKEEKKDSTFTNDQLLAVCLDLFIAGSQTTNSLSGFLF
YNLLCYPDIKVKILNEIDANIPKSRMPELADRVKLPYLEAFIMETRRMQPVAGVAGPRRVLHD
YQMDKYNLEKDVTVLISIWSVHMDKEYWKDPEVFRPERFIGKDGLLYPNERILNFGLGKRRC
LGEALGKDCIFLFFTGILQKFDVLPVSNIPLPLKRAYAGITMTPDPFSIRFVNR

>ON646421 CYP305W2 [364 AA] TRINITY_DN50925_c0_g1_i2|m.52723
MAVLFLILLSVSVILLIIRSIKKPYNYPPGPSWLPIVGCQPIFKKRLMKHAGAMYLGLQELADE
YNTNILGLKLGKDQIICVFGYERVKEVLTNDDFAGRPDGFFLRLRTFGMRKGITFTDGPVWH
EQRSFVMRHLRDVGFGKKSMELKIINECQDLIQFINENGPDINMEEFFAPSVLSVLWDLTAGR
SLDRNDDKLKELLALLKRRLKAFDLSGGLLGQMPWLRFIIPEKSGFNLVTKLNEQMRNFFME
AILEHHATFTGDDSDLIYAFIQEMKIQQKEEKKDSTFTNDQLLAVCLDLFIAGSQTTNSLSGFL
FYNLLCFPDIKVKILKEIDANIPKSRMPELADRVKYVYFTKNFALIMYD

>ON646422 CYP305W2 [495 AA] TRINITY_DN50925_c0_g2_i5|m.52729
MAVLFLILLSVSVILLIIRSIKKPYNYPPGPSWLPIVGCQPIFKKRLMKHAGAMYLGLQELADE
YNTNILGLKLGKDQIICVFGYERVKEVLTNDDFAGRPDGFFLRLRTFGMRKGITFTDGPVWH
EQRSFVMRHLRDVGFGKKSMELKIINECQDLIQFINENGPDINMEEFFAPSVLSVLWDLTAGR
SLDRNDDKLKELLALLKRRLKAFDLSGGLLGQMPWLRFIIPEKSGFNLVTKLNEQMRNFFME
AILEHHATFTGDDSDLIYAFIQEMKIQQKEEKKDSTFTNDQLLAVCLDLFIAGSQTTNSLSGFL

FYNLLCFPDIKVKILKEIDANIPKSRMPELADRVKLPYLEAFIMETRRMQPIAGVAGPRRVLHN
YQMDKYLLAKDVTVLISIWSVHMDKEYWKDPEVFRPERFIGKDGLFYPNERILNFGLGKRRC
PGEALAKDCIFLFFASILQKFDVLPVSNIPLPLKRAYAGITMTPDPFSIRFVNR

>ON646423 CYP305W2 [448 AA] TRINITY_DN50925_c0_g2_i7|m.52731
MAVLFLILLSVSVILLIIRSIKKPYNYPPGPSWLPIVGCQPIFKKRLMKHAGAMYLGLQELADE
YNTNILGLKLGKDQIICVFGYERVKEVLTNDDFAGRPDGFFLRLRTFGMRKGITFTDGPVWH
EQRSFVMRHLRDVGFGKKSMELKIINECQDLIQFINENGPDINMEEFFAPSVLSVLWDLTAGR
SLDRNDDKLKELLALLKRRLKAFDLSGGLLGQMPWLRFIIPEKSGFNLVTKLNEQMRNFFME
AILEHHATFTGDDSDLIYAFIQEMKIQQKEEKKDSTFTNDQLLAVCLDLFIAGSQTTNSLSGFL
FYNLLCFPDIKVKILKEIDANIPKSRMPELADRVKLPYLEAFIMETRRMQPIAGVAGPRRVLHN
YQMDKYLLAKDVTVLISIWSVHMDKEYWKDPEVFRPERFIGKDGLFYPNERILNFGLGKIQF
DKKDFSF

>ON646424 CYP4G280 [567 AA] TRINITY_DN51045_c2_g1_i1|m.53567
MSTTFTSLDENTSSWSVAGATLYGLITSATLLVIYYYWEQNKRFARLGNAIPGPPGVPFLGNA
LEIFLSTPEELLVKALEYSKHYGKVVKGWLGPYLVVFLIDPRDVEVILNSHVHIDKSDEYKFF
KPWLGEGLLISTGEKWRTHRKMIAPTFHMNILKSFMDVFNRNSLAVVKKMRKEIDREFDVH
DYMSGITVDILLETAMGVKRTHSDNAGFDYAVAVMKMCDILHQRHYKFWMRFEPLFKLTN
FAKKQLEYLGIIHGLTNKVIKTKKEEFLQNRVLGNEPNYFEQLDSDAAKIDQKEAKLSSQNN
QSSDPNKGTPLRDDLDEIDEQDVGEKKRLAFLDLMIETAHSTQNLTDEEIKEEVDTIMFEGHD
TTAAGSSFVLCLLGIYKDVQDKVYQELYDIFGNSDRPVTYLDTLEMKYLERVIMETLRLYPP
VPIIARKMNQEVKLASGDYTLPVGCTVVIGTYKIHHDPDYYKNPDTFDPDNFLPERTQNRHY
YSYIPFSAGPRSCVGRKYAMLKLKVLLSTILRNYTIVSDLTEKDFRLTGDIILKRADGFRIRIQP
RERVPV

>ON646425 CYP4416A7 [489 AA] TRINITY_DN51185_c2_g2_i9|m.54068
KIYIWNYIRMCYYASKLPGPPAYPIIGNGALLFGSHEEASHNLLDICLQYQKSLIRFWLGPIPV
AVILDPEDIQEVLTNSNALEKSFLYHLIWDTTMGKSIFTSEVLEWKHHRGIIVKGFSPIILKSYF
KIFVEKINILTENLDLELNKETSFDIFKCLSRTTLDSVCGSTMGVNINSHKHSESYFDAMHRLM
NLMIVRLFSVIKLPFLLYRFTEDYKIASKCSKIIREFTENIISEKRSKYETIDNNNLIDVKANKKN
EYKKPLLEYLMDFTKNDEEFTDTQLRDEVNTIIFAAYETSANALSYVLLNLAAHKDIQTKVY
NEILEVLGENNESSINFDDLPKFKYMEMVIKESMRRYSTVPFIGRKACQDIKIGNYTIPKGITIG
IMIMAAHLNENHWENPLKFYPERFTPENIKNRHPYSFIPFSAGSRNCIGIRYAWIVMKITLATL
LSRYEFHTDLNLDELRFQFNLTLSLVNGHQVRITPRQKL

>ON646426 CYP4G282 [559 AA] TRINITY_DN51463_c0_g1_i2|m.55589
MTLIDAAGAEFASGPSPVFSATSVLYFLLAPAIALYLLYWRLSRNHMIKLAEKIPGPTGYPIIG
NALDLIGKSPDIFKHVYARSFEFGSVVRLWIGPKLVVFLTDPRDVEVILSSHVYIDKSSEYRFF
QPWLGNGLLISTGPKWRAHRKLIAPTFHLNVLKSFIDLFNANSRAVCAKMLKEAGKTFDCHD
YMSECTVEILLETAMGVSKNTQESGYDYAMAVMKMCDILHLRHTKFWLRPDWLFNLTRHG
KTQVALLDTIHSLTRKVIKRKKEDFKNKKITPVEIKKEVEEVTHSDTKGAVDGLSYGQSAGLR
DDLDVDDNDVGEKKRMAFLDLMIEASQNGVNITDEEIKEQVDTIMFEGHDTTAAGSSFFLSM
MGIHQHIQDKVIQELDGIFGDSDRPATFADTLEMKYLERCLMETLRLYPPVPIIARQLNEDLK
LASAPYVLPAGCTVVVGTVKLHRREEIYPNPDEFNPDNFLPEKTANRHYYAFVPFSAGPRSC
VGRKYAMLKLKILLSTILRNFRVKSDLKEKDFQLQADIILKRAEGFQVRLEPRKRTAKA

>ON646427 CYP4XE2 [484 AA] TRINITY_DN51460_c5_g2_i1|m.55692

MFLLLLKIFFTLFIGLLIILYLNQLRVCRRYPVPSPPLIPILGHIIELGSTAEAIHTNILRLIDTYGK
VIQLWIGPFDHYYFISDIDITNKILSSTKLIEKSHAYDLLAPWLGYGLLLSGGEKWHKRRKML
TPAFHFTILEQSINTFTIASDIFVKNLKKHVDGGNFDVYPYITLLGLDIICQTAMGIDINAQMQS
DSEYVTAVRDVCQIVFERIFSAIQQSDFIFYNFTKNGKVFKKSLKILHNLTNKVIEERRQLLIDS
MKNNTTKPNKIINKEVIDDLEVQEKKEKMVFMDVLLKSTIDGKPLTNSDIREEVDTFMFEGH
DTVSSGLSFTLYLLANHPEVQNQVFEEIREICGDDLKKHATLSDLNKMTYLEAVIKESMRIYP
PVPIVGRKLSEDLPLENGVLRKNTRPSIMIYSIHHNPEYFPDPEAFKPERFLTQAQNKHYFSYIP
FSAGSRNCIGQKFAMYEMKSTITNVIRHYKLFS

>ON646428 CYP9GK3 [487 AA] TRINITY_DN51572_c0_g2_i2|m.56121
MFTIFDQQEDLLTKNKFFNKICVFRLYGMFMFNTPTILVRDLDMLKNILIKQFEYFVDHSNSL
FQEDINPMLDKNLFSLKGNKWRDMRATLSPTFTGSKMRGMFQLINEVGEQFVDYFKNEIQT
KNNGKPMNVELKDILTRFTNDVIASAAFGVKCDSLRDRNNEFYRMGAKVSNLSTWRMFINN
FCPKLAKFFRIGLIPNEVCDYLRNIVIETIKMRERENIVRPDMLQLLLDAKNGTIKQDKQKIET
DNSFAAVEESNYTQIKKRIYLDEDDVAAQAFVFFMGGFDTVSIAMCFSVYELALHPELQERL
YLENEQVLSENNGKLTYDVLNKMKYLDMVISETLRKYPPPIFLDRICDKPYTVQIDGKEITLE
KGTILTIPLYAMHTDPNYFPEPEKFDPHRFSDENADKIVPLTYIPFGSGPRNCIASRFALMEAKT
AVYFMVKHFEILPNEKSIIPPRITTKRINVTMEDGFHVTLKPRNQGFI

>ON646429 CYP9GK3 [416 AA] TRINITY_DN51572_c0_g3_i1|m.56124
LDKNLFSLKGNKWRDMRATLSPTFTGSKMRGMFQLINEVGEQFVDYFKNEIQTKNNGKPMN
VELKDILTRFTNDVIASAAFGVKCDSLRDRNNEFYRMGAKVSNLSTWRMFINNFCPKLAKFF
RIGLIPNEVCDYLRNIVIETIKMRERENIVRPDMLQLLLDAKNGTIKQDKQKIETDNSFAAVEE
SNYTQIKKRIYLDEDDVAAQAFVFFMGGFDTVSIAMCFSVYELALHPELQERLYLENEQVLS
ENNGKLTYDVLNKMKYLDMVISETLRKYPPPIFLDRICDKPYTVQIDGKEITLEKGTILTIPLY
AMHTDPNYFPEPEKFDPHRFSDENADKIVPLTYIPFGSGPRNCIASRFALMEAKTAVYFMVKH
FEILPNEKSIIPPRITTKRINVTMEDGFHVTLKPRNQGFI

>ON646430 CYP9GK3 [530 AA] TRINITY_DN51572_c0_g2_i5|m.56126
MLIVVLVIFVIIVAAYWLLITPLSHWEKRGIPYVKGLPIVGNFFWHTMKQTTFGDELVKIYNT
YPEKKLYGMFMFNTPTILVRDLDMLKNILIKQFEYFVDHSNSLFQEDINPMLDKNLFSLKGNK
WRDMRATLSPTFTGSKMRGMFQLINEVGEQFVDYFKNEIQTKNNGKPMNVELKDILTRFTN
DVIASAAFGVKCDSLRDRNNEFYRMGAKVSNLSTWRMFINNFCPKLAKFFRIGLIPNEVCDY
LRNIVIETIKMRERENIVRPDMLQLLLDAKNGTIKQDKQKIETDNSFAAVEESNYTQIKKRIYL
DEDDVAAQAFVFFMGGFDTVSIAMCFSVYELALHPELQERLYLENEQVLSENNGKLTYDVL
NKMKYLDMVISETLRKYPPPIFLDRICDKPYTVQIDGKEITLEKGTILTIPLYAMHTDPNYFPEP
EKFDPHRFSDENADKIVPLTYIPFGSGPRNCIASRFALMEAKTAVYFMVKHFEILPNEKSIIPPR
ITTKRINVTMEDGFHVTLKPRNQGFI

>ON646431 CYP9GL1 [374 AA] TRINITY_DN51572_c0_g1_i4|m.56125
MITIIVGIVTILVLFHYYFTSTYEYWKKRGIPYYQESNAYLESIRIFRKNAMNELIDQYNAFPDK
IAYGIYFFRIPQLMVKDPELIRTICIKDFEHFTNHQGVFFQNDEILGKNLFALKDKKWRDMRTI
LSPVFTGSKLRGLSELMSECGDQTAAYYVQKIKQQNDDKPLKVNIFDACARFTNDVIASTAF
GIKVNSLEDKDNDFFKIGQRVSSFSSWKLWLLGLPAVIREYFKIKIFDNDIYDFFRNIGNETITA
RLEKNIIRPDMIHLLLEARKEQIKRSEEGNGKIENLPQTNSILTNDEIIAQGVIFFVAGFTNVALT
MSFAAYELAVNPKVQEELYAEIKETEKRNAKIDYDTIKQMKYFDMVISGLSK

>ON646432 CYP9GL1 [391 AA] TRINITY_DN51572_c0_g1_i6|m.56131

MITIIVGIVTILVLFHYYFTSTYEYWKKRGIPYYQESNAYLESIRIFRKNAMNELIDQYNAFPDK
IAYGIYFFRIPQLMVKDPELIRTICIKDFEHFTNHQGVFFQNDEILGKNLFALKDKKWRDMRTI
LSPVFTGSKLRGLSELMSECGDQTAAYYVQKIKQQNDDKPLKVNIFDACARFTNDVIASTAF
GIKVNSLEDKDNDFFKIGQRVSSFSSWKLWLLGLPAVIREYFKIKIFDNDIYDFFRNIGNETITA
RLEKNIIRPDMIHLLLEARKEQIKRSEEGNGKIENLPQTNSILTNDEIIAQGVIFFVAGFTNVALT
MSFAAYELAVNPKVQEELYAEIKETEKRNAKIDYDTIKQMKYFDMVISETLRKWTPASATDR
ICNKEFT

>ON646433 CYP4XD1 [414 AA] TRINITY_DN51514_c1_g2_i3|m.56406
MASNVTIFFVIILILFIIKYLWSRRLHWNLRIIKHVKHFPGPPCIPVLGCTIPLLGPLDHIWNEFR
KMAKTYPEMYRLWGGNRCGIFLLEPDDSEILLLSTRNLAKSEGYQYMHDWLGNGLLLSSGS
KWKARRKILTSAFHFNILNEFVNVFEDNSEKSANYIKNNCNKDLDANAIAVDHTMHSLCETT
MGISVNMEDPDIKQYKDALHNFGSVFFNRLMRPWLTADFIYNLSEIKRNQDRLIKAMHKFTL
SVIEGRLLNWSLEGKQPLYSASGKQILPMMDLLIGEMKRGGDIDFEGIREEVDTFMFEGHDTT
SAGLTFLLMLLANHPEIQNRIYDEQLKIFNGSKRKATYDDLQNMEYLERCIKESLRLYPPVPII
GRTLDEDIVLKNKLVMPSGIFANIMIYDLHRNPKFW

>ON646434 CYP4XD2 [463 AA] TRINITY_DN51514_c1_g2_i4|m.56409
LVNKFYNYYDILIIVMASITVLLLVIVILFIIKLLWANRLHWDYRIYQCVKHIPGPYNIPLLGCS
LPFIGTLEHLWDELRRTAKRFPDIYKMWGGNRCGIFLLNADDIEMVLLSTKSLAKNDAYESIR
DWLGDGLLISGGSKWKSRRKILTPAFHFNILNQFVNVFESNSEKSTNFIKNNCNKDLNANDLA
VEHSLYSLCETTMGISIDMENPDIKQYKYALHNFGTTFYNRITRPWLYPDIFFNLSEIKRNQDR
LIKAMHKFTLNVIEERLQNWSLEGKQPLYSASGKQILPMMDLLIGEMKRGGDIDYEGIREEV
DTFMFEGHDTTSTGLTFLLMLLANHPEIQNRIYEEQLTIFNDSKRKATYDDLQNMEYLERCV
KESLRLYPPVPIIGRTLDEDIVLKSGYVMPSGIFVNMMICDLHRNPKYWPDPHKFDPDRFLPD
NCVNRHPFAYIPFSAGSRNCI

>ON646435 CYP9GN2 [398 AA] TRINITY_DN51723_c1_g4_i1|m.57100
LFVLKDKKWRDMRNILTPVFTGSKLRGLVQLISNCGEKTVNHLVEELKEKNTKLLKIDIMDT
TTRFTNDVIATSAFGIEVNSIKDPKNDFFMAGREIIRFPMWRFFMMFFLPAVSKILDIRLAPRY
VYDFLFNIINETIRVREEKNIVRPDMVHLLIEARKEQKKVIQQKQAQNEDVSVLTKNLLTNEDI
VAQGFIFFFAGYGNVAVILSFAAYELAVHPEIQKRLQDEISHVLNENNGQLDYDAIKKMKYL
DMVLSETLRKWPPLPIIDRECTKAYQIKSDDLTIDLAPGMGVQIPIAGFHYDPKYYPNPEKFDP
ERFSDENKANMNPYVYMPFGLGPRVCVAVRFVMIETKIFLIHLLRQFDVVVTEKTPIPMVLKS
GNVIRPKDDFNLALVSRNE

>ON646436 CYP9GN2 [398 AA] TRINITY_DN51723_c1_g4_i2|m.57107
LFVLKDKKWRDMRNILTPVFTGSKLRGLVQLISNCGEKTVNHLVEELKEQNTKLLKVDIMDT
STRFTNDVIATSAFGIEVNSIKDPKNDFFMAGREIIRFPMWRFFMMFFLPAVSKILDIRLAPRY
VYDFLFNIINETIRVREEKNIIRPDMVHLLIEARKEQKKVIQQKQAQNEDVSVLTKNLLTNEDI
VAQGFIFFFAGYGNVAVILSFAAYELAVHPEIQKRLQDEISQVLNENNGQLDYDAIKKMKYL
DMVLSETLRKWPPLPIIDRECTKAYQIKSDDLTIDLAPGMGVQIPIAGFHYDPKYYPNPEKFDP
ERFSDENKANMNPYVYMPFGLGPRVCVAVRFVMIETKILLIHLLRQFDVVVTEKTPIPMVLK
SGNVIRPKDDFNLALVSRNE

>ON646437 CYP9GN2 [371 AA] TRINITY_DN51723_c1_g4_i3|m.57109
LFVLKDKKWRDMRNILTPVFTGSKLRGLVQLISNCGEKTVNHLVEELKEQNTKLLKVDIMDT
STRFTNDVIATSAFGIEVNSIKDPKNDFFMAGREIIRFPMWRFFMMFFLPAVSKILDIRLAPRY
VYDFLFNIINETIRVREEKNIIRPDMVHLLIEARKEQKKVIQQKQAQNEDVSVLTKNLLTNEDI
VAQGFIFFFAGYGNVAVILSFAAYELAVHPEIQKRLQDEISQVLNENNGQLDYDAIKKMKYL

DMVLSETLRKWPPLPIIDRECTKAYQIKSDDLTIDLAPGMGVQIPIAGFHYDPKYYPNPEKFDP
ERFSDENKANMNPYVYMPFGLGPRVCVAVRFVMIETKILLIHLLRQFDRNIKLSQ

>ON646438 CYP9GN2 [508 AA] TRINITY_DN51806_c0_g4_i1|m.57639
MKIPIYLYFLIHKMILELIGVLIVIYLLHLYFMKGYDYWEKRGIPSYRRFHPYIEFLNMLFQRTT
MYDDIIGVYNGLPDKKVHGINILKIPQVFVKDPDILRNICVKDFDHFNNHVDIFLQADPLFGKS
LFVLKDKKWRDMRNILTPVFTGSKLRGLVQLISNCGEKTVNHLVEELKEQNTKLLKVDIMDT
STRFTNDVIATSAFGIEVNSIKDPKNDFFMAGREIIRFPMWRFFMMFFLPAVSKILDIRLAPRY
VYDFLFNIINETIRVREEKNIIRPDMVHLLIEARKEQKKVIQQKQAQNEDVSVLTKNLLTNEDI
VAQGFIFFFAGYGNVAVILSFAAYELAVHPEIQKRLQDEISHVLNENNGQIDYDAIKKMKYLD
MVLSETLRKWPPLPIIDRECTKAYQIKSDDLTIDLAPGMGVQIPIAGFHYDPKYYPNPEKFDPE
RFSDENKANMNPYVYMPFGLGPRVCVAVRFVMIETKIFLIHLLRQFDVVVTEKTPIPMVLKS

>ON646439 CYP9GN2 [495 AA] TRINITY_DN51806_c0_g4_i2|m.57641
MILELIGVLIVIYLLHLYFMKGYDYWEKRGIPSYRRFHPYIEFLNMLFQRTTMYDDIIGVYNG
LPDKKVHGINILKIPQVFVKDPDILRNICVKDFDHFNNHVDIFLQADPLFGKSLFVLKDKKWR
DMRNILTPVFTGSKLRGLVQLISNCGEKTVNHLVEELKEQNTKLLKVDIMDTSTRFTNDVIAT
SAFGIEVNSIKDPKNDFFMAGREIIRFPMWRFFMMFFLPAVSKILDIRLAPRYVYDFLFNIINET
IRVREEKNIIRPDMVHLLIEARKEQKKVIQQKQAQNEDVSVLTKNLLTNEDIVAQGFIFFFAGY
GNVAVILSFAAYELAVHPEIQKRLQDEISHVLNENNGQIDYDAIKKMKYLDMVLSETLRKWP
PLPIIDRECTKAYQIKSDDLTIDLAPGMGVQIPIAGFHYDPKYYPNPEKFDPERFSDENKANMN
PYVYMPFGLGPRVCVAVRFVMIETKIFLIHLLRQFDVVVTEKTPIPMVLKS

>ON646440 CYP9GN1 [414 AA] TRINITY_DN51806_c0_g2_i1|m.57636
MLFVIIGILIALYLLHLYFMKGQDYWKKRGVPTYQRSHPYKEFIRLILQKTSIYEDFISLYNGFP
DKKVYGVNAFNVPQLVVKDPEIIKNICVKDFDHFVNHIDVFFKADPLFGRSLFAIKDKKWRD
MRNILSPVFTGSKLRGLVQLISNCGEQTVKYLTEKCKEQDNKLLKVDLLDMTTRFTNDVIAT
AAFGIEVNSIQNPNNEFFEAGREIIKFPAWRVLIMFFLPMIAKIFEIRLAPKYVYNFLYNIINETI
RVREEKHIVRPDMVHLLVEGRKEQKRMLEEKSAKNEDVSSLTNSLLTNDDIVAQGFIFFLAG
YGNVAIIMSFAAYELAVQPELQKRLQDEIAHTLKENNGKLDYEVIKKMKYLDMVISETLRK
WTPVTFIDRECTKAYQIKSDDLTVDVEPGMAITIPT

>ON646441 CYP9GN1 [458 AA] TRINITY_DN51806_c0_g2_i2|m.57637
MLFVIIGILIALYLLHLYFMKGQDYWKKRGVPTYQRSHPYKEFIRLILQKTSIYEDFISLYNGFP
DKKVYGVNAFNVPQLVVKDPEIIKNICVKDFDHFVNHIDVFFKADPLFGRSLFAIKDKKWRD
MRNILSPVFTGSKLRGLVQLISNCGEQTVKYLTEKCKEQDNKLLKVDLLDMTTRFTNDVIAT
AAFGIEVNSIQNPNNEFFEAGREIIKFPAWRVLIMFFLPTIAKIFEIRLAPKYVYNFLYNIINETIR
VREEKHIVRPDMVHLLVEGRKEQKRMVEEKSAKNEDMPSLTNSVLTNDDIVAQGFIFFLAGY
GNVAIIMSFAAYELAVQPELQKRLQDEIAHTLKENNGKLDYDVIKKMKYLDMVIAETLRKW
TPVTFIDRECTKAYQIKSDDLTVDVEPGMAITIPTVGFHYDPKYFPNPNKFDPERFSDENKER
MNPYVYMPFGLGPRVC

>ON646442 CYP4XE3 [500 AA] TRINITY_DN52387_c0_g1_i2|m.60175
MLPLITFGLFIVVCLCIYCYQLSIRNKHKSIPCPPLVPVLGNCFEFSSSLEGLYFDILRLQRKYGK
LFQIILGPFKHVFVVFDADLMEKVMSTSKHSDKSMEYTFFKPWLGNGLLISSGSLWRKRRKM
LTPAFHFTILQQAVDKFDENVNILVKNLEKYATTNEVFDIYPHVTLCVLDIICETAMDIKINAQ
TDSNSKYVCAVRNACCALQKRFFSVVDQSDFFFNNFSRTGKMYNDSLKILHELSNKVIVERR
EALIRRQSNYLINNEDFYKPKMIFLDTLLESTIDGRLLTNAEIREEVDTFMFEGHDTVSSAITFT

LYLLANHPLVQDRLFAEIKEVLGTDPSKPATLNDLNNLNYIELVIKESLRLYPPVPLVGRTLTE
DLQWGDVSLSKGSQIVVVIHGAHRHEDYFPEPEVFDPERFLNNTSNKNAFAYVPFSAGPRNCI
GQKFAMYEMKSTISKILRYYELESAGPGYDLKLIMTVIFKSMNGVHLKLKPRIY

>ON646443 CYP4236B1 [359 AA] TRINITY_DN52543_c2_g3_i1|m.61016
MQRQAISPIFTPKMMTEYMPSFSNHCELLKRKLQSIPNGKLIDVYAEIAPFTYDIVCETLLGTK
VRFAQNTWKSEFFHLAELGYEIVFERMIKSLYQSDVLFYFSKRYNDYVKIQKLVDNFTRTILK
DKQSDTQKPFDTCLDRILKHDLTEKQQMDATYVIFTASQESITTIISFVILMLAIHPDIQQKAHE
ELDRVLGTEDDEIPFTIELLNSLEYLDMCIKEVLRLYPIGHIIQRECKQDFRLNNDVVLPSGCSI
GVGIYAMHRDPNIWPEPDKFIPERFTREAVLNRHPYAYVPFSAGPMNCIGKIYADLLMKSVA
ATILRNNRFECDYKRVEEITLKTDISIRPQKGYPVRIYSR

>ON646444 CYP6YL1 [456 AA] TRINITY_DN52759_c6_g1_i2|m.61627
MALVVSLTIVLVVILFISVYCYVIRNFDYWRKRRIPYIEPTPFFGNIGSVFRCKESIGQCIATLHK
QTNEPFVGIFTVDCPALLIRDLELMRHILIKDFNSFTDRNFAPDPEIEPLASHGLFCLTGDKWR
NLRGKLTSAFSISRIKNMFYLVEQCAEPMNKILTKHAENNEPLDVKDFMARYTTDVISTCVFG
VTGNAINDPNWDFYHIGRSVFKTSPLDSVKSGLLIFAPWFVKLFGIRMINDSIYDAIRKSIWTAI
DLRQKNNIIRNDFIDLLLQIREKSLAEDRRNDESVLFLDGDALVGQAFVFLLGGYETSSTTMS
LALYEMALNPVIQQKVRREIDRVLAKHNNTMSYEAVHEMTYLNQVVSETLRKYPALPFLDR
NCVKDYIIPGTNVKIDCGTKVFISLLSLHHDPEIFPNPEQFDPDRFTEENISKRHKFAYLPFGNG
PRICIGKFH

>ON646445 CYP6YL1 [532 AA] TRINITY_DN52759_c6_g2_i2|m.61628
LIGHNGKWLNVPKGLNTCYNFLTKMALVVSLTIVLVVILFISVYCYVIRNFDYWRKRRIPYIE
PTPFFGNIGSVFRCKESIGQCIATLHKQTNEPFVGIFTVDCPALLIRDLELMRHILIKDFNSFTDR
NFAPDPEIEPLASHGLFCLTGDKWRNLRGKLTSAFSISRIKNMFYLVEQCAEPMNKILTKHAE
NNEPLDVKDFMARYTTDVISTCVFGVTGNAINDPNWDFYHIGRSVFKTSPLDSVKSGLLIFAP
WFVKLFGIRMINDSIYDAIRKSIWTAIDLRQKNNIIRNDFIDLLLQIREKSLAEDRRNDESVLFL
DGDALVGQAFVFLLGGYETSSTTMSLALYEMALNPVIQQKVRREIDRVLAKHNNTMSYEAV
HEMTYLNQVVSETLRKYPALPFLDRNCVKDYIIPGTNVKIDCGTKVFISLLSLHHDPEIFPNPE
QFDPDRFTEENISKRHKFAYLPFGNGPRICIGMRFGLLQTKVGLIYLLKNFEIEPLLETMKPIEF
TSTNITLAIKHPLKLRIKKRTD

>ON646446 CYP4416A4 [487 AA] TRINITY_DN52793_c3_g2_i4|m.62078
WNYIRMCYYASKLPGPPAYPIIGNAKLFLGSHQEVTNKLINIAEKYQNMVRVWIGPVPLIGSS
NPDIVQKLFTNEHVLEKAYLLKLVWNPVMGKSVFTSEVTEWKHHRSIVIRGFTPIILKSYFKIF
VEKINILIQKLDLELNKETPFDIYKYLSRTTLDSVCGSTMGVNINSHDDDGSSSYFHAINSVTFF
ILARFLNPIKYPTIFYRFTQDYKMVSKSRDVTQEFTEKIISEKRKSMATLTNNNELMDIKAINN
NAYKKPFLEYLMDFTKNNSNFTEEQLHNETNVLIFAGYETTAITLSFVLLHMAAYKDIQTKL
YNEIIEVLGDKNVDSINVDDLPKLKYMEMVLKESLRLTPTVPLIPREASQDIKIDNYTIPKGAN
ILIPIINIHRNPEYWENPLKFNPERFTSENIKNRHPYAYLPFGAGPRSCLGRRYAWIFMKTALV
ALLSRYEFHTDINLDTLRFEAGMALKLVNGYPVRITPRYKS

>ON646447 CYP4416A2 [537 AA] TRINITY_DN52793_c3_g2_i6|m.62081
MLDMGCNIYVGAELFLKNTMFLTVFIQFLQNHVLVSLLFLILVLTCIYFWNYIRVCYYASKIP
GPPAYPIIGNAIFLLGLSHQEFTHAILKIAEDYQQNLGRIWFGPIPAIGASNPDIVQELLTNENVL
EKAYLMKKAWNPVLGKSILTSEVPEWKHHRSIIIRGFTPIILKSYFKIFVEKINILIQKLDLELNK
ETPFDIFEYLSRTTLDSVCGSTMGVNINSHEDDGSSYFHAVNSSLYLVVAPYLNPIKFFNIFYR
FSQDYKMVLKNRDLTQEFTKKIISEKQKLMANVINNNELNNIKAINRNVYKKPFLEYLMDFT
KNNSNFTEEQLHNETNVLIFAGYETTAITLSFVLLHMAAYKDIQTKLYNEIIEVLGDKNVDSIN

VDDLPKLKYMEMVLKESLRLTPTVPLIPREASQDIKIDNYTIPKGANILIPIINIHRNPEYWENP
LKFNPERFTSENIKNRHPYAYLPFGAGPRSCLGRRYAWIFMKTALVALLSRYEFHTDINLDTL
RFEAGMALKLVNGYPVRITPRYKS

>ON646448 CYP4416A3 [536 AA] TRINITY_DN52793_c3_g2_i13|m.62083
MLDMGCNIYVGAELFLKNSMFLMVFMNFLQNHVLISLLLLILILTCIYFWNYIRVCYYVSKIP
GPPAYPIIGNAKLFWGSHAEVTHNLINMAEKYRNMVRVWIGPVPLIGSSNPDIVQKLFTNEHV
LEKAYLLKLVWNPVMGKSVFTSEVTEWKHHRSIVIRGFTPIILKSYFKIFVEKINILIQKLDLEL
NKETPFDIYKYLSRTTLDSVCGSTMGVNINSHDDDGSSSYFHAINSVTFFILARFLNPIKYPTIF
YRFTQDYKMVSKSRDVTQEFTEKIISEKRKSMATLTNNNELMDIKAINNNAYKKPFLEYLMD
FTENNSSFTEEQLRNHTNLLIFGGYETTAISLSFVLLHLAAYKDIQTKLYNEIIEVLGDINVDLI
NVDDLPKLKYMDMVLKESMRLTPTTPVISREASQDIKIDNYTIPKGANILIPIINIHRNPEYWE
NPLKFNPERFTSENIKNRHPYAYLPFGAGPRSCLGRRYAWIFMKTALVALLSRYEFHTDINLD
TLRFEAGMALKLVNGYPVRITPRYKS

>ON646449 CYP6YH1 [523 AA] TRINITY_DN52853_c3_g1_i1|m.62169
MAIITSNWNTDLLTLLAVVLTIIYFYFKSTFNYWKKRGVPYSEPNWIFGNFYDAITFKKNITSY
FCEQYHKTDEKAYGLYIFQRPYLLLRDPELIKHVLVKDFSNFIPRTSADVKGDEIGRHNMFSM
KSVSGWRYIRSKLSPFFTSGKMKNMFGLLDQIGDQLTQQVKSHVKESLDAKEIARLYTTDVI
VSTAYGIEVNSFSDKESVFSKVGKMMITFDLRRSLEFLCIFFCPIMVKIFNFKLFSKVGSDFLRN
VVMNAMAEREANNIKRPDLIEALITLKNKGTLEDSNKTGDIVANGNNVKNDLPPFKLEGDIL
VAQAAVFFTAGFETTSSAISFTLYSLSYNPEIQIKLRKEIQAVIARNGGNFTYDCLKDMKYLDA
CVKETLRLYPTLGFLDREAIDTYTFPGTNITIDKGTAVVISLEALHRDPQYFENPLVYDPERFM
EDKNIVPYTYMPFGEGPRNCIGARFGMMSTKVGLVHILKDFEVLTHKTAPPLEIDNKGFLLTA
KNGINLKFVPGTVYTE

>ON646450 CYP6YH1 [406 AA] TRINITY_DN52853_c3_g1_i6|m.62170
MAIITSNWNTDLLTLLAVVLTIIYFYFKSTFNYWKNRGVAYSEPNWIFGNFYDAITFKKNITSY
FCEQYNKTDEKAYGLYIFQRPYLLLRDPELIKHVLVKDFNNFVPRTSADIKGDEIGRHNMFSM
KSVSGWRYIRSKLSPFFSSGKMKNMFGLLDQIGDQLTQQVKSHVKESLDAKEIARLYTTDVI
VSTAYGIEVNSFSDKESVFSKVGKMMITFDLRRSLEFLCIFFCPIMVKIFNFKLFSKVGSDFLRN
VVMNAMAEREANNIKRPDLIEALITLKNKGTLEDSNKTGDIVANGNNVKNDLPPFKLEGDIL
VAQAAVFFTAGFETTSSAISFTLYSLSYNPEIQIKLRKEIQAVIARNGGNFTYDCLKDMKYLDA
CVKETLRLYPTLGFLDREAIDTYTFP

>ON646451 CYP6YH3 [436 AA] TRINITY_DN52853_c3_g1_i7|m.62171
MAIITSNWNTDLLTLLAVVLTIIYFYFKSTFNYWKKRGVPYSEPNWIFGNFYDAITFKKNITSY
FCEQYHKTDEKAYGLYIFQRPYLLLRDPELIKHVLVKDFSNFIPRTSADVKGDEIGRHNMFSM
KSVSGWRYIRSKLSPFFTSGKMKNMFGLLDQIGDQLTEQVKSHVNDSLDAKEIARFYTTDVI
VSAAFGIQANSFSDKEAAFSKVGKFMFTMNLRRSLELLCIFFWPIIAKIFNFKLFSKVGSDFLR
NVVMNAMAEREANNIKRPDLIEALITLKNKGTLEDSNKTGDIVANGNNVKNDLPPFKLEGDI
LVAQAAVFFTAGFETTSSAISFTLYSLSYNPEIQIKLRKEIQAVIARNGGNFTYDCLKDMKYLD
ACVKETLRLYPTLGFLDREAIDTYTFPGTNITIDKGTAVVISLEALHRDPQYFENPL

>ON646452 CYP6YH2 [440 AA] TRINITY_DN52853_c3_g1_i10|m.62174
MAIITSNWNTDLLTLLAVVLTIIYFYFKSTFNYWKKRGVPYSEPNWIFGNFYDAITFKKNITSY
FCEQYHKTDEKAYGLYIFQRPYLLLRDPELIKHVLVKDFSNFIPRTSADVKGDEIGRHNMFSM

KSVSGWRYIRSKLSPFFTSGKMKNMFGLLDQIGDQLTQQVKSHVKESLDAKEIARLYTTDVI
VSTAYGIEVNSFSDKESVFSKVGKMMITFDLRRSLEFLCIFFCPIMVKIFNFKLFSKVGSDFLRN
VVMNAMAEREANNIKRPDLIEALIALKNKGTLEDSNKTEDGVANGITSKDDKPPFKLEGDVL
VAQAAIFFTAGFESSSSAISFTLHSISYNPEIQTRLRKEIQTVISRNGGKFTYDCLKDMKYLDAC
VKETLRLYPTLGFLDREAIDTYTFPGTNITIDKGTAVVISLEGLHRDPQYFENPLIYDP

>ON646453 CYP6YH2 [440 AA] TRINITY_DN52853_c3_g1_i15|m.62177
MAIITSNWNTDLLTLLAVVLTIIYFYFKSTFNYWKKRGVPYSEPNWIFGNFYDAITFKKNITSY
FCEQYHKTDEKAYGLYIFQRPYLLLRDPELIKHVLVKDFSNFIPRTSADVKGDEIGRHNMFSM
KSVSGWRYIRSKLSPFFTSGKMKNMFGLLDQIGDQLTEQVKSHVNDSLDAKEIARFYTTDVI
VSAAFGIQANSFSDKEAAFSKVGKFMFTMNLRRSLELLCIFFWPIIAKIFNFKLFSKVGSDFLR
NVVMNAMAEREANNIKRPDLIEALIALKNKGTLEDSNKTEDGVANGITSKDDKPPFKLEGDV
LVAQAAIFFTAGFESSSSAISFTLHSISYNPEIQTRLRKEIQTVISRNGGKFTYDCLKDMKYLDA
CVKETLRLYPTLGFLDREAIDTYTFPGTNITIDKGTAVVISLEGLHRDPQYFENPLIYDP

>ON646454 CYP4416A5 [395 AA] TRINITY_DN52979_c0_g1_i5|m.62504
VLISLLLLILILTCIYFWNYIRVCYYASKIPGPPAYPIIGNAIFLLGLSHQEFTHAILKIAEDYQQN
LGRIWFGPIPAIGASNPDIVQELLTNENVLEKAYLMKKAWNPVLGKSILTSEVPEWKHHRSIII
RGFTPIILKSYFKIFVEKINILIQKLDLELNKETPFDIFEYLSRTTLDSVCGSTMGVNINSHEDDG
SSYFHAINSAIFFIIARLLNPIKLPSILYRFTRDYKMVSRSRDVTHEFTKKIISEKRKNKATVTNN
KELMDIKAINSNLYKKPFLEYLMDFTENNSSFTEEQLRNETNFMIMAGYDTTALSLSYVLLHL
AAYKDIQTKLYNEILEVVGDINVDLINVDDLPKLKYMEMVLKESMRLTPTIPLIVREPSQDIKI
GNICL

>ON646455 CYP4416A5 [505 AA] TRINITY_DN52979_c0_g1_i6|m.62506
VLISLLLLILILTCIYFWNYIRVCYYASKIPGPPAYPIIGNAIFLLGLSHQEFTHAILKIAEDYQQN
LGRIWFGPIPAIGASNPDIVQELLTNENVLEKAYLMKKAWNPVLGKSILTSEVPEWKHHRSIII
RGFTPIILKSYFKIFVEKINILIQKLDLELNKETPFDIFEYLSRTTLDSVCGSTMGVNINSHEDDG
SSYFHAINSAIFFIIARLLNPIKLPSILYRFTRDYKMVSRSRDVTHEFTKKIISEKRKNKATVTNN
KELMDIKAINSNLYKKPFLEYLMDFTENNSSFTEEQLRNETNFMIMAGYDTTALSLSYVLLHL
AAYKDIQTKLYNEILEVVGDINVDLINVDDLPKLKYMEMVLKESMRLTPTIPLIVREPSQDIKI
GNYIIPKGASILIPIFTIHRNPEYWENPLKFDPERFTAENIKNRHPYAYIPFSAGPRNCIGGRYA
WIFMKTTLAALLSRYEFHTDINLDTLKFEAAITLKLVNGHQVRITPRYKS

>ON646456 CYP4416A5 [399 AA] TRINITY_DN52979_c0_g1_i13|m.62511
VLISLLLLILILTCIYFWNYIRVCYYASKIPGPPAYPIIGNAIFLLGLSHQEFTHAILKIAEDYQQN
LGRIWFGPIPAIGASNPDIVQELLTNENVLEKAYLMKKAWNPVLGKSILTSEVPEWKHHRSIII
RGFTPIILKSYFKIFVEKINILIQKLDLELNKETPFDIYKYLSRTTLDSVCGSTMGVNINSHDDD
GSSSYFHAINSVTFLILARCLNPIKYPTIFYRFTQDYKMVSKSRDVTQEFTEKIISEKRKSMATL
TNNNELMDIKAINNNAYKKPFLEYLMDFTKNNSNFTDEQLRNETNFMIMAGYDTTALSLSY
VLLHLAAYKDIQTKLYNEIIEVVGDINVDLINVDDLPKLKYMEMVLKESLRLTPTVPLIPREAS
QDIKIDNYTIPKG

>ON646457 CYP4416A5 [505 AA] TRINITY_DN52979_c0_g1_i22|m.62520
VLISLLLLILILTCIYFWNYIRVCYYASKIPGPPAYPIIGNAIFLLGLSHQEFTHAILKIAEDYQQN
LGRIWFGPIPAIGASNPDIVQELLTNENVLEKAYLMKKAWNPVLGKSILTSEVPEWKHHRSIII
RGFTPIILKSYFKIFVEKINILIQKLDLELNKETPFDIFEYLSRTTLDSVCGSTMGVNINSHEDDG

SSYFHAINSAIFFIIARLLNPIKLPSILYRFTRDYKMVSRSRDVTHEFTKKIISEKRKNKATVTNN
KELMDIKAINSNLYKKPFLEYLMDFTENNSSFTEEQLRNETNFMIMAGYDTTALSLSYVLLHL
AAYKDIQTKLYNEILEVVGDINVDLINVDDLPKLKYMEMVLKESMRLTPTIPLIVREPSQDIKI
GNYIIPKGASILIPIFTIHRNPEYWENPLKFDPERFTAENIKNRHPYAYIPFSAGPRNCIGGRYA
WIFMKTALVALLSRYEFHTDINLDTLRFEAGMALKLVNGYPVRITPRYKS

>ON646458 CYP4416A6 [439 AA] TRINITY_DN52979_c0_g1_i14|m.62512
RNMIRVWIGPVPVIGSSNPEIVQELLTNEYVLEKGYLLKFVWNPVMGKSIFTSEVPEWRNHRR
IVIRGFTPIILKSYFKIFVEKINILIQKLDLELNKETPFDIYKYLSRTTLDSVCGSTMGVNINSHDD
DGSSSYFHAINSVTFFILARFLNPIKYPTIFYRFTQDYKMVSKSRDVTQEFTEKIISEKRKSMAT
LTNNNELMDIKAINNNAYKKPFLEYLMDFTENNSSFTEEQLRNHTNLLIFGGYETTAISLSFVL
LHLAAYKDIQTKLYNEIIEVLGDINVDLINVDDLPKLKYMDMVLKESMRLTPTTPVISREASQ
DIKIDNYTIPKGANILIPIINIHRNPEYWENPLKFDPERFTAENIKNRHPYAYIPFGAGPRNCFGR
RYAWIFMKATLVALLCRYEFHTDINLDTLKFEIGMALKLINGHQVQITPRY
